# Promoting π-Facial Interactions in Phenyl-Substituted 1,8-Bis(silylamido)naphthalene Alkaline Earth Complexes

**DOI:** 10.1021/acs.organomet.4c00479

**Published:** 2025-02-05

**Authors:** Matthew D. Haynes, Clement G. Collins Rice, Louis J. Morris, Zoë R. Turner, Dermot O’Hare

**Affiliations:** Chemistry Research Laboratory, Department of Chemistry, University of Oxford, 12 Mansfield Road, OX1 3TA Oxford, U.K.

## Abstract

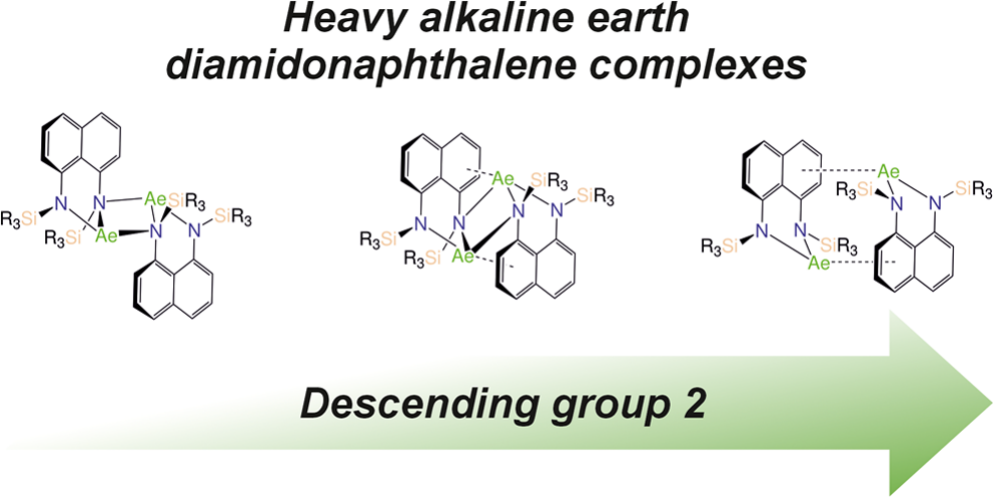

Bimetallic 1,8-bis(silylamido)naphthalene alkaline earth complexes [(^R_3_^L)Ae]_2_ ([^R_3_^L]^2–^ = [1,8-{(R_3_Si)N}_2_C_10_H_6_)]^2–^, where R_3_ = Ph_2_Me, Ae = Ca (**1**), Sr (**2**), and Ba (**3**); R_3_ = Ph_3_, Ae = Ca (**4**), Sr (**5**), and Ba (**6**) were prepared *via* protonolysis reactions of the phenyl-substituted proligands ^Ph_3_^LH_2_ and ^Ph_2_Me^LH_2_ with [AeN″_2_]_2_ (N″ = [N(SiMe_3_)_2_]^−^) in benzene. X-ray crystallographic analysis showed that **1**, **2**, and **4** crystallize as nitrogen-bridged dimers. Conversely, **5** and **6** display a naphthalene-bridged motif, while the structure of **3** is intermediate between the two distinct classes. NMR spectroscopic analysis of isolated samples of **1**–**6** in thf-*d*_8_ confirmed their conversion into the monomeric thf-*d*_8_ adducts [(^R_3_^L)Ae(thf-*d*_8_)_*n*_]; crystallographic verification of the structural motif was provided by the X-ray crystal structure of [(^Ph_3_^L)Sr(thf)_3_] (**7**). The structural range of dimers **1**–**6** was influenced by the electron-withdrawing nature of the phenyl substituents of the ligand and the ability to form “soft” multihaptic π-facial interactions with the metal ions, which was preferential for the larger Sr^2+^ and Ba^2+^ cations as well as the relative strength of the metal-N bonds. This has been rationalized through complementary computational studies. This work provides insight into the structure and bonding preferences of heavy alkaline earth complexes with rigid bis(amido) ligands.

## Introduction

The chemistry of the alkaline earth (Ae) elements magnesium, calcium, strontium, and barium has been developed substantially in recent years. These metals are attractive from a sustainability perspective due to their high terrestrial abundance, low cost, and (with the exception of Ba^2+^, which interferes with transmembrane K^+^ channels) low toxicity.^1^ However, until relatively recently, the application of coordination complexes of these electropositive elements to the fields of small molecule activation and catalysis has been limited as a consequence of their propensity for ligand redistribution *via* Schlenk equilibria.^2^ Much research has therefore been devoted to the search for suitable ligand systems that are able to form well-defined coordination complexes with these elements, the bonding of which is predominantly ionic in character. Many of these are bulky, bi- or multidentate monoanionic nitrogen-based ligands ([L]^−^).^3^ These are able to strongly coordinate to the highly ionic Ae^2+^ dications through their hard nitrogen donors, forming a defined pocket flanked by a peripheral steric bulk. This enables the coordination of smaller monodentate ligands ([X]^−^) in a controlled manner to form reactive heteroleptic species of the form LAeX.

In contrast, homoleptic alkaline earth complexes bearing dianionic ligands ([L]^2–^) have received less attention. However, various reports have described alkaline earth complexes featuring dianionic ligands such as reduced α-diimines,^2a,4^ bora-amidinates,^5^ and bis(phosphinosulfonicamides)^6^ that confer onward reactivity through noninnocent behavior. A number of recent reports have also exploited alkaline earth complexes featuring chelating diamido ligands, including ethylene-bridged bis(silylamide)s,^7^ di(amido)siloxanes,^8^ and rigid NON-Xanthene ligands,^9^ as precursors for the synthesis of reactive heterometallic species.^10^

**Chart 1:**
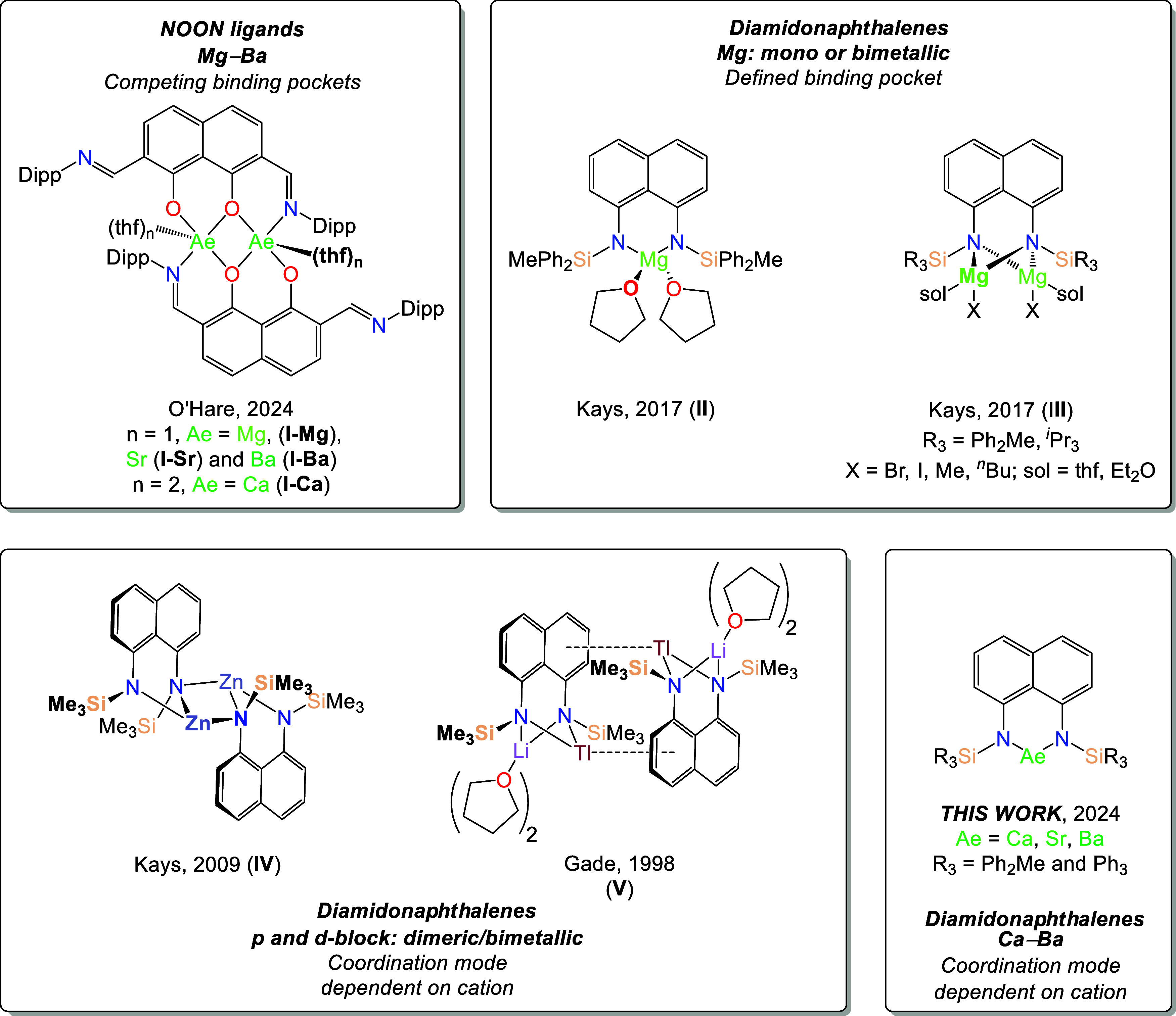
Selected Examples of Complexes Supported by Dianionic Chelating Ligands with Rigid Naphthalene Backbones

We have recently reported a series of dimeric alkaline earth complexes supported by a bis(phenoxyimine) “NOON” ligand, which also contains a rigid naphthalene backbone (**I-Ae**, Ae = Mg, Ca, Sr, and Ba, Chart 1).^11^ The X-ray crystal structures of **I-Ae** revealed a central Ae_2_O_2_ core, with Ae^2+^ also coordinated by one imine donor of the NOON ligand and thf. When dissolved in thf, these dimeric species were found to exist in equilibrium with their monomeric analogues, which could be isolated as 18-crown-6 adducts. The ability of **I-Ae** (Ae = Ca, Sr, and Ba) to catalyze the ring-opening polymerization of lactide both in the presence and absence of a co-initiator was also demonstrated.^12^ These investigations demonstrated the complications arising from the presence of multiple binding pockets within the NOON ligand framework.

Consequently, we turned our attention to the synthesis of lower-coordinate complexes of the alkaline earth elements supported by 1,8-bis(silylamido)naphthalene ligands, ([^R_3_^L]^2–^ = [1,8-{(R_3_Si)N}_2_C_10_H_6_]^2–^). These bidentate bis(amido) ligands have a defined metal-binding pocket and can be accessed *via* deprotonation of the corresponding proligands ^R_3_^LH_2_. The latter are straightforwardly synthesized from 1,8-diaminonaphthalene and a wide array of commercially available alkyl- and arylsilyl chlorides, thus enabling the facile modulation of both their steric and electronic properties.^13^ They have been utilized to support a variety of p- and d-block complexes, ranging from mono and bimetallic monomeric species to bimetallic dimers.^14^

Despite this, there have been relatively few reports of s-block complexes bearing 1,8-bis(silylamido)naphthalene ligands, excluding dilithium salts, which have often been employed as salt metathesis reagents.^13,14c,15^ A mixed Mg/Li complex has been reported,^15b^ while in 2017, Kays and co-workers reported both mono- and bimetallic Mg(II) complexes (**II** and **III**, respectively, Chart 1).^16^

A number of aggregated species supported by 1,8-bis(silylamido)naphthalene ligands have been described, the solid-state structures of which display a range of structural motifs. In 2009, Kays and co-workers reported the dimeric zinc complex [(^Me_3_^L)Zn]_2_ (**IV**, Chart 1).^14a^ This nitrogen-bridged species consists of a central Zn_2_N_2_ core in which the two [^Me_3_^L]^2–^ ligands each chelate one Zn^2+^center in a κ^2^-*N*,*N*′-bidentate manner, with a μ_2_-N donor also bridging to the other zinc dication. This motif has also been reported for ytterbium(II) and europium(II) complexes supported by a related naphthalene-bridged bis(guanidinate) ligand.^17^ Conversely, dimerization is also possible through the formation of π-facial interactions between metal cations and the naphthalene backbone; this has been reported previously for both the dilithium salt [(^Me_3_^L)Li{Li(thf)}]_2_ and the mixed thalium/lithium dimer [(^Me_3_^L)Tl{Li(thf)_2_}]_2_ (**V**, Chart 1), which result from η^3^- and η^6^-π-facial interactions, respectively.^15a^

No 1,8-bis(silylamido)naphthalene complexes of the heavier alkaline earth elements (Ae = Ca, Sr, Ba) have been reported previously, contributing to the general paucity of such s-block complexes. We therefore identified [(^R_3_^L)Ae]_*n*_ as novel synthetic targets, with a view to identifying the structural motifs adopted in both the solid state and solution phase as well as ultimately assessing their potential as precursors for reactive heterometallic complexes.

## Results and Discussion

### Synthesis of Dimeric thf-Free Alkaline Earth Complexes

Two equivalents of ^R_3_^LH_2_ (^R_3_^LH_2_ = 1,8-{(R_3_Si)NH}_2_C_10_H_6_, R_3_ = Ph_2_Me and Ph_3_) were reacted with one equivalent of the thf-free dimeric alkaline earth bases [AeN″_2_]_2_ (Ae = Mg, Ca, Sr, and Ba; N″ = [N(SiMe_3_)_2_]^−^) in benzene. The [MgN″_2_]_2_ reactions proved unproductive, likely due to (i) the poor basicity of the magnesium amide precursor precluding the deprotonation of both equivalents of proligand and (ii) the small ionic radius of Mg^2+^ preventing the formation of sterically congested aggregated products.

**Scheme 1:**
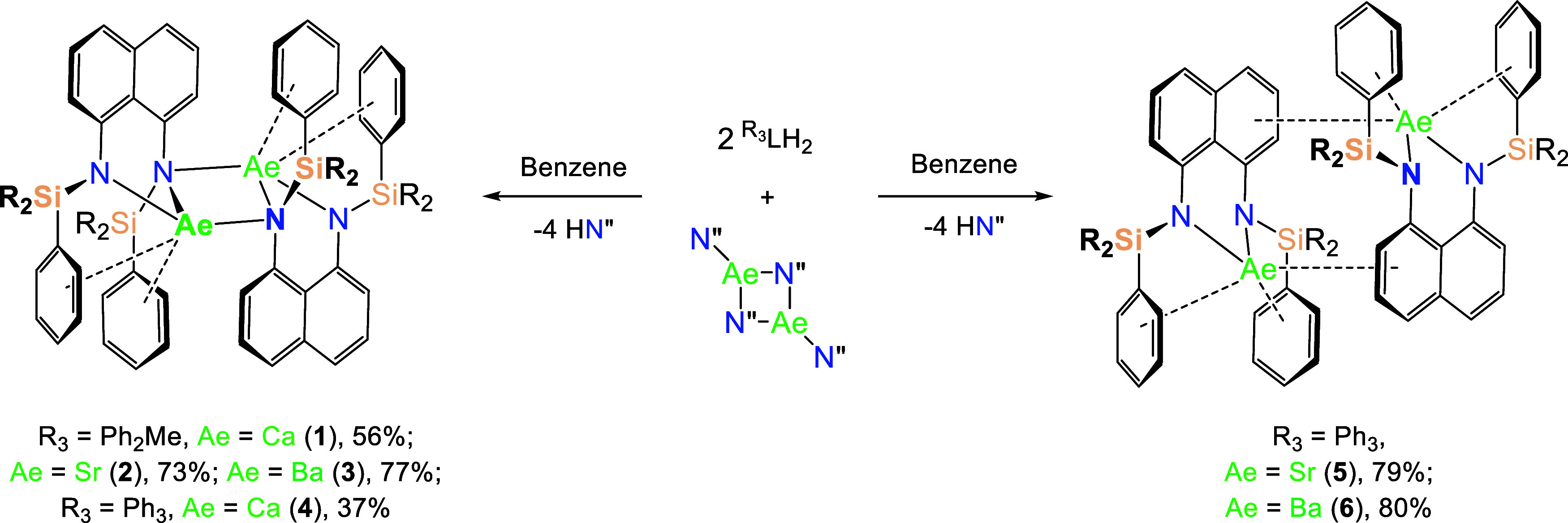
Synthesis of **1**–**6***via* the 2:1 Reaction of ^R_3_^LH_2_ (R^3^ = Ph_2_Me and Ph_3_) with [AeN″_2_]_2_ (Ae = Ca, Sr and Ba) in Benzene

However, reactions of the heavier congeners afforded the dimeric alkaline earth complexes [(^R_3_^L)Ae]_2_ (R_3_ = Ph_2_Me, Ae = Ca (**1**), 56%, Sr (**2**), 73% and Ba (**3**), 77%; R_3_ = Ph_3_, Ae = Ca (**4**), 37%, Sr (**5**), 79% and Ba (**6**), 80%) as bright yellow crystalline solids (Scheme 1). With the exception of **4**, complexes **1**–**6** all crystallize directly from the reaction mixture, enabling their facile isolation and characterization.

### Solid-State Structural Analysis: An Overview

**Scheme 2:**
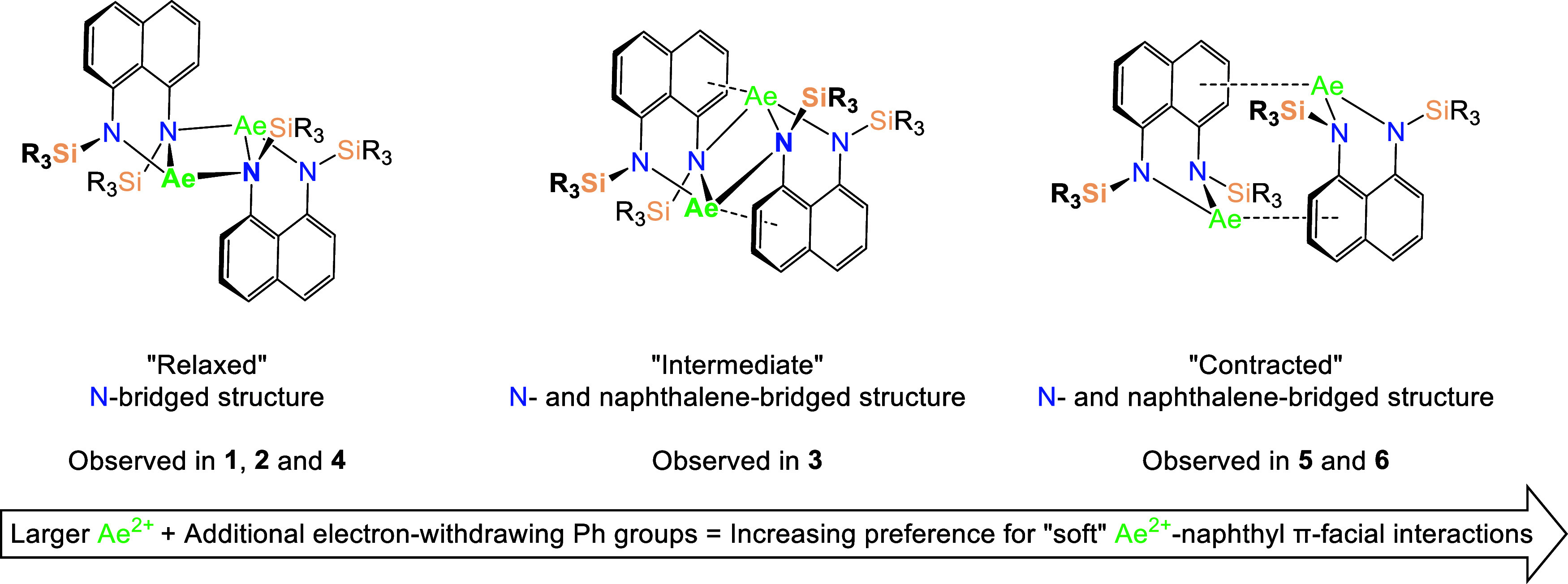
“Structural Snapshots” of the Transition of a “Relaxed” Nitrogen-Bridged [(^R_3_^L)Ae]_2_ Dimer into a “Contracted” Naphthalene-Bridged form *via* an Intermediate Species in Which Both Interactions are Present

The solid-state structures of complexes **1**–**6** exhibit either nitrogen- or naphthalene-bridged centrosymmetric dimeric motifs (Scheme 2).

### Solid-State Structural Analysis: Nitrogen-Bridged Dimers, **1**–**4**

**Figure 1:**
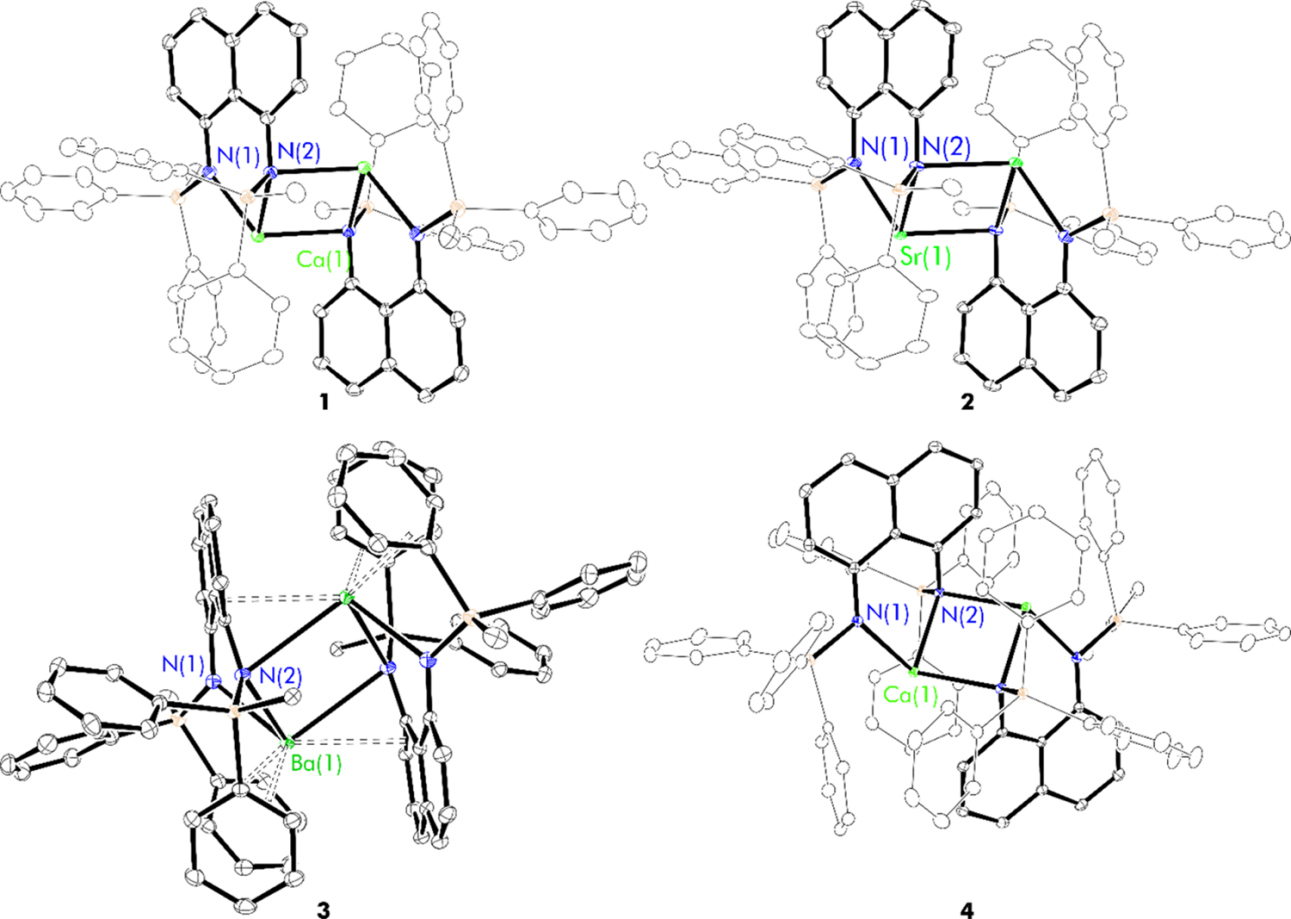
Thermal displacement ellipsoid drawings (30% probability) of [(^Ph_2_Me^L)Ca]_2_ (**1**), [(^Ph_2_Me^L)Sr]_2_ (**2**), [(^Ph_2_Me^L)Ba]_2_ (**3**), and [(^Ph_3_^L)Ca]_2_ (**4**). All hydrogen atoms have been omitted, and wireframes are used for clarity.

Complexes **1**, **2**, and **4** all crystallize as exclusively nitrogen-bridged dimers, whereas complex **3** features both N- and naphthalene bonding with the metal center. Complexes **1**–**3** crystallize in the *P*2_1_/*c* space group, with structures consisting of only a single centrosymmetric [(^Ph_2_Me^L)Ae]_2_ dimer. Complex **4** crystallizes in the *P*1̅ space group and has a structure consisting of four crystallographically distinct [(^Ph_3_^L)Ca]_2_ molecules, though there is little metrical variance between them. (Figures 1 and S19 and Table 1).

**Table 1:** Experimental Metrical Parameters (Bond Lengths in Å and Angles in °) in [(^R_3_^L)Ae]_2_ (R_3_ = Ph_2_Me, Ae = Ca (**1**), Sr (**2**), and Ba (**3**); R_3_ = Ph_3_, Ae = Ca (**4**))

	**1**	**2**	**3**^a^	**4**
space group	*P*2_1_/*c*	*P*2_1_/*c*	*P*2_1_/*c*	*P*1̅
Ae(1)–N(1)	2.308(2)	2.462(3)	2.621(2)	2.3106(13)
Ae(1)–N(2)	2.479(2)	2.489(2)	2.647(2)	2.3568(13)
Ae(1)–N(2)^a^	2.332(2)	2.679(3)	3.088(2)	2.4783(14)
Ae(1)–Ae(1)^a^	3.4870(8)	3.7726(4)	4.2476(2)	3.4486(4)
N(1)–N(2)	2.956(3)	2.983(4)	2.935(3)	2.9647(19)
Ae(1)-plane_C(1)–C(10)_	0.522(4)	0.60372(2)	1.433(4)	0.564(2)
Ae(1)-plane_C(1)–C(10)_^a^	1.809(3)	2.04097(5)	2.9616(18)	1.4283(19)
plane_C(1)–C(10)_-plane_C(1)–C(10)_^a^	2.331(6)	2.6447(1)	4.395(6)	1.992(4)
N(1)–Ae(1)–N(2)	79.14(7)	74.10(9)	67.73(7)	78.86(4)
N(1)–Ae(1)–N(2)^a^	117.04(7)	114.30(9)	104.41(7)	121.18(5)
N(2)–Ae(1)–N(2)^a^	87.14(7)	86.24(8)	84.73(6)	89.04(5)
∑{∠Ae(1)}	283.32(12)	274.70(13)	256.87(12)	289.07(7)
plane_C(1)–C(10)_-plane_Ca(1)_2_N(2)_2__ fold angle	62.354(2)	63.407(2)	95.00(9)	57.00(5)

a Refers to symmetry-related atoms.

Using [(^Ph_3_^L)Ca]_2_ (**4**) as a representative example to highlight key structural features, the asymmetric unit of **4** consists of a monomeric [(^Ph_3_^L)Ca] unit, with the full dimeric structure (which is generated by a (−*X*, −*Y* + 2, −*Z* + 1) symmetry operation) featuring a Ca_2_N_2_ diamond core. This dimerization mode has previously been reported for the 1,8-bis(silylamido)naphthalene Zn(II) complex (**IV**, Chart 1),^14a^ Eu(II)^17a^ and Yb(II)^17b^ complexes supported by a related ligand, as well as a range of alkaline earth complexes bearing both chelating diamido ligands^18^ and a related bis(phenoxyimine) ligand with a naphthalene backbone (**I-Ae**, Chart 1).^11^

The calcium centers are formally three-coordinate, chelated by one dianionic [^Ph_3_^L]^2–^ ligand in a κ^2^-*N*,*N*′-bidentate manner and bridging to the amide donor of the other ligand with a distorted trigonal pyramidal coordination geometry (∑{∠Ca(1)} = 289.07(7)°). While the bridging Ca–N bond is the longest of the three (Ca(1)–N(1) = 2.3106(23) Å < Ca(1)–N(2) = 2.3568(13) Å < Ca(1)–N(2)* = 2.4783(14) Å, where * refers to symmetry-related atoms), it is similar in length to the other Ca–N distances in this case. This reflects the strength of the ionic Ca–N bonds formed by the relatively hard Ca^2+^ cation, which results in dimerization occurring *via* the bridging μ_2_-N donors.

The [(^Ph_3_^L)Ca] units are necessarily oriented antiparallel with respect to one other. This motif results in an interplanar hinge angle of 0° between the mean planes defined by the two naphthalene backbones and a short Ca–Ca distance of 3.4486(4) Å. The Ca^2+^ center lies 0.564(2) Å from the mean plane defined by the naphthalene backbone, with the accommodation of the large Ca^2+^ cation relatively close to the plane of the [^Ph_3_^L]^2–^ ligand, necessitating a distortion of the naphthalene backbone away from planarity (C(1–10)-plane_C(1)–C(10)_ = 0.002(1)–0.170(1) Å, see Figure S20e). This results in an increase in the N–N distance of **4** (2.9647(19) Å) relative to that of proligand ^Ph_3_^LH_2_ (2.827(2) Å) as the metal-binding pocket widens to facilitate the coordination of Ca^2+^, which is consequently exposed as a result of the acute N(1)–Ca(1)–N(2) bite angle of 78.86(4)°.

Coordinative saturation is provided by the formation of both η^1^- and η^2^-π-facial interactions between Ca^2+^ and two phenyl substituents (one from each SiPh_3_ group) and the naphthalene backbone of the opposing [^Ph_3_^L]^2–^ ligand. However, Ca^2+^ does not reside directly above the opposing naphthalene ring (Ca(1)-centroid_C(5)–C(10)_ = 3.8029(8) Å). Together, these factors result in a “relaxed” ladder-like structure with both a relatively small fold angle between the respective Ca_2_N_2_ and naphthalene planes (57.00(5)°) and a short interplanar distance between the two naphthalene planes (1.992(4) Å). This can be attributed to the preference of the (relatively) small Ca^2+^ cation to maximize its interactions with the hard amide donors of the [^Ph_3_^L]^2–^ ligand rather than the softer π-system of the naphthalene backbone. This “relaxed” structure also helps to relieve steric congestion between the bulky SiPh_3_ groups. Various examples of stabilizing Ae^2+^-arene interactions have also been reported in the literature.^18a,19^ For example, dimeric alkaline earth complexes supported by chelating diamido ligands that dimerize *via* both bridging amido donors^20^ and Ae^2+^-aryl π-facial interactions^18a,20^ have also been reported recently by Jones and co-workers.

The structure of complex **1** is very similar to that of **4**, with their composition only differing due to the substitution of a phenyl group for a methyl group. This results in very similar metrical parameters, especially within their respective asymmetric units (Table 1). However, **1** has a larger Ae_2_N_2_-naphthyl fold angle (62.354(2)°) and a widened interplanar distance between the two naphthalene rings (2.331(6) Å). The resulting structural “contraction” is likely enabled by the reduced degree of steric congestion conferred by the smaller methyl substituents in **1**, which facilitates the accommodation of an enhanced Ca^2+^-naphthyl interaction, reflected by a slight shortening of the Ca(1)-centroid_C(5)–C(10)_ distance to 3.736(11) Å in **1**.

In the structure of the strontium congener **2** (Figure 1), the increased ionic radius of Sr^2+^ relative to Ca^2+^ results in lengthened Sr–N bond lengths and a more acute N(1)–Sr(1)–N(2) bite angle (74.13(8)°) as Sr^2+^ necessarily sits further from the [^Ph_2_Me^L]^2–^ ligand than Ca^2+^. While the interplanar distance between the two naphthalene rings in **2** has increased to 2.64469(7) Å, this is due to the extension of the Ae–N bonds *rather* than a large increase in the Ae_2_N_2_-naphthyl fold angle (62.354(2)° (**1**) *vs* 63.407(2)° (**2**)).

Barium complex **3** displays some key differences from those of **1**, **2**, and **4** (Figure 1). Within the asymmetric unit, the extremely large Ba^2+^ cation sits further from the plane of the naphthalene backbone (Ae^2+^-plane_C(1)–C(10)_ = 0.60372(2) Å (**2**) *vs* 1.433(4) Å (**3**), see Figure S20c,d). Consequently, the N(1)–Ba(1)–N(2) bite angle (67.73(7)°) is contracted, while the N(1)–N(2) distance in **3** (2.935(3) Å) is the shortest of the four nitrogen-bridged dimers. This reflects the more planar naphthalene backbone (C(1–10)-plane_C(1)–C(10)_ = 0.002(3)–0.077(2) Å): the Ba^2+^ cation is so large that it must sit out of plane, so the [^Ph_2_Me^L]^2–^ ligand has not distorted in order to accommodate it. The full dimeric structure of **3** is “contracted” relative to those of **1**, **2**, and **4**, as evidenced by its increased Ae_2_N_2_-naphthyl fold angle (95.00(9)°) and a naphthalene–naphthalene interplanar distance (4.395(6) Å) that is more than double that of **4** (1.992 (4) Å). The twist angle between the central Ae_2_N_2_ ring and the two naphthalene mean planes is also greatly increased (30.004(3)° (**2**) *vs* 61.87(7)° (**3**)), reflecting the more distorted structure of **3**. This results from the competing formation of a η^2^-π-facial Ba^2+^-naphthyl interaction, with Ba^2+^ lying above the opposing naphthalene ring (Ba(1)-centroid_C(5)–C(10)_ = 3.282(1) Å). All three Ba–N bond lengths (Ba(1)–N(1) = 2.621(2) Å, Ba(1)–N(2) = 2.647(2) Å, and Ba(1)–N(2)* = 3.088(2) Å) are lengthened in **3** relative to **2** reflecting the larger ionic radius of Ba^2+^. However, the bridging Ae(1)–N(2)* distance has increased by 0.411(3) Å, whereas the chelating Ae(1)–N(1) and Ae(1)–N(2) distances have each only increased by 0.160(3) Å. This reflects a weakening of the nitrogen-bridged dimerization mode that results from the softness of the large Ba^2+^ dication, which instead exhibits a greater preference for multihaptic π-facial interactions with the softer phenyl and naphthyl donors. Consequently, the structure of **3** can be viewed as somewhat intermediate between the more “relaxed” nitrogen-bridged complexes **1**, **2**, and **4** and the “contracted” naphthalene-bridged dimers **5** and **6** (*vide infra*). As such, **3** acts as a “structural snapshot” of the process that assumedly results in the conversion of intermediate nitrogen-bridged species into naphthalene-bridged dimers in some cases (Scheme 2).

**Figure 2:**
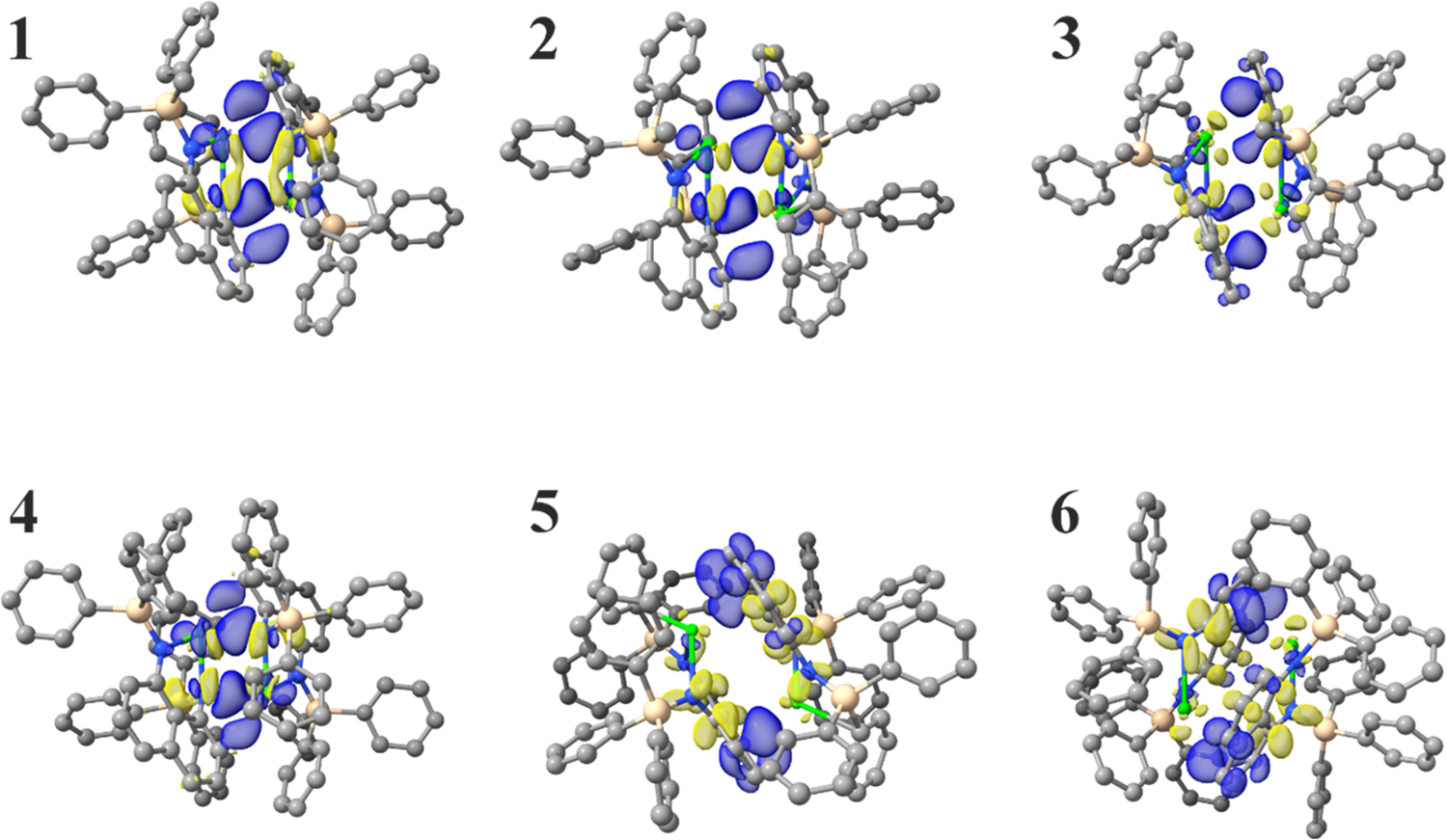
Illustrations of the deformation density of the principal interacting orbital in dimeric complexes **1**–**6** computed by EDA-NOCV relative to isolated monomeric species. Blue and yellow represent donating and accepting orbitals, respectively. The dominance of the Ae–N interaction in **1**, **2**, and **4** is contrasted with the Ae-arene π-facial interactions in **5** and **6**.

These trends are further rationalized through computational chemical methods. The interaction energies of the dimers **1**–**6** relative to isolated monomeric species were analyzed using EDA-NOCV,^21^ revealing the dominance of Ae–N donor–acceptor interactions in complexes **1**, **2**, and **4** and the more diffuse π-facial interactions in **5** and **6** as being the driving force for the observed structural preferences in these molecules (Figures 2, S21, S22, and Table S6). IGMH analysis also shows some degree of ligand–ligand van der Waals stabilization of the dimers alongside the attractive Ae-arene π interaction compensating for the repulsive effects resulting from ligand sterics (Figure S23).^22^

### Solid-State Structural Analysis: Nitrogen-Bridged Dimers, **5** and **6**

**Figure 3:**
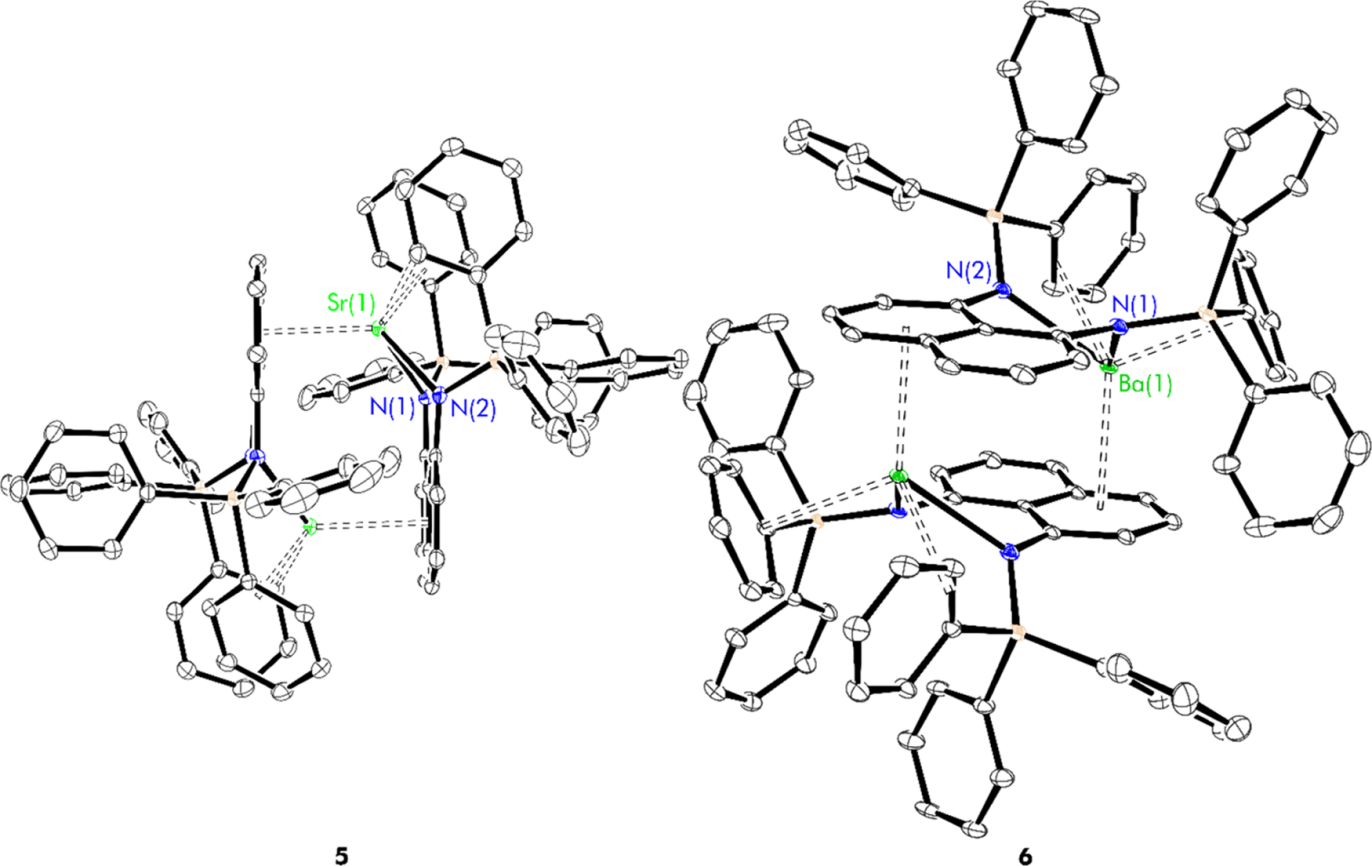
Thermal displacement ellipsoid drawings (30% probability) of [(^Ph_3_^L)Sr]_2_ (**5**) and [(^Ph_3_^L)Ba]_2_ (**6**). All hydrogen atoms have been omitted for clarity.

Like **1**–**3**, complexes **5** and **6** both crystallize in the *P*2_1_/*c* space group, with two identical [(^R_3_^L)Ae] units orientated antiparallel with respect to one another within a centrosymmetric dimeric structure. However, they display a markedly different naphthalene-bridged dimerization mode (Figure 3 and Table 2).

**Table 2:** Experimental Metrical Parameters (Bond Lengths in Å and Angles in °) in [(^Ph_3_^L)Ae]_2_ (Ae = Sr (**5**) and Ba (**6**))

	**5**	**6**
space group	*P*2_1_/*c*	*P*2_1_/*c*
Ae(1)–N(1)	2.462(2)	2.625(3)
Ae(1)–N(2)	2.445(2)	2.593(3)
Ae(1)–N(2)^a^	4.090(2)	4.276(3)
Ae(1)–Ae(1)^a^	4.8750(5)	4.9831(3)
N(1)–N(2)	2.900(3)	2.941(4)
Ae(1)-centroid_C(5)–C(10)_^a^	2.7348(9)	2.9129(12)
Ae(1)-plane_C(1)–C(10)_	1.210(3)	1.44113(1)
plane_C(1)–C(10)_-plane_C(1)–C(10)_^a^	3.944(3)	4.334402(5)
N(1)–Ae(1)–N(2)	72.47(6)	68.60(9)
N(1)–Ae(1)-centroid_C(5)–C(10)_^a^	117.31(5)	116.6599(7)
N(2)–Ae(1)-centroid_C(5)–C(10)_^a^	119.98(5)	123.3939(3)
plane_C(1)–C(10)_-plane_Ca(1)_2_N(2)_2__ fold angle	105.82(6)	106.3350(7)

a Refers to symmetry-related atoms.

Using **5** as a representative example (Figure 3), the dimeric structure consists of two symmetry-related [(^Ph_3_^L)Sr] groups. The Ae_2_N_2_ ring present in both the [SrN″_2_]_2_ precursor^23^ and the nitrogen-bridged dimers **1**–**4** has been broken. This is evidenced by a Sr(1)–N(2)* distance of 4.090(2) Å (*ca*. 1.5 Å greater than ∑*r*_cov_(Sr,N) = 2.56 Å)^24^ and the trigonal planar geometries of both nitrogen donors (∑{∠N(1)} = 358.7(2)°, ∑{∠N(2)} = 359.3(2)°).

The Sr^2+^ cation is coordinated to [^Ph_3_^L]^2–^ in a κ^2^-*N*,*N*′-bidentate manner, as well as to one phenyl substituent from each SiPh_3_ group through η^3^- and η^1^-π-facial interactions, in addition to an η^6^-π-facial interaction with the naphthalene ring of the opposing ligand. This results in a short Sr^2+^-centroid_C(5)–C(10)_ distance of 2.748(9) Å, which contrasts with the analogous distances in **1**–**3** (the shortest of which is 3.282(1) Å in **3**, which itself displays an intermediate nitrogen/naphthalene-bridged structure, *vide supra*). Consequently, **5** has a highly “contracted” dimeric structure characterized by a large Sr_2_N_2_-naphthyl fold angle (105.82(6)°) and a wide interplanar separation between the two naphthalene rings (3.944(3) Å).

The dianionic [^Ph_3_^L]^2–^ ligand is highly planar (C(1–10)-plane_C(1)–C(10)_ = 0.001(2) to 0.033(2) Å), resulting in a relatively short N(1)–N(2) distance of 2.900(3) Å. This is due to Sr^2+^ lying 1.210(3) Å from the plane of the naphthalene backbone, which consequently has not been distorted to accommodate it in-plane (Figure S20f). This notably contrasts with the asymmetric unit of the analogous [^Ph_2_Me^L]^2–^-supported strontium complex **2**, in which Sr^2+^ sits closer (Sr(1)-plane_C(1)–C(10)_ = 0.60372(2) Å) to the plane of a more distorted (C(1–10)-plane_C(1)–C(10)_ = 0.015(3)–0.100(3) Å) naphthalene backbone (Figure S20c). This variation arises from the change in dimerization mode between **2** and **5**; Sr^2+^ sits further out of plane in **5** in order to maximize its η^6^-π-facial interaction with the opposing naphthalene ring.

Complex **5** can be viewed as a dimeric “piano-stool” complex in which one face of the Sr^2+^ cation is coordinated by two nitrogen donors within the same [(^Ph_3_^L)Sr] unit, while the other face is capped by the opposing naphthalene ring. This structural motif has been previously reported for the 1,8-bis(silylamido)naphthalene-supported mixed thallium/lithium complex (**V**, Chart 1) as well as a related dilithium dimer.^15a^ Complexes **5** and **6** are also reminiscent of a series of aryl-bridged alkaline earth dimers supported by bulky chelating diamido ligands, which were reported recently by Jones and co-workers.^18a^

It is notable that simply exchanging a methyl group for an additional phenyl group results in such a marked shift in dimerization mode between the two strontium complexes **2** and **5**. In contrast, the same substitution did not result in any notable structural variation between analogous calcium complexes **1** and **4**. Though steric congestion between bulky SiPh_3_ substituents would assumedly be relieved by reducing the number of bulky amide donors coordinated to Ca^2+^, this does not preclude the accommodation of a nitrogen-bridged dimerization mode by the smaller Ca^2+^ cation in **4**. This suggests that the shift to a naphthalene-bridged structure in **5**, which contains a larger Sr^2+^ cation, cannot be solely a result of steric factors. Instead, it is proposed that the presence of an additional electron-withdrawing phenyl group reduces the charge density of the N-donor atoms. The electron-withdrawing influence exerted by phenyl groups is supported by analysis of the chemical shifts of the SiR_3_ signals in ^29^Si NMR spectra of **1**–**6** in thf-*d*_8_ (Table S1 and Figure S17).

Consequently, the formation of Sr^2+^-naphthyl interactions is preferential over bridging Sr–N bonds. That this structural change occurs for Sr^2+^ and Ba^2+^ but not for Ca^2+^ when R_3_ = Ph_3_ reflects the preference of the heavier congeners for the formation of multihaptic interactions with “soft” π-systems that are better able to saturate their large coordination spheres.^23^ In contrast, the subtle electronic modulation of the strength of the amide donors causes little variation between the structures of the calcium congeners **1** and **4** due to the stronger preference of Ca^2+^ for harder monodentate donors.^25^

This observation is further verified computationally with the orbital contribution of the dimerization interaction energy being approximately the same in **5** as in **2** (Figure S22). This suggests that strontium is the inflection point, where the Sr–N and Sr-arene interactions are balanced, whereas the calcium complexes favor “hard” interactions with the amido donors. In the barium complexes, the more diffuse orbital of the cations results in weaker Ba–N covalent interactions, and the structural driving force shifts to “soft” Ba-arene π interactions.

Similarly, the structure of barium congener **6** (Figure 3) shows less variance relative to the analogous [^Ph_2_Me^L]^2–^-supported complex **3**, as the latter already contains a Ba^2+^-naphthyl interaction in addition to its Ba_2_N_2_ ring. Consequently, the reduced charge density of the N-donor atoms in **6** that results from their SiPh_3_ substituents again results in full cleavage of the Ba(1)–N(2)* bond (which is already lengthened in **3**, *vide supra*) and conversion to a solely naphthalene-bridged structure.

Both the asymmetric unit and full dimeric structure of **6** vary little from those of the strontium congener **5**, notwithstanding the expected increases in Ba–N (Ba(1)–N(1) = 2.625(3) Å, Ba(1)–N(2) = 2.593(3) Å) and Ba-centroid_C(5)–C(10)_ (2.9129(12) Å) distances, as well as a consequently narrower N(1)–Ba(1)–N(2) bite angle (68.60(9)°), that result from the larger ionic radius of Ba^2+^. While the N(1)–N(2) distance in **6** is somewhat larger (2.941(4) Å) than that in **5**, inspection of the structure shows that this results from an in-plane widening of the metal-binding pocket rather than distortion of the highly planar naphthalene backbone (C(1–10)-plane_C(1)–C(10)_ = 0.001(1)–0.023(1) Å).

### NMR Spectroscopy in Aromatic Solvents

The insolubility of crystalline samples of **1**–**6** in benzene and toluene precluded the recording of informative NMR spectra at room temperature. **1**–**6** were also insoluble in 4:1 mixtures of 1,2-difluorobenzene and benzene-*d*_6_/toluene-*d*_8_, further demonstrating the insolubility of these aggregated species in noncoordinating solvents that do not disrupt their dimeric structures (in contrast with their facile dissolution in thf, *vide infra*). From *in situ* reactions at 25 °C, the rapid crystallization of **1**, **2**, **3**, **5**, and **6** resulted in spectra with broad, low-intensity ligand signals that were dominated by a sharp singlet at δ = 0.10 ppm corresponding to the byproduct HN (see the Supporting Information). However, [(^Ph_3_^L)Ca]_2_ (**4**) forms more slowly and remains solvated, allowing for solution-state characterization. The ^1^H NMR spectrum of **4** recorded *in situ* in C_6_D_6_ at 25 °C (Figure S9) consists of three naphthyl (δ = 7.05, 6.76, and 6.67 ppm) and three phenyl (δ = 7.55, 7.05, and 6.88 ppm) signals in the aromatic region, consistent with a *C*_2*v*_-symmetric structure, as well as the expected two equivalents of HN" per ligand. This contrasts with the asymmetrical dimeric solid-state structure (*vide infra*), suggesting that, in solution, **4** may have a fluxional structure that results in a time-averaged *C*_2*v*_-symmetric spectrum. This phenomenon has been reported for the NMR spectra of the dimeric precursors [AeN″_2_]_2_, in which the distinct terminal and bridging [*N*″]^−^ ligands observed in the solid state produce a single signal at room temperature as they exchange rapidly in solution due to the kinetic lability of Ae^2+^.^26^

### Generation of Monomeric thf Adducts

**Scheme 3:**
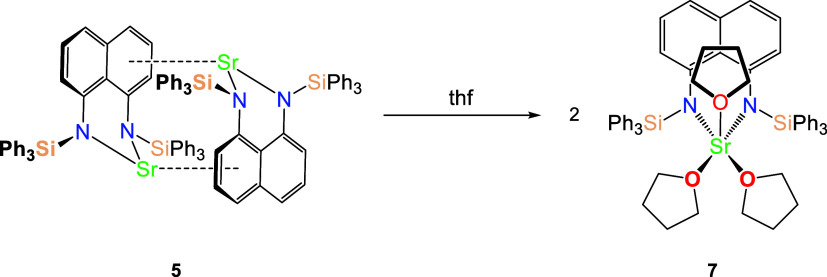
Synthesis of [(^R_3_^L)Ae(thf)_*n*_] from [(^R_3_^L)Ae]_2_ (**1**–**6**); Illustrated for [(^Ph_3_^L)Sr(thf)_3_] (**7**) Which was also Verified in the Solid State

Though poorly soluble in hydrocarbons, complexes **1**–**6** all dissolve readily in thf. This results in the disruption of their dimeric structures by the strong donor solvent and irreversible conversion into the monomeric thf adducts [(^R_3_^L)Ae(thf)_*n*_] (R_3_ = Ph_2_Me and Ph_3_, Ae = Ca, Sr and Ba) (Scheme 3).

Multinuclear NMR spectra (^1^H, ^13^C{^1^H} and ^29^Si) of **1**–**6**, recorded in thf-*d*_8_ were fully consistent with both their assignment as [(^R_3_^L)Ae(thf-*d*_8_)_*n*_] and with data reported for [(^Ph_2_Me^L)Mg(thf)_2_] by Kays and co-workers (**II**, Chart 1).^16^ The spectra of [^Ph_2_Me^L]^2–^-supported complexes closely resemble one another, as do the analogous spectra of the [^Ph_3_^L]^2–^-supported complexes. For example, for [(^Ph_3_^L)Sr(thf)_3_] (**7**), the ^1^H NMR spectrum consists of an aromatic region with three phenyl (δ = 7.70, 7.16, and 7.05 ppm) and three naphthyl (δ = 6.59, 6.56, and 6.52 ppm) resonances. Additionally, the ^13^C{^1^H} NMR spectrum consists of four phenyl and six naphthyl signals, while the ^29^Si NMR spectrum consists of a single signal at δ = **–**32.92 ppm. These data are fully consistent with both *C*_2*v*_ molecular symmetry and the solid-state structure of **7** (*vide infra*), with the [^R_3_^L]^2–^ ligand coordinated to Ae^2+^ in a κ^2^-*N*,*N*′-bidentate manner.

The irreversible conversion of **1**–**6** into monomeric thf adducts contrasts with the solution-phase behavior of the related dimeric alkaline earth complexes **I-Ae** (Chart 1).^11,12^ NMR spectra of **I-Ae** revealed that they exist in equilibrium with their monomeric analogues when dissolved in thf, with full conversion to the monomeric species achieved only upon the addition of a crown ether. This reflects the additional coordinative saturation provided by the bis(phenoxyimine) “NOON” ligand, which stabilizes the aggregated dimeric form in the presence of a donor solvent. Conversely, the π-facial interactions present in **1**–**6** are more readily outcompeted by the coordination of thf than the dative Ae–N interactions present in **I-Ae**, thus enabling the facile conversion of **1**–**6** into monomeric thf adducts.

The ^29^Si NMR spectra of **1**–**6** in thf-*d*_8_ each consist of a low-frequency singlet corresponding to the silicon atoms of their SiR_3_ substituents. This ^29^Si NMR spectroscopic signal acts as a simple probe for inferring the influence of both the coordinated Ae^2+^ cation and the substituents of the SiR_3_ group on the electronic properties of the [^R_3_^L]^2–^ ligand (Table S1 and Figure S17).

While not taking into account the anisotropy effects of the phenyl rings, two clear trends can be identified. First, the ^29^Si NMR spectroscopic signal of a given ligand shifts to a lower frequency as the size and electropositivity of the Ae^2+^ cation to which it is coordinated increases. This results in more ionic Ae–N bonds and the retention of more charge density on the anionic amide donors, with the adjacent silicon atoms more shielded as a consequence. Second, the ^29^Si NMR signal of the [^Ph_3_^L]^2–^ ligand consistently resonates at a lower frequency than the corresponding [^Ph_2_Me^L]^2–^ signal upon their coordination to the same Ae^2+^ center, with a near-uniform change in chemical shift observed across the three metals (Δδ(SiR_3_) = 3.59 (Ca), 3.53 (Sr) and 3.59 (Ba) ppm).

This demonstrates the additional electron-withdrawing effect that results from the presence of an extra phenyl substituent, which results in SiPh_3_ groups that withdraw more electron density from the electron-rich [1,8-C_10_H_6_N_2_]^2–^ π-system and consequently have more shielded silicon centers than their SiPh_2_Me analogues. Therefore, the ^29^Si NMR signals of [(^Ph_2_Me^L)Ca(thf-*d*_8_)_*n*_] and [(^Ph_3_^L)Ba(thf-*d*_8_)_*n*_] are the least and most shielded, respectively, due to the combination of these ligand and metal effects, with the δ(SiR_3_) values of the other phenyl-substituted [(^R_3_^L)Ae(thf-*d*_8_)_*n*_] complexes intermediate between these extreme cases. These conclusions are consistent with computational molecular partitioning methods. Both natural population analysis (NPA) and the quantum theory of atoms in molecules (QTAIM) show the net charge at Ae increasing in complexes with SiPh_3_ ligands (**4**–**6**) relative to SiPh_2_Me (**1**–**3**) resulting from the electron-withdrawing nature of the additional phenyl group.^27^ Generally, the net positive charge on Ae decreases down the group, and the magnitude of the retained negative charge on the ligand nitrogen also decreases (Table S7). However, factors in addition to the formal charge must also be considered. While orbital overlap and covalency decrease down the group, the distribution of charge over the ligand may also change. Reflecting the changing coordination mode, the negative charge is delocalized more over the naphthalene backbone, which barium interacts with more effectively than strontium and calcium.

**Figure 4:**
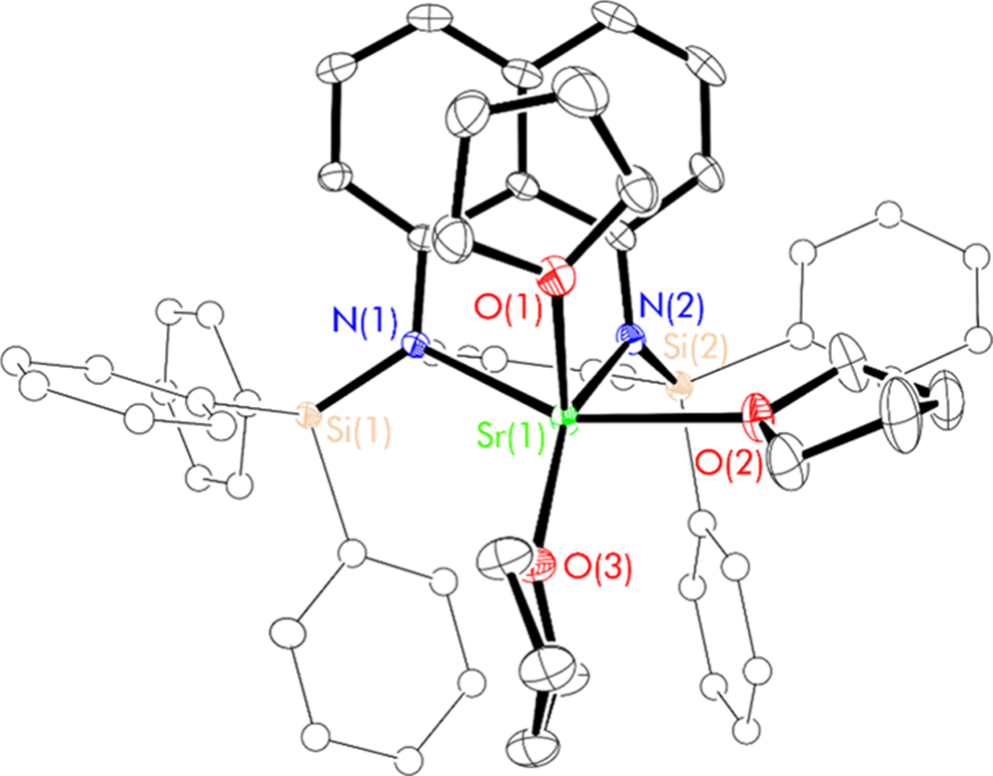
Thermal displacement ellipsoid drawing (30% probability) of [(^Ph_3_^L)Sr(thf)_3_] (**7**). All hydrogen atoms have been omitted, and phenyl groups are shown in ball-and-stick form for clarity.

Large block-like yellow single crystals of the monomeric thf adduct [(^Ph_3_^L)Sr(thf)_3_] (**7**) suitable for an X-ray diffraction study grew from a saturated thf solution of **5**. Complex **7** crystallizes in the *P*2_1_/*c* space group, with two crystallographically independent [(^Ph_3_^L)Sr(thf)_3_] molecules in the asymmetric unit. However, the metrical parameters of these two distinct molecules vary little; therefore, only one [(^Ph_3_^L)Sr(thf)_3_] complex (Figure 4 and Table S3) and selected metrical parameters from it are presented.

The strontium center in **7** is five-coordinate with a square pyramidal coordination geometry (τ_5_ = 0.075)^28^ and is bound to three thf molecules and to the [^Ph_3_^L]^2–^ ligand in a κ^2^-*N*,*N*′ manner. The apical position is occupied by a thf molecule that is projected above the naphthalene backbone, blocking any dimerization *via* the formation of a Sr^2+^-naphthyl π-facial interaction. The equatorial positions are occupied by the amide donors of the chelating [^Ph_3_^L]^2–^ ligand and two additional thf molecules that are necessarily *cis* to one another.

The Sr–N bond lengths in **7** are statistically indistinguishable from those in **5**. The [^Ph_3_^L]^2–^ ligand in **7** remains highly planar (C(1–10)-plane_C(1)–C(10)_ = 0.004(2)–0.071(1) Å), though with a slightly lengthened N(1)–N(2) distance (2.929(2) Å) relative to that of **5**. The Sr^2+^ cation lies 0.6554 Å further from the naphthalene plane in **7** than in **5** in order to accommodate the coordination of three thf molecules. Furthermore, the Sr^2+^-phenyl π-facial interactions that are present within the [(^Ph_3_^L)Sr] unit of **5** are not observed in **7**, having been outcompeted by the coordination of thf. The structure of **7** is similar to that of [(^Ph_2_Me^L)Mg(thf)_2_] (**II**, Chart 1),^16^ as in both cases, the [Ae(thf)_*n*_]^2+^ group lies out of the naphthalene plane on one face of a more planar [^R_3_^L]^2–^ ligand (Figure S20a,h).

It should be noted that while isolated crystalline samples of **1**–**6** were shown to be analytically pure by elemental analysis and are stable indefinitely when stored under an inert atmosphere, the monomeric adducts [(^R_3_^L)Ae(thf-*d*_8_)_*n*_] all undergo onward reactivity at 25 °C following their formation *in situ* in thf-*d*_8_. This is reminiscent of the solution-phase decomposition of a monomeric barium thf adduct supported by a chelating 1,3-di(amido)propane ligand reported by Jones and co-workers, though in this case, the lighter congeners proved stable in solution.^20^ Monitoring this decomposition process by ^1^H NMR spectroscopy in thf-*d*_8_ showed the formation of complex mixtures of products, including a high frequency (*N*,*N*)μ_2_-H resonance (*e.g.*, for **6**: δ = 14.49 ppm), which indicates the protonation of the dianionic [^Ph_3_^L]^2–^ ligand to form a monoanionic [^Ph_3_^LH]^−^ group with a strong intramolecular hydrogen bond, as well as the release of benzene (confirmed by a diagnostic singlet at δ = 7.16 ppm). This suggests that degradation results from the protonation of electron-rich phenyl substituents, although the origin of the abstracted proton is unclear. The reactivity of complexes of the form [(^R_3_^L)Ae(thf-*d*_8_)_*n*_] in solution is complex and is currently under investigation in greater detail using the reactivity of ^R_3_^LH_2_ with [(thf)_2_Ae*N*″_2_] as a model system.

## Conclusions

Full deprotonation of the phenyl-substituted proligands ^Ph_3_^LH_2_ and ^Ph_2_Me^LH_2_*via* protonolysis with [AeN″_2_]_2_ (Ae = Ca, Sr, and Ba) in benzene afforded the six new dimeric 1,8-bis(silylamido)naphthalene alkaline earth complexes [(^R_3_^L)Ae]_2_ (R_3_ = Ph_2_Me, Ae = Ca (**1**), Sr (**2**), and Ba (**3**); R_3_ = Ph_3_, Ae = Ca (**4**), Sr (**5**), and Ba (**6**)). NMR spectroscopic analysis of isolated samples of **1**–**6** in thf-*d*_8_ confirmed their conversion into the monomeric thf-*d*_8_ adducts [(^R_3_^L)Ae(thf-*d*_8_)_*n*_], with crystallographic verification provided by the X-ray crystal structure of [(^Ph_3_^L)Sr(thf)_3_] (**7**). A comparative analysis of the ^29^Si NMR spectra of **1**–**6** confirmed the influence of both the coordinated Ae^2+^ cation and the substituents of the SiR_3_ group on the electronic properties of the [^R_3_^L]^2–^ ligand.

X-ray crystallographic analysis showed that **1**, **2**, and **4** crystallize as nitrogen-bridged dimers, while **5** and **6** instead display a naphthalene-bridged motif, with **3** exhibiting a structure that is intermediate between the two distinct classes. The variance between these structures was rationalized based on the electron-withdrawing influence of the phenyl substituents of their ligands and the preferential formation of “soft” multihaptic π-facial interactions by the larger Sr^2+^ and Ba^2+^ cations. The structure and bonding of these complexes were additionally investigated with computational chemistry, which supported these conclusions. The Ae^2+^ cations in **1**–**6** increasingly sit out of the naphthalene backbone plane, with the 1,8-bis(silylamido)naphthalene ligand deviating less from planarity, as group 2 is descended.

Consequently, the flanking amide substituents provide a lessening degree of kinetic stabilization as the metal moves further from the in-plane binding pocket of the ligand, and so dimerization is necessary to saturate the coordination sphere of the otherwise exposed Ae^2+^ centers in all cases. The incorporation of phenyl substituents proved to be key to stabilizing the resulting dimeric species, facilitating both the formation of additional π-facial interactions with Ae^2+^ and the withdrawal of electron density from the highly basic amido donors. While the poor solubility and stability of **1**–**6** in arenes and thf, respectively, has hindered further investigation of their onward reactivity in solution, we are now investigating their conversion into reactive heterometallic species using mechanochemical methods.

## Experimental Section

All manipulations were carried out using standard Schlenk line or dry box techniques under an atmosphere of dinitrogen or argon. Protio solvents were degassed by sparging with dinitrogen, dried by passing through a column of activated sieves (pentane, hexane, toluene, benzene) and stored over potassium mirrors, or distilled from sodium metal (thf) and stored over activated 4 Å molecular sieves or distilled from sodium–potassium alloy (diethyl ether) and stored over a potassium mirror. Deuterated solvents were dried over potassium (C_6_D_6_, C_7_D_8_) or CaH_2_ (C_4_D_8_O), distilled under reduced pressure, and freeze**-**pump**-**thaw degassed 3 times prior to use.

^1^H NMR spectra were recorded at 298 K, unless otherwise stated, on Bruker AVIII 400 nanobays or Bruker AVIII 500 or Bruker NEO 600 spectrometers. ^13^C{^1^H} NMR spectra were recorded on the same spectrometers at operating frequencies of 100, 125, and 151 MHz, respectively, as were ^29^Si NMR spectra at operating frequencies of 80, 99, and 119 MHz, respectively. ^2^H NMR spectra were recorded on a Bruker AVIII 500 spectrometer at an operating frequency of 77 MHz. Two dimensional ^1^H–^1^H, ^13^C**–**^1^H, and ^29^Si–^1^H correlation experiments were used, when necessary, to confirm ^1^H, ^13^C, and ^29^Si assignments. NMR spectra were referenced internally to residual protio solvent (^1^H) or solvent (^13^C) resonances and are reported relative to tetramethylsilane (δ = 0 ppm). Chemical shifts are quoted in δ (ppm), and coupling constants are quoted in Hertz. Air-sensitive samples were prepared in a glovebox under an inert atmosphere, using dry deuterated solvents in 5 mm J-Young’s tap NMR tubes.

Samples for Fourier transform infrared (FTIR) spectroscopy were prepared in a glovebox under a dinitrogen atmosphere as pellets pressed with anhydrous potassium bromide, which was dried above 150 °C at 10^–6^ mbar for 24 h prior to use. FTIR spectra were measured using a Nicolet iS5 Thermo Scientific spectrometer, with a background spectrum run prior to the sample and subtracted from the sample spectrum.

Samples for elemental analysis were prepared in a glovebox under a nitrogen atmosphere and sealed in glass vials. CHN analyses were carried out in duplicate by Ms. Orla McCullough at London Metropolitan University.

^**Ph**_**3**_^**LH**_**2**_—procedure adapted from reference (13). 1,8-Diaminonaphthalene (4.00 g, 25.3 mmol) was dissolved in tetrahydrofuran (50 mL). The resulting deep purple solution was cooled to −30 °C, and then *n*-butyllithium (1.6 M in hexanes, 31.6 mL, 50.6 mmol, 2 equiv) was added dropwise over 20 minutes. The reaction mixture was allowed to warm to room temperature and stirred for 2 h. The resulting green suspension was cooled to −30 °C. A solution of triphenylsilyl chloride (14.9 g, 50.6 mmol, 2 equiv) in tetrahydrofuran (50 mL) was added dropwise over 20 minutes to form a dark brown suspension. The reaction mixture was allowed to warm to room temperature and stirred for 16 h. All solvents were removed, and the resulting yellow residue was dried *in vacuo* for 2 h. The residue was extracted with toluene (4 × 20 mL) and filtered through Celite. The resulting colorless solution was concentrated *in vacuo* to ∼10 mL. The addition of *n*-pentane (40 mL) and storage at −30 °C resulted in the precipitation of a white solid, which was isolated by filtration, washed with *n*-pentane (3 × 10 mL), and dried *in vacuo* for 1 h. ^Ph_3_^LH_2_ (4.57 g, 6.77 mmol, 27%) was isolated as a white powder. The toluene/*n*-pentane filtrate was left to stand at room temperature, resulting in the growth of large colorless crystals suitable for an X-ray diffraction study. ^1^H NMR (C_4_D_8_O, 298 K, 600 MHz): δ (ppm) = 7.53 (m, 12H, 2,6-C_6_***H***_***5***_), 7.31 (dt, ^3^*J*_H–H_ = 7.4, ^4^*J*_H–H_ = 1.4 Hz, 6H, 4-C_6_***H***_***5***_), 7.20 (m, 12H, 3,5-C_6_***H***_***5***_), 7.11 (dd, ^3^*J*_H–H_ = 8.2, ^4^*J*_H–H_ = 1.1 Hz, 2H, 4,5-C_10_***H***_***6***_), 6.86 (t, *J* = ^3^*J*_H–H_ = 7.8 Hz, 2H, 3,6-C_10_***H***_***6***_), 6.74 (dd, ^3^*J*_H–H_ = 7.5, ^4^*J*_H–H_ = 1.1 Hz, 2H, 2,7-C_10_***H***_***6***_), 6.67 (s, br, 2H, N***H***). ^13^C{^1^H} NMR (C_4_D_8_O, 298 K, 151 MHz): δ (ppm) = 144.58 (1,8-***C***_***10***_H_6_), 138.21 (10-***C***_***10***_H_6_), 136.67 (2,6-***C***_***6***_H_5_), 135.53 (1-***C***_***6***_H_5_), 130.61 (4-***C***_***6***_H_5_), 128.82 (3,5-***C***_***6***_H_5_), 126.06 (3,6-***C***_***10***_H_6_), 122.20 (9-***C***_***10***_H_6_), 121.10 (4,5-***C***_***10***_H_6_), 117.05 (2,7-***C***_***10***_H_6_).^29^Si NMR (C_4_D_8_O, 298 K, 119 MHz): δ (ppm) = −19.89 (***Si***Ph_3_). IR (KBr): 3312, 3269, 3065, 3056, 3021, 1576, 1427, 1412, 1290, 1111, 1027, 739, 714, 697, 516 cm^–1^. Anal. Calcd for C_46_H_38_N_2_Si_2_: C, 81.85; H, 5.67; N, 4.15. Found: C, 81.68; H, 5.83; N, 3.97.

### [(^Ph_2_Me^L)Ca]_2_ (**1**)

A solution of ^Ph_2_Me^LH_2_ (276 mg, 0.500 mmol, 2 equiv) in benzene (2 mL) was carefully layered over a solution of [CaN″_2_]_2_ (180 mg, 0.250 mmol, 1 equiv) in benzene (2 mL). The amber-colored reaction mixture was left to stand for 16 h, resulting in the growth of pale-yellow crystals suitable for an X-ray diffraction study. The solution was decanted, and the crystals were washed with benzene (3 × 0.5 mL) and *n*-pentane (3 × 1 mL) and then dried *in vacuo* for 1 h. [(^Ph_2_Me^L)Ca]_2_ (166 mg, 0.141 mmol, 56% (**1**)) was isolated as a pale-yellow crystalline solid. Yellow single crystals of **1** suitable for X-ray diffraction studies were grown by layering a benzene solution of ^Ph_2_Me^LH_2_ (2 equiv) over a benzene solution of [CaN″_2_]_2_ precursor (1 equiv). IR (KBr): 3065, 3047, 2952, 1578, 1542, 1427, 1268, 1110, 1055, 701 cm^–1^. Anal. Calcd for C_72_H_64_Ca_2_N_4_Si_4_: C, 73.42; H, 5.48; N, 4.76. Found: C, 73.21; H, 5.44; N, 4.34.

Dissolution of **1** in thf-*d*_8_ resulted in conversion to [(^Ph_2_Me^L)Ca(thf-*d*_8_)_*n*_], which could be characterized by multinuclear NMR spectroscopy. ^1^H NMR (C_4_D_8_O, 298 K, 600 MHz): δ (ppm) = 7.70 (dd, ^3^*J*_H–H_ = 7.4, ^4^*J*_H–H_ = 2.0 Hz, 8H, 2,6-C_6_***H***_***5***_), 7.18–7.11 (m, 12H, 3,5-C_6_***H***_***5***_ and 4-C_6_***H***_***5***_ overlapping), 6.59 (t, ^3^*J*_H–H_ = 7.5, 2H, 3,6-C_10_***H***_**6**_), 6.56 (dd, ^3^*J*_H–H_ = 7.8, ^4^*J*_H–H_ = 1.9 Hz, 2H, 4,5-C_10_***H***_***6***_), 6.45 (dd, ^3^*J*_H–H_ = 7.1, ^4^*J*_H–H_ 1.9 Hz, 2H, 2,7-C_10_***H***_***6***_), 0.61 (s, 6H, Si(C***H***_***3***_)Ph_2_). ^13^C{^1^H} NMR (C_4_D_8_O, 298 K, 151 MHz): δ (ppm) = 159.33 (1,8-***C***_***10***_H_6_), 145.17 (1-***C***_***6***_H_5_), 141.97 (9-***C***_***10***_H_6_), 136.25 (2,6-***C***_***6***_H_5_), 129.36 (10-***C***_***10***_H_6_), 128.45 (4-***C***_***6***_H_5_), 128.15 (3,5-***C***_***6***_H_5_), 125.69 (3,6-***C***_***10***_H_6_), 118.99 (2,7-***C***_***10***_H_6_), 115.19 (4,5-***C***_***10***_H_6_), 1.62 (Si(***C***H_3_)Ph_2_). ^29^Si NMR (C_4_D_8_O, 298 K, 119 MHz): δ (ppm) = −27.98 (N***Si***Ph_2_Me).

### [(^Ph_2_Me^L)Sr]_2_ (**2**)

A solution of ^Ph_2_Me^LH_2_ (551 mg, 1.00 mmol, 2 equiv) in benzene (2 mL) was carefully layered over a solution of [SrN″_2_]_2_ (409 mg, 0.500 mmol, 1 equiv) in benzene (3 mL). The amber-colored reaction mixture was left to stand for 16 h, resulting in the growth of bright yellow crystals suitable for an X-ray diffraction study. The solution was decanted, and the crystals were washed with benzene (3 × 1 mL) and *n*-pentane (3 × 2 mL) and then dried *in vacuo* for 1 h. [(^Ph_2_Me^L)Sr]_2_ (465 mg, 0.365 mmol, 73% (**2**)) was isolated as a bright yellow crystalline solid. Yellow single crystals of **2** suitable for X-ray diffraction studies were grown by layering a benzene solution of ^Ph_2_Me^LH_2_ (2 equiv) over a benzene solution of [Sr*N*″_2_]_2_ precursor (1 equiv). IR (KBr): 3044, 2953, 1541, 1424, 1366, 1105, 1054, 886, 701 cm^–1^. Anal. Calcd for C_72_H_64_N_4_Si_4_Sr_2_: C, 67.94; H, 5.07; N, 4.40. Found: C, 68.03; H, 5.16; N, 4.11.

Dissolution of **2** in thf-*d*_8_ resulted in conversion to [(^Ph_2_Me^L)Sr(thf-*d*_8_)_*n*_], which could be characterized by multinuclear NMR spectroscopy. ^1^H NMR (C_4_D_8_O, 298 K, 600 MHz): δ (ppm) = 7.75 (m, 8H, 2,6-C_6_***H***_***5***_), 7.16–7.06 (m, 12H, 3,5-C_6_***H***_***5***_ and 4-C_6_***H***_***5***_ overlapping), 6.57 (t, ^3^*J*_H–H_ = 7.6 Hz, 2H, 3,6-C_10_***H***_***6***_), 6.52 (dd, ^3^*J*_H–H_ = 7.9, ^4^*J*_H–H_ = 1.5 Hz, 2H, 4,5-C_10_***H***_***6***_), 6.44 (dd, ^3^*J*_H–H_ = 7.4, ^4^*J*_H–H_ = 1.5 Hz, 2H, 2,7-C_10_***H***_***6***_), 0.58 (s, 6H, Si(C***H***_***3***_)Ph_2_). ^13^C{^1^H} NMR (C_4_D_8_O, 298 K, 151 MHz): δ (ppm) = 159.98 (1,8-***C***_***10***_H_6_), 145.48 (1-***C***_***6***_H_5_), 141.99 (9-***C***_***10***_H_6_), 136.34 (2,6-***C***_***6***_H_5_), 130.28 (10*-***C**_***10***_H_6_), 128.30 (4-***C***_***6***_H_5_), 128.05 (3,5*-***C**_***6***_H_5_), 125.51 (3,6-***C***_***10***_H_6_), 119.09 (2,7-***C***_***10***_H_6_), 114.50 (4,5*-***C**_***10***_H_6_), 1.97 (Si(***C***H_3_)Ph_2_). ^29^Si NMR (C_4_D_8_O, 298 K, 119 MHz): δ (ppm) = −29.39 (N***Si***Ph_2_Me).

### [(^Ph_2_Me^L)Ba]_2_ (**3**)

A solution of ^Ph_2_Me^LH_2_ (551 mg, 1.00 mmol, 2 equiv) in benzene (2 mL) was carefully layered over a solution of [BaN″_2_]_2_ (459 mg, 0.500 mmol, 1 equiv) in benzene (3 mL). The amber-colored reaction mixture was left to stand for 16 h, resulting in the growth of bright yellow crystals suitable for an X-ray diffraction study. The solution was decanted, and the crystals were washed with benzene (3 × 1 mL) and *n*-pentane (3 × 2 mL) and then dried *in vacuo* for 1 h. [(^Ph_2_Me^L)Ba]_2_·(C_6_H_6_) (560 mg, 0.386 mmol, 77% (**3**)) was isolated as a bright yellow, crystalline solid. Yellow single crystals of **3** suitable for X-ray diffraction studies were grown by layering a benzene solution of ^Ph_2_Me^LH_2_ (2 equiv) over a benzene solution of [Ba*N*″_2_]_2_ (1 equiv). IR (KBr): 3038, 3012, 2952, 1532, 1424, 1365, 1319, 1106, 1068, 894, 736 cm^–1^. Anal. Calcd for C_72_H_64_Ba_2_N_4_Si_4_·(C_6_H_6_): C, 64.59; H, 4.86; N, 3.86. Found: C, 63.87; H, 4.55; N, 3.46.

Dissolution of **3** in thf-*d*_8_ resulted in conversion to [(^Ph_2_Me^L)Ba(thf-*d*_8_)_*n*_], which could be characterized by multinuclear NMR spectroscopy. ^1^H NMR (C_4_D_8_O, 298 K, 600 MHz): δ (ppm) = 7.72 (dd, ^3^*J*_H–H_ = 7.6, ^4^*J*_H–H_ = 1.7 Hz, 8H, 2,6-C_6_***H***_***5***_), 7.20–7.08 (m, 12H, 3,5-C_6_***H***_***5***_ and 4-C_6_***H***_***5***_ overlapping), 6.59 (t, ^3^*J*_H–H_ = 7.7 Hz, 2H, 3,6-C_10_***H***_***6***_), 6.52 (dd, ^3^*J*_H–H_ = 7.8, ^4^*J*_H–H_ = 1.4 Hz, 2H, 4,5-C_10_***H***_***6***_), 6.39 (dd, ^3^*J*_H–H_ = 6.8, ^4^*J*_H–H_ = 1.4 Hz, 2H, 2,7-C_10_***H***_***6***_), 0.62 (s, 6H, Si(C***H***_***3***_)Ph_2_). ^13^C{^1^H} NMR (C_4_D_8_O, 298 K, 151 MHz): δ (ppm) = 158.89 (1,8-***C***_***10***_H_6_), 145.34 (1-***C***_***6***_H_5_), 142.30 (9-***C***_***10***_H_6_), 136.34 (2,6-***C***_***6***_H_5_), 129.39 (10*-***C**_***10***_H_6_), 128.40 (4-***C***_***6***_H_5_), 128.17 (3,5*-***C**_***6***_H_5_), 125.72 (3,6-***C***_***10***_H_6_), 118.49 (2,7-***C***_***10***_H_6_), 114.14 (4,5*-***C**_***10***_H_6_), 1.57 (Si(***C***H_3_)Ph_2_). ^29^Si NMR (C_4_D_8_O, 298 K, 119 MHz): δ (ppm) = −30.64 (N***Si***Ph_2_Me).

### [(^Ph_3_^L)Ca]_2_ (**4**)

A solution of ^Ph_3_^LH_2_ (254 mg, 0.376 mmol, 2 equiv) in benzene (2 mL) was added to a solution of [CaN″_2_]_2_ (136 mg, 0.188 mmol, 1 equiv) in benzene (2 mL). The yellow reaction mixture was left to stand for 16 h and then lyophilized to yield a bright yellow powder. Benzene (10 mL) was added, and the mixture was refluxed at 100 °C to redissolve the yellow solid. Slow cooling of the solution to 25 °C resulted in the growth of bright yellow crystals suitable for an X-ray diffraction study. The solution was decanted, and the crystals were washed with benzene (0.5 mL) and *n*-pentane (3 × 0.5 mL) and then dried *in vacuo* for 1 h. [(^Ph_3_^L)Ca]_2_ (98 mg, 0.069 mmol, 37% (**4**)) was isolated as a yellow crystalline solid. IR (KBr): 3066, 3049, 2992, 1544, 1427, 1279, 1103, 881, 832, 703 cm^–1^. Anal. Calcd for C_92_H_72_Ca_2_N_4_Si_4_: C, 77.48; H, 5.09; N, 3.93. Found: C, 77.70; H, 5.20; N, 3.15.

Dissolution of **4** in thf-*d*_8_ resulted in conversion to [(^Ph_3_^L)Ca(thf-*d*_8_)_*n*_], which could be characterized by multinuclear NMR spectroscopy. ^1^H NMR (C_4_D_8_O, 298 K, 600 MHz): δ (ppm) = 7.70 (d, ^3^*J*_H–H_ = 7.3 Hz, 12H, 2,6-C_6_***H***_***5***_), 7.16 (t, ^3^*J*_H–H_ = 7.5 Hz, 6H, 4-C_6_***H***_***5***_), 7.06 (t, ^3^*J*_H–H_ = 7.4 Hz, 12H, 3,5-C_6_***H***_***5***_), 6.66–6.62 (m, 4H, 4,5-C_10_***H***_***6***_ and 2,7-C_10_***H***_***6***_ overlapping), 6.55 (t, ^3^*J*_H–H_ = 7.6 Hz, 2H, 3,6-C_10_***H***_***6***_).^13^C{^1^H} NMR (C_4_D_8_O, 298 K, 151 MHz): δ (ppm) = 158.56 (1,8-***C***_***10***_H_6_), 143.06 (1-***C***_***6***_H_5_), 141.73 (9-***C***_***10***_H_6_), 137.22 (2,6-***C***_***6***_H_5_), 129.53 (10-***C***_***10***_H_6_), 128.72 (4-***C***_***6***_H_5_), 128.26 (3,5-***C***_***6***_H_5_), 125.56 (3,6-***C***_***10***_H_6_), 120.51 (2,7-***C***_***10***_H_6_), 116.12 (4,5-***C***_***10-***_H_6_). ^29^Si NMR (C_4_D_8_O, 298 K, 119 MHz): δ (ppm) = −31.57 (N***Si***Ph_3_).

### [(^Ph_3_^L)Sr]_2_ (**5**)

A solution of ^Ph_3_^LH_2_ (254 mg, 0.375 mmol, 2 equiv) in benzene (2 mL) was carefully layered over a solution of [SrN″_2_]_2_ (172 mg, 0.188 mmol, 1 equiv) in benzene (2 mL). The amber-colored reaction mixture was left to stand for 16 h, resulting in the growth of bright yellow crystals suitable for an X-ray diffraction study. The solution was decanted, and the crystals were washed with benzene (3 × 0.5 mL) and *n*-pentane (3 × 1 mL) and then dried *in vacuo* for 1 h. [(^Ph_3_^L)Sr]_2_ (225 mg, 0.148 mmol, 79% (**5**)) was isolated as a bright yellow crystalline solid. IR (KBr): 3065, 3043, 1533, 1424, 1364, 1327, 1105, 830, 704, 516 cm^–1^. Anal. Calcd for C_92_H_72_N_4_Si_4_Sr_2_: C, 72.64; H, 4.77; N, 3.68. Found: C, 71.72; H, 4.24; N, 3.30.

Dissolution of **5** in thf-*d*_8_ resulted in conversion to [(^Ph_3_^L)Sr(thf-*d*_8_)_3_] (**7**), which could be characterized by multinuclear NMR spectroscopy and also in the solid state using single-crystal X-ray diffraction. ^1^H NMR (C_4_D_8_O, 298 K, 600 MHz): δ (ppm) = 7.70 (dd, ^3^*J*_H–H_ = 7.9, ^4^*J*_H–H_ = 1.5 Hz, 12H. 2,6-C_6_***H***_***5***_), 7.16 (ddt, ^3^*J*_H–H_ = 8.8, 6.9, ^4^*J*_H–H_ = 1.4 Hz, 6H, 4-C_6_***H***_***5***_), 7.05 (t, ^3^*J*_H–H_ = 7.4 Hz, 12H, 3,5-C_6_***H***_***5***_), 6.59 (dd, ^3^*J*_H–H_ = 7.6, ^4^*J*_H–H_ = 1.6 Hz, 2H, 4,5-C_10_***H***_***6***_), 6.56 (dd, ^3^*J*_H–H_ = 7.6, ^4^*J*_H–H_ = 1.6 Hz, 2H, 2,7-C_10_***H***_***6***_), 6.52 (t, ^3^*J*_H–H_ = 7.5 Hz, 2H, 3,6-C_10_***H***_***6***_). ^13^C{^1^H} NMR (C_4_D_8_O, 298 K, 151 MHz): δ (ppm) = 158.59 (1,8-***C***_***10***_H_6_), 143.50 (1-*C*_6_H_5_), 142.21 (9-***C***_***10***_H_6_), 137.15 (2,6-***C***_***6***_H_5_), 129.34 (10-***C***_***10***_H_6_), 128.66 (4-***C***_***6***_H_5_), 128.31 (3,5-***C***_***6***_H_5_), 125.60 (3,6-***C***_***10***_H_6_), 120.44 (2,7-***C***_***10***_H_6_), 115.46 (4,5-***C***_***10***_H_6_). ^29^Si NMR (C_4_D_8_O, 298 K, 119 MHz): δ (ppm) = −32.92 (N***Si***Ph_3_).

### [(^Ph_3_^L)Ba]_2_ (**6**)

A solution of ^Ph_3_^LH_2_ (254 mg, 0.375 mmol, 2 equiv) in benzene (2 mL) was carefully layered over a solution of [BaN″_2_]_2_ (172 mg, 0.188 mmol, 1 equiv) in benzene (3 mL). The amber-colored reaction mixture was left to stand for 16 h, resulting in the growth of bright yellow crystals suitable for an X-ray diffraction study. The solution was decanted, and the crystals were washed with benzene (3 × 0.5 mL) and *n*-pentane (3 × 1 mL) and then dried *in vacuo* for 1 h. [(^Ph_3_^L)Ba]_2_ (244 mg, 0.151 mmol, 80% (**6**)) was isolated as a bright yellow crystalline solid. IR (KBr): 3065, 3042, 1531, 1426, 1325, 1105, 900, 703, 514 cm^–1^. Anal. Calcd for C_92_H_72_Ba_2_N_4_Si_4_: C, 68.18; H, 4.48; N, 3.46. Found: C, 67.75; H, 4.32; N, 3.22.

Dissolution of **6** in thf-*d*_8_ resulted in conversion to [(^Ph_3_^L)Ba(thf-*d*_8_)_*n*_], which could be characterized by multinuclear NMR spectroscopy. ^1^H NMR (C_4_D_8_O, 298 K, 600 MHz): δ (ppm) = 7.69 (d, ^3^*J*_H–H_ = 7.2 Hz, 12H, 2,6-C_6_***H***_***5***_), 7.16 (t, ^3^*J*_H–H_ = 7.4 Hz, 6H, 4-C_6_***H***_***5***_), 7.06 (t, ^3^*J*_H–H_ = 7.4 Hz, 12H, 3,5-C_6_***H***_***5***_), 6.58 (dd, ^3^*J*_H–H_ = 7.1, ^4^*J*_H–H_ = 2.2 Hz, 2H, 4,5-C_10_***H***_***6***_), 6.54 (m, 4H, 3,6-C_10_***H***_***6***_ and 2,7-C_10_***H***_***6***_ overlapping). ^13^C{^1^H} NMR (C_4_D_8_O, 298 K, 151 MHz): δ (ppm) = 157.75 (1,8-***C***_***10***_H_6_), 143.27 (1-***C***_***6***_H_5_), 142.33 (9-***C***_***10***_H_6_), 137.24 (2,6-***C***_***6***_H_5_), 129.18 (10-***C***_***10***_H_6_), 128.68 (4-***C***_***6***_H_5_), 128.27 (3,5-***C***_***6***_H_5_), 125.74 (3,6-***C***_***10***_H_6_), 120.00 (2,7-***C***_***10***_H_6_), 114.87 (4,5-***C***_***10***_H_6_). ^29^Si NMR (C_4_D_8_O, 298 K, 119 MHz): δ (ppm) = −34.23 (N***Si***Ph_3_).

### [(^Ph_3_^L)Sr(thf)_3_] (**7**)

Dissolution of [(^Ph_3_^L)Sr]_2_ (**5**; 30 mg, 20 μmol) in thf (0.5 mL) resulted in the growth of large block-like yellow crystals of [(^Ph_3_^L)Sr(thf)_3_] (**7**) suitable for an X-ray diffraction study when left to stand at 25 °C for 24 h. Complex **7** was not isolated on a preparative scale.

For complexes **3**, **4**, and **5**, elemental analysis indicates a greater difference than ±0.4% between the calculated and measured carbon (**3** and **5**) or nitrogen (**4**) values. All analyses were carried out on spectroscopically pure, recrystallized samples at least twice. This is a phenomenon in alkaline earth chemistry for which there is precedent.^29^

In addition, we note that the crystal structures of 3 and 4 contain 1 and 1.75 equiv of cocrystallized benzene per dimer, respectively. It is unclear to what extent this is retained after drying in vacuo as part of sample preparation—NMR spectroscopy cannot determine this accurately due to additional benzene produced during the decomposition of [(^R_3_^L)Ae(thf-*d*_8_)*_n_*] in solution. This may also lead to a discrepancy in the elemental analysis value.

## Supporting Information Available

General experimental details; representative NMR spectra; additional crystallographic data and computational chemistry data (PDF)
Cartesian coordinates of computationally optimized geometries (XYZ)

## Supplementary Material

om4c00479_si_001.pdf

om4c00479_si_002.xyz

## References

[ref1] aBhoelan B. S.; Stevering C. H.; van der Boog A. T.; van der Heyden M. A. Barium toxicity and the role of the potassium inward rectifier current. Clin. Toxicol. 2014, 52 (6), 584–593. 10.3109/15563650.2014.923903. 24905573

[ref2] aChapple P. M.; Sarazin Y. Contemporary Molecular Barium Chemistry. Eur. J. Inorg. Chem. 2020, 2020 (35), 3321–3346. 10.1002/ejic.202000501.

[ref3] aMoxey G. J.; Blake A. J.; Lewis W.; Kays D. L. Alkaline Earth Complexes of a Sterically Demanding Guanidinate Ligand. Eur. J. Inorg. Chem. 2015, 2015 (36), 5892–5902. 10.1002/ejic.201501239.

[ref4] aGao J.; Liu Y.; Zhao Y.; Yang X.-J.; Sui Y. Syntheses and Structures of Magnesium Complexes with Reduced α-Diimine Ligands. Organometallics 2011, 30 (22), 6071–6077. 10.1021/om2003199.

[ref5] Freitag B.; Fischer C. A.; Penafiel J.; Ballmann G.; Elsen H.; Färber C.; Piesik D. F.; Harder S. Bora-amidinate as a cooperative ligand in group 2 metal catalysis. Dalton Trans. 2017, 46 (34), 11192–11200. 10.1039/C7DT02136D. 28745370

[ref6] aKottalanka R. K.; Adimulam H.; Bhattacharjee J.; Babu H. V.; Panda T. K. Bis(phosphinoselenoic amides) as versatile chelating ligands for alkaline earth metal (Mg, Ca, Sr and Ba) complexes: syntheses, structure and ε-caprolactone polymerisation. Dalton Trans. 2014, 43 (23), 8757–8766. 10.1039/C4DT00669K. 24777284

[ref7] aLiu H.-Y.; Schwamm R. J.; Neale S. E.; Hill M. S.; McMullin C. L.; Mahon M. F. Reductive Dimerization of CO by a Na/Mg(I) Diamide. J. Am. Chem. Soc. 2021, 143 (42), 17851–17856. 10.1021/jacs.1c09467. 34652134 PMC8554760

[ref8] O’Reilly A.; Haynes M. D.; Turner Z. R.; McMullin C. L.; Harder S.; O’Hare D.; Fulton J. R.; Coles M. P. Mixing and matching N,N- and N,O-chelates in anionic Mg(i) compounds: synthesis and reactivity with RN–C–NR and CO. Chem. Commun. 2024, 60 (56), 7204–7207. 10.1039/D4CC02594F. 38910507

[ref9] McMullen J. S.; Edwards A. J.; Hicks J. C–H and C–F coordination of arenes in neutral alkaline earth metal complexes. Dalton Trans. 2021, 50 (25), 8685–8689. 10.1039/D1DT01532J. 34160514

[ref10] aMcMullen J. S.; Huo R.; Vasko P.; Edwards A. J.; Hicks J. Anionic Magnesium and Calcium Hydrides: Transforming CO into Unsaturated Disilyl Ethers. Angew. Chem., Int. Ed. 2023, 62 (1), e202215218 10.1002/anie.202215218. PMC10100151 36344462

[ref11] Jones R. L.; Turner Z. R.; Buffet J.-C.; O’Hare D. Bis(phenoxy-imine) Alkaline–Earth Complexes with Competing Binding Pockets. Organometallics 2024, 43 (3), 414–426. 10.1021/acs.organomet.3c00504.

[ref12] Jones R. L.; Turner Z. R.; Buffet J.-C.; O’Hare D. Phenoxy imine NOON complexes of heavy alkaline earth ions for the ring-opening polymerisation of cyclic esters. Polym. Chem. 2024, 15 (17), 1767–1776. 10.1039/D4PY00103F.

[ref13] Gade L. H.; Galka C. H.; Hellmann K. W.; Williams R. M.; De Cola L.; Scowen I. J.; McPartlin M. Tetraaminoperylenes: Their Efficient Synthesis and Physical Properties. Chem. - Eur. J. 2002, 8 (16), 3732–3746. 10.1002/1521-3765(20020816)8:163.0.co;2-5. 12203300

[ref14] aBlake A. J.; Gillibrand N. L.; Moxey G. J.; Kays D. L. Differing Coordination Environments in Transition Metal Derivatives of 1,8-Bis(silylamido)naphthalene Ligands. Inorg. Chem. 2009, 48 (22), 10837–10844. 10.1021/ic901745v. 19824653

[ref15] aHellmann K. W.; Galka C.; Gade L. H.; Kottke T.; Stalke D. Aggregation of lithium and mixed thallium(I)–lithium amides through η^3^- and η^6^-π-arene interactions in the solid. Chem. Commun. 1998, 5, 549–550. 10.1039/a708139a.

[ref16] Bradley M. A.; Birchall C.; Blake A. J.; Lewis W.; Moxey G. J.; Kays D. L. 1,8-Bis(silylamido)naphthalene complexes of magnesium and zinc synthesised through alkane elimination reactions. Dalton Trans. 2017, 46 (12), 4101–4110. 10.1039/C7DT00471K. 28276554

[ref17] aLiu Q.; Wang C.; Zhang X.; Xue M.; Yao Y.; Zhang Y.; Shen Q. Synthesis and molecular structures of divalent bridged bis(guanidinate) europium complexes and their application in intermolecular hydrophosphination of alkenes and alkynes. New J. Chem. 2016, 40 (12), 10447–10454. 10.1039/C6NJ02508K.

[ref18] aNguyen D. T.; Helling C.; Evans M. J.; Jones C. Enforcing Metal–Arene Interactions in Bulky p-Terphenyl Bis(anilide) Complexes of Group 2 Metals (Be–Ba): Potential Precursors for Low-Oxidation-State Alkaline Earth Metal Systems. Inorg. Chem. 2024, 63 (12), 5718–5726. 10.1021/acs.inorgchem.4c00207. 38471088

[ref19] aThum K.; Martin J.; Elsen H.; Eyselein J.; Stiegler L.; Langer J.; Harder S. Lewis Acidic Cationic Strontium and Barium Complexes. Eur. J. Inorg. Chem. 2021, 2021 (26), 2643–2653. 10.1002/ejic.202100345.

[ref20] Nguyen D. T.; Helling C.; Jones C. Synthesis and Characterization of Bulky 1,3-Diamidopropane Complexes of Group 2 Metals (Be-Sr). Chem. - Asian J. 2024, 19 (15), e202400498 10.1002/asia.202400498. 38760323

[ref21] Mitoraj M.; Michalak A. Donor–Acceptor Properties of Ligands from the Natural Orbitals for Chemical Valence. Organometallics 2007, 26 (26), 6576–6580. 10.1021/om700754n.

[ref22] Lu T.; Chen Q. Independent gradient model based on Hirshfeld partition: A new method for visual study of interactions in chemical systems. J. Comput. Chem. 2022, 43 (8), 539–555. 10.1002/jcc.26812. 35108407

[ref23] Westerhausen M.; Schwarz W. Molekül- und Kristallstrukturen des dimeren Strontium-bis[bis(trimethylsilyl)amids] und des Strontium-bis[bis(trimethylsilyl)amids] · 2DME. Z. Anorg. Allg. Chem. 1991, 606 (1), 177–190. 10.1002/zaac.19916060118.

[ref24] Pyykkö P. Additive Covalent Radii for Single-, Double-, and Triple-Bonded Molecules and Tetrahedrally Bonded Crystals: A Summary. J. Phys. Chem. A 2015, 119 (11), 2326–2337. 10.1021/jp5065819. 25162610

[ref25] Harder S. From Limestone to Catalysis: Application of Calcium Compounds as Homogeneous Catalysts. Chem. Rev. 2010, 110 (7), 3852–3876. 10.1021/cr9003659. 20420358

[ref26] Westerhausen M. Synthesis and spectroscopic properties of bis(trimethylsilyl)amides of the alkaline-earth metals magnesium, calcium, strontium, and barium. Inorg. Chem. 1991, 30 (1), 96–101. 10.1021/ic00001a018.

[ref27] Bader R. F. W. A quantum theory of molecular structure and its applications. Chem. Rev. 1991, 91 (5), 893–928. 10.1021/cr00005a013.

[ref28] Addison A. W.; Rao T. N.; Reedijk J.; van Rijn J.; Verschoor G. C. Synthesis, structure, and spectroscopic properties of copper(II) compounds containing nitrogen–sulphur donor ligands; the crystal and molecular structure of aqua[1,7-bis(N-methylbenzimidazol-2′-yl)-2,6-dithiaheptane]copper(II) perchlorate. J. Chem. Soc., Dalton Trans. 1984, (7), 1349–1356. 10.1039/DT9840001349.

[ref29] aTorvisco A.; Ruhlandt-Senge K. Ligand and Coligand Effects on Ion Association in Magnesium Amides. Organometallics 2011, 30 (5), 986–991. 10.1021/om101045b.

