# Supplementary material for: Promoting π-Facial Interactions in Phenyl-Substituted 1,8-Bis(silylamido)naphthalene Alkaline Earth Complexes
Source: Organometallics. 2025 Feb 5;44(4):582–94. doi: 10.1021/acs.organomet.4c00479 (PMC11863540; doi:10.1021/acs.organomet.4c00479)
Supplement: Supplementary file 1 — om4c00479_si_001.pdf [file om4c00479_si_001.pdf]

Supporting Information

Promoting π-facial interactions in phenyl-substituted 1,8-bis(silylamido)naphthalene alkaline earth complexes.

Matthew D. Haynes, Clement G. Collins Rice, Louis J. Morris, Zoë R. Turner, and Dermot O'Hare*;*
e-mail: zoe.turner@earth.ox.ac.uk and *dermot.ohare@chem.ox.ac.uk*

Table of Contents

I.	General considerations	S2
II.	Representative NMR spectra	S3
III.	Crystallographic data	S15
IV.	Computational Chemistry	S26
V.	References	S34

I. **General considerations**

1,8 diaminonaphthalene (Sigma Aldrich), n¬ butyllithium (1.6 M or 2.5 M solution in hexanes; Sigma Aldrich), diphenylmethylsilylchloride (Sigma Aldrich), triphenylsilylchloride (Sigma Aldrich), magnesium bis(hexamethyldisilazide) (Sigma Aldrich), anhydrous calcium iodide (Sigma Aldrich), anhydrous strontium iodide (Sigma Aldrich), anhydrous barium iodide (Sigma Aldrich) and di-*n*-butyl magnesium (1.0 M in heptane, Sigma Aldrich) were used as received. Hexamethyldisilazane (Sigma Aldrich) and methanol-d_1 (Sigma Aldrich) was dried over calcium hydride, freeze pump thaw degassed three times and stored over pre activated 3 Å molecular sieves prior to use. Potassium hydride (Sigma Aldrich) was purchased as a 30 wt% dispersion in mineral oil. Excess oil was decanted away before the solid was washed thoroughly with diethyl ether until a free-flowing powder was obtained, which was dried in vacuo prior to use. Celite (Sigma Aldrich) was dried at 150 °C under reduced pressure (10^{-3} mbar) for at least 48 hours before use. Molecular sieves (Sigma Aldrich) were dried in vacuo at 140 °C for 48 hours prior to use.

1,8-{(R_3Si)NH}$_2C_{10}H_6$ ($^{R_3}LH_2$, R_3 = Ph_2Me),[1] KN($SiMe_3$)$_2$,[2] [(thf)$_2$Ae{N($SiMe_3$)$_2$}$_2$] (Ae = Ca, Sr and Ba),[3] and[Ae{N($SiMe_3$)$_2$}$_2$]$_2$ (Ae = Ca, Sr and Ba)[4] were prepared according to literature procedures.

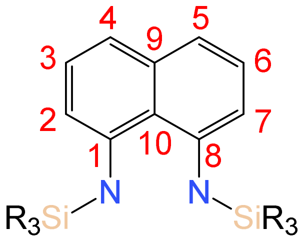

Naphthalene numbering scheme used in this publication:

II. Representative NMR spectra

$^{Ph3}LH_2$. In the ^{1}H NMR spectrum, the aromatic region consists of three naphthyl signals (δ = 7.11, 6.86 and 6.74 ppm) which resonate at lower frequencies than three phenyl signals (δ = 7.53, 7.31 and 7.20 ppm). In addition, a singlet integrating to two at δ = 6.67 ppm corresponds to the two amino protons. This spectrum reflects the C_{2v}-symmetric structure of $^{R3}LH_2$ in solution, with both amino protons sharing the same chemical environment. This symmetry is confirmed by the presence of six naphthyl resonances in the $^{13}C\{^{1}H\}$ spectrum of $^{Ph3}LH_2$ and one signal at δ = 19.89 ppm in its ^{29}Si NMR spectrum.

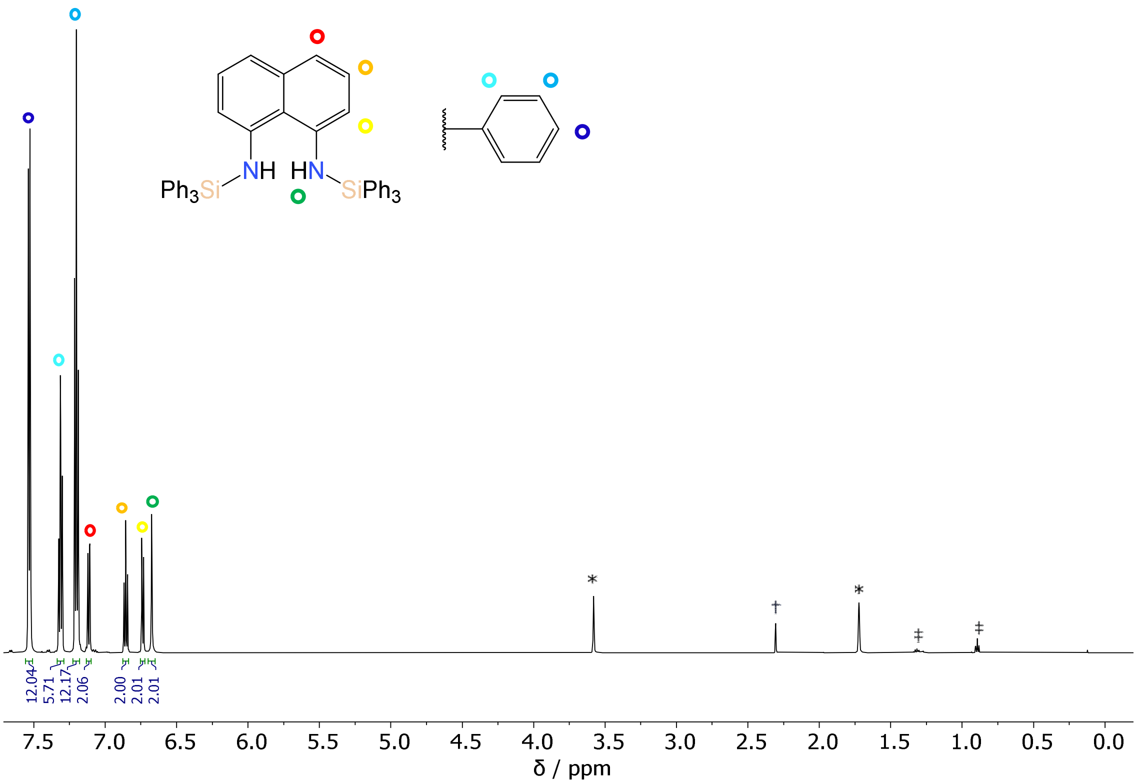

Figure S1. ^{1}H NMR spectrum (C_4D_8O, 298 K, 600.4 MHz) of $^{Ph3}LH_2$. ‡ denotes *n*-pentane, †, denotes toluene, * denotes residual protio fraction of C_4D_8O.

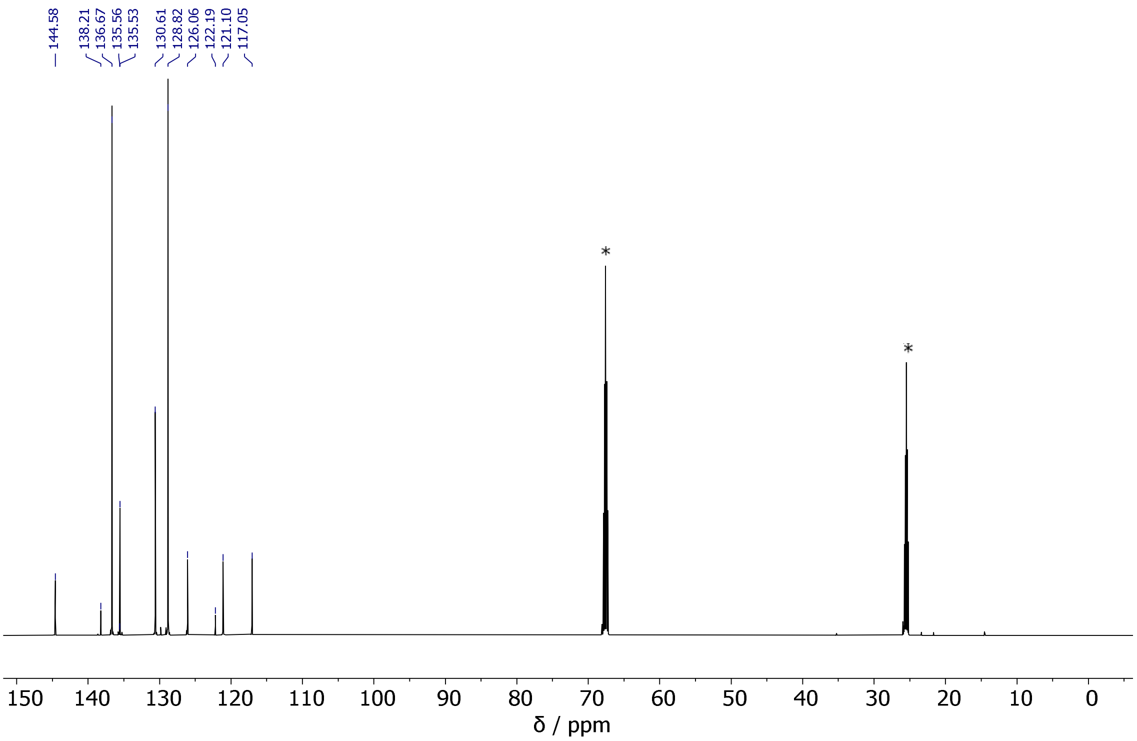

Figure S2. $^{13}C\{^{1}H\}$ NMR spectrum (C_4D_8O, 298 K, 151.0 MHz) of $^{Ph3}LH_2$. * denotes C_4D_8O.

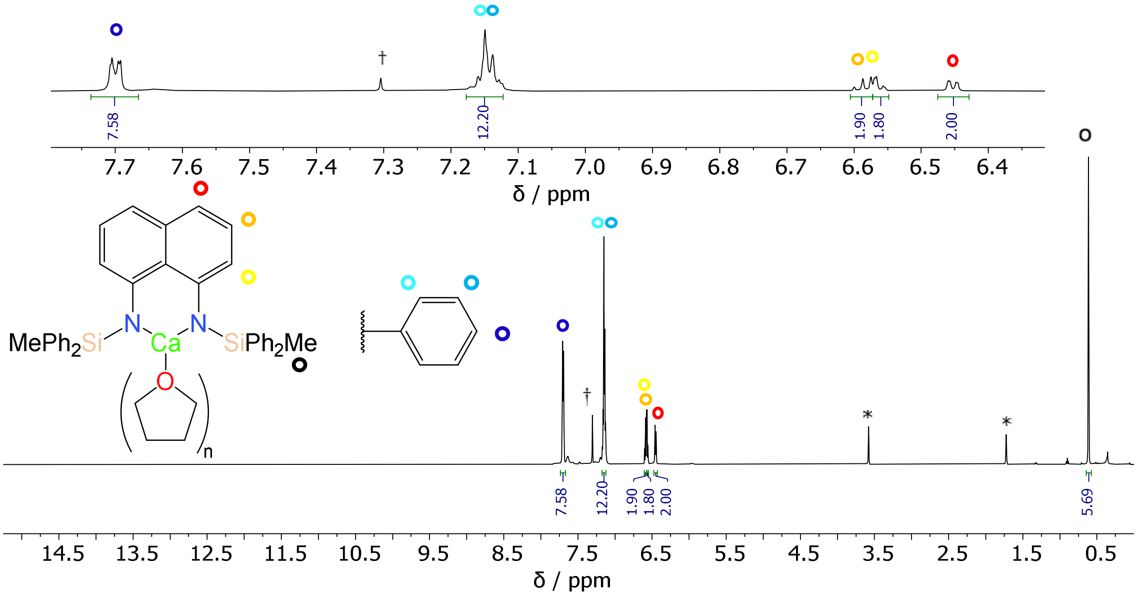

Figure S3. ^{1}H NMR spectrum (C_4D_8O, 298 K, 600.4 MHz) of $[(^{Ph_2Me}L)Ca(thf)_n]$, formed when $[(^{Ph_2Me}L)Ca]_2$ (**1**) is dissolved in thf. † denotes co-crystallised benzene, * residual protio fraction of denotes C_4D_8O.

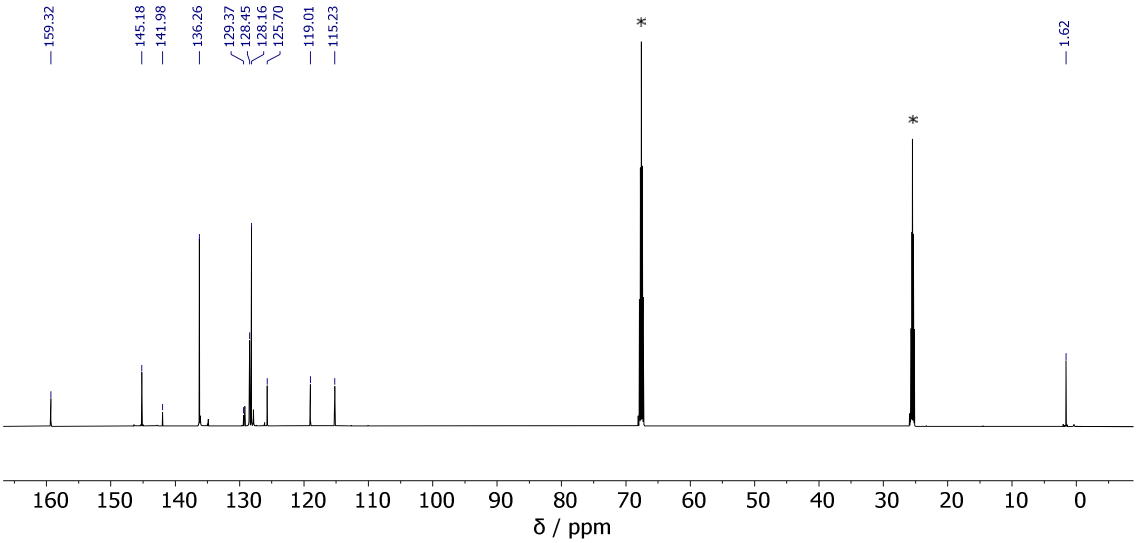

Figure S4. $^{13}C\{^{1}H\}$ NMR spectrum (C_4D_8O, 298 K, 151.0 MHz) of $[(^{Ph_2Me}L)Ca(thf)_n]$, formed when $[(^{Ph_2Me}L)Ca]_2$ (**1**) is dissolved in thf. * denotes C_4D_8O.

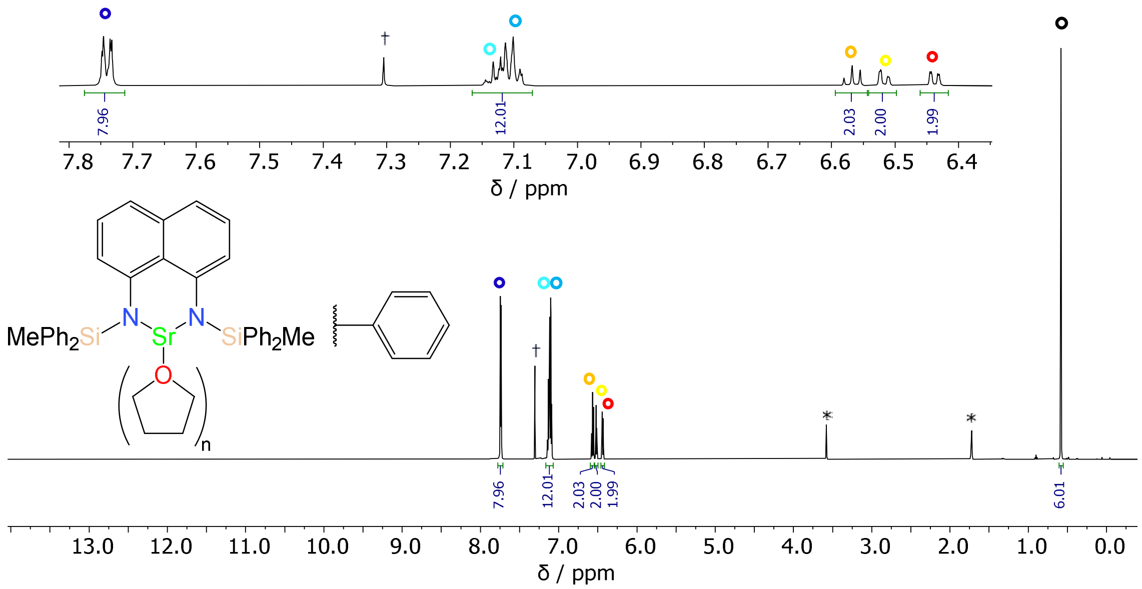

Figure S5. ^{1}H NMR spectrum (C_4D_8O, 298 K, 600.4 MHz) of [(^{Ph_2Me}L)Sr(thf)$_n$], formed when [(^{Ph_2Me}L)Sr]$_2$ (**2**) is dissolved in thf. † denotes co-crystallised benzene, * denotes residual protio fraction of C_4D_8O. Expansion shows aromatic region.

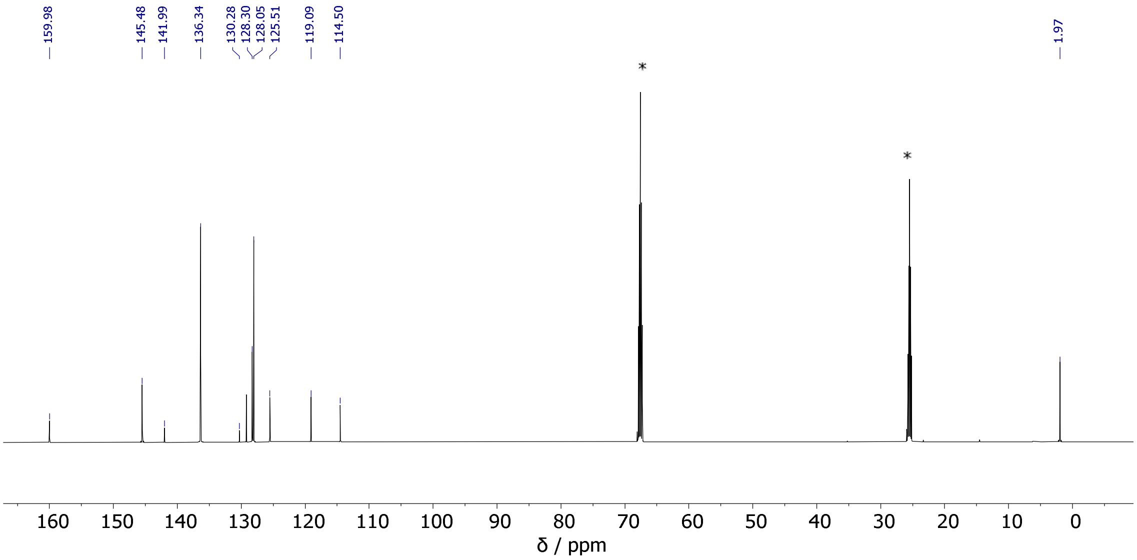

Figure S6. ^{1}H NMR spectrum (C_4D_8O, 298 K, 600.4 MHz) of [(^{Ph_2Me}L)Sr(thf)$_n$], formed when [(^{Ph_2Me}L)Sr]$_2$ (**2**) is dissolved in THF. † denotes co-crystallised benzene, * denotes residual protio fraction of C_4D_8O. Expansion shows aromatic region.

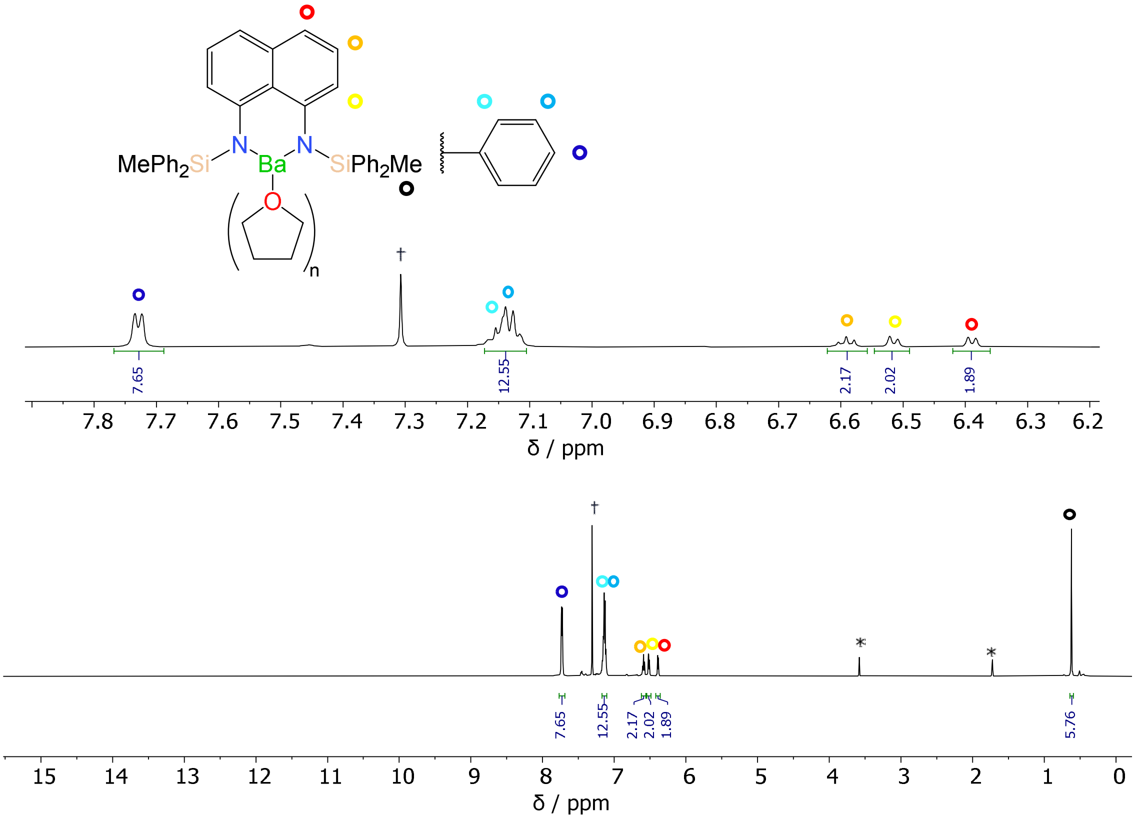

Figure S7. ^{1}H NMR spectrum (C_4D_8O, 298 K, 600.4 MHz) of [(^{Ph_2Me}L)Ba(thf)$_n$], formed when [(^{Ph_2Me}L)Ba]$_2$ (**3**) is dissolved in thf. † denotes co-crystallised benzene, * denotes residual protio fraction of C_4D_8O. Expansion shows aromatic region.

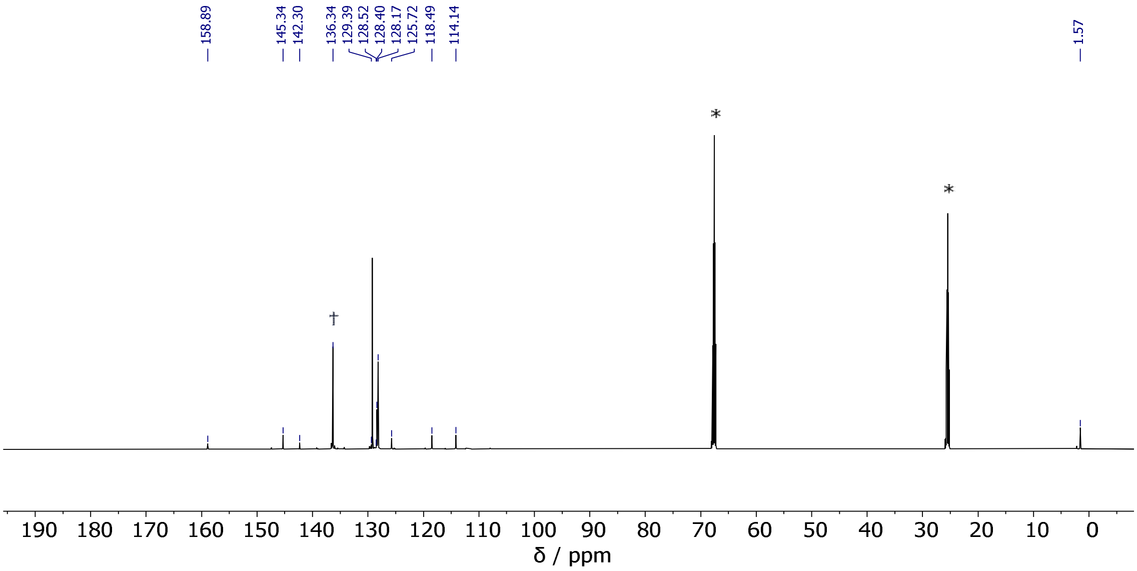

Figure S8. $^{13}C\{^{1}H\}$ NMR spectrum (C_4D_8O, 298 K, 151.0 MHz) of [(^{Ph_2Me}L)Ba(thf)$_n$], formed when [(^{Ph_2Me}L)Ba]$_2$ (**3**) is dissolved in thf. † denotes co-crystallised benzene, * denotes C_4D_8O.

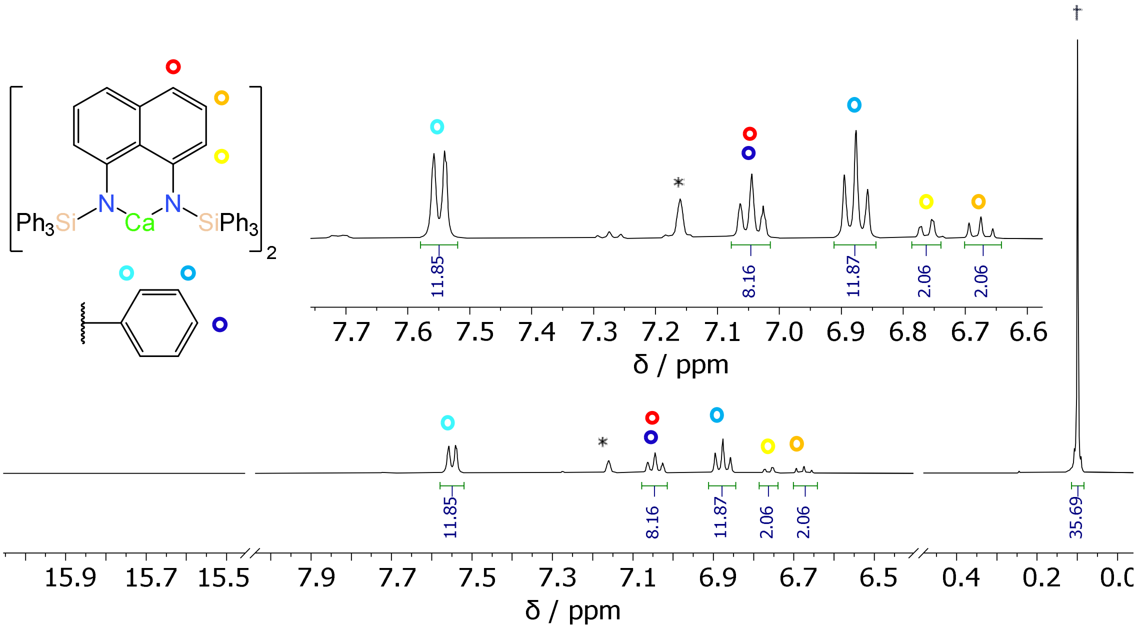

Figure S9. ^{1}H NMR spectrum (C_6D_6, 298 K, 400 MHz) of $[(^{Ph3}L)Ca]_2$ (**4**) prepared *in situ via* the 2:1 reaction of $^{Ph3}LH_2$ with $[CaN''_2]_2$ (spectrum taken after 16 h at 25 °C). Expansion shows the aromatic region. † denotes HN'', * denotes residual protio fraction of C_6D_6.

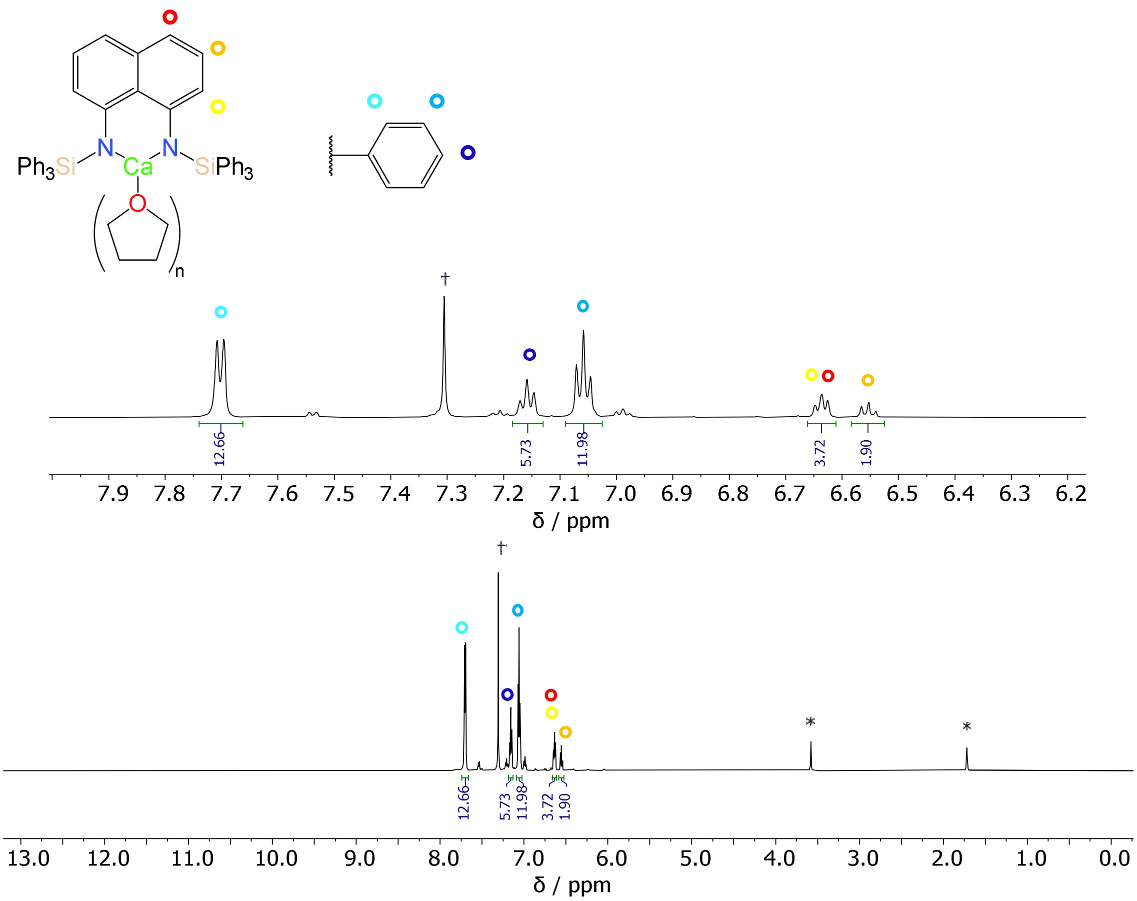

Figure S10. ^{1}H NMR spectrum (C_4D_8O, 298 K, 600.4 MHz) of [(^{Ph3}L)Ca(thf)$_n$], formed when [(^{Ph3}L)Ca]$_2$ (**4**) is dissolved in thf. † denotes co-crystallised benzene, * denotes residual protio fraction of C_4D_8O. Expansion shows aromatic region.

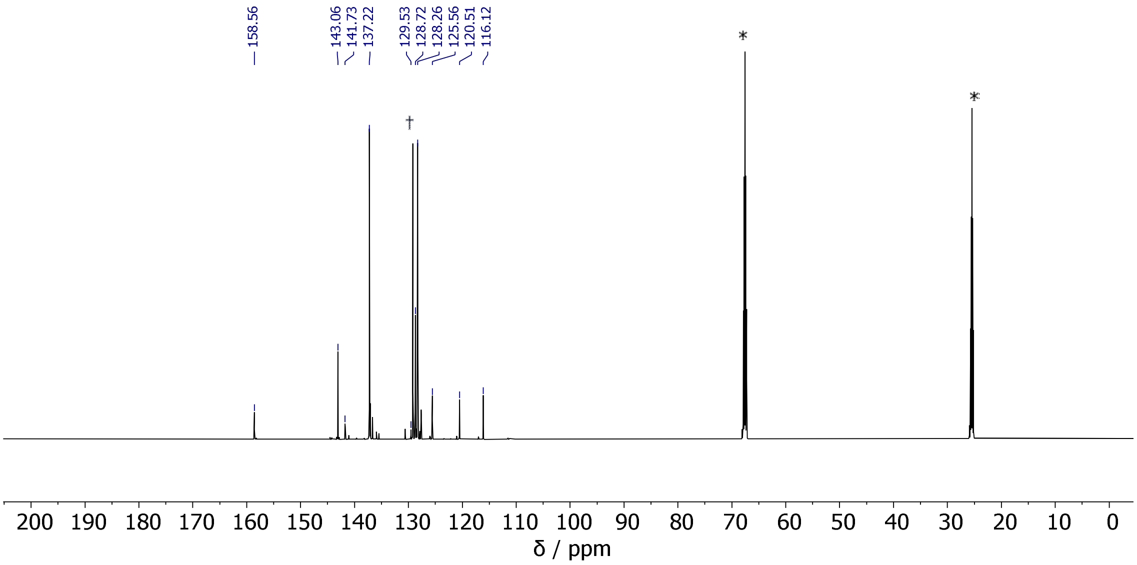

Figure S11. $^{13}C\{^{1}H\}$ NMR spectrum (C_4D_8O, 298 K, 151.0 MHz) of [(^{Ph3}L)Ca(thf)$_n$], formed when [(^{Ph3}L)Ca]$_2$ (**4**) is dissolved in thf. † denotes co-crystallised benzene, * denotes C_4D_8O.

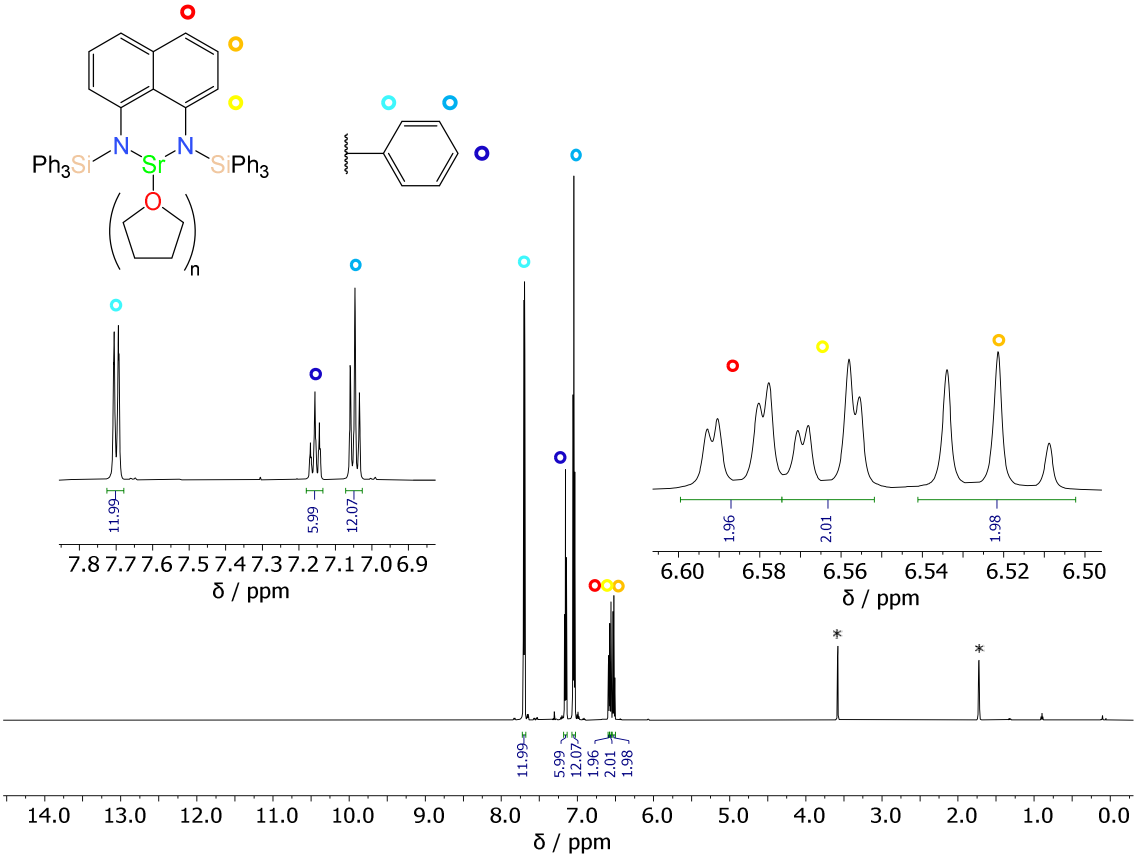

Figure S12. ^{1}H NMR spectrum (C_4D_8O, 298 K, 600.4 MHz) of [(^{Ph3}L)Sr(thf)$_3$] (**7**), formed when [(^{Ph3}L)Sr]$_2$ (**5**) is dissolved in thf. * denotes residual protio fraction of C_4D_8O. Expansions show phenyl and naphthyl regions.

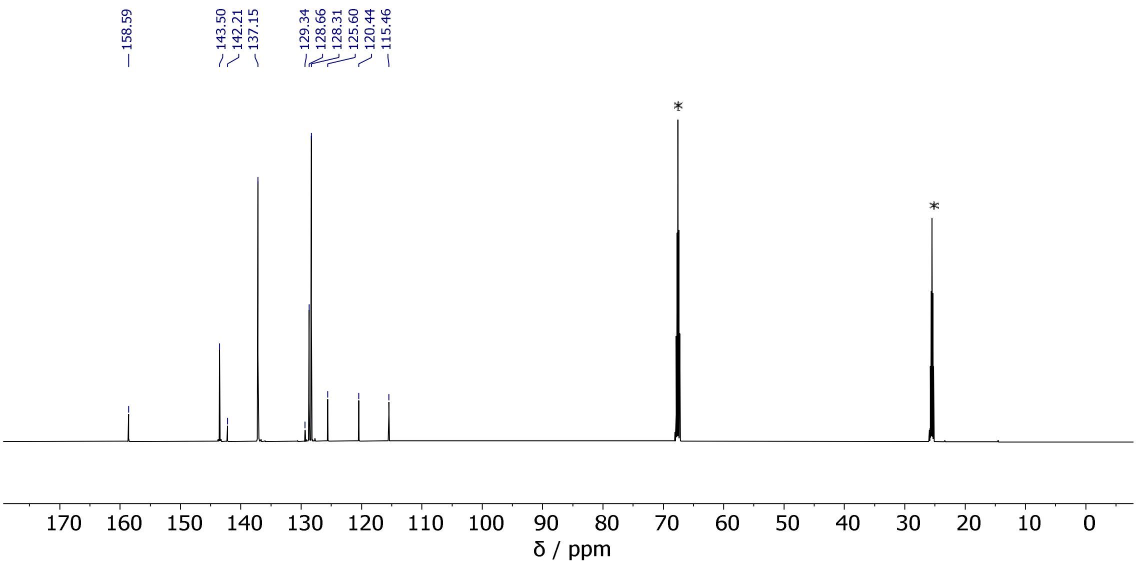

Figure S13. $^{13}C\{^{1}H\}$ NMR spectrum (C_4D_8O, 298 K, 151.0 MHz) of [(^{Ph3}L)Sr(THF)$_3$] (**7**), formed when [(^{Ph3}L)Sr]$_2$ (**5**) is dissolved in thf. * denotes C_4D_8O.

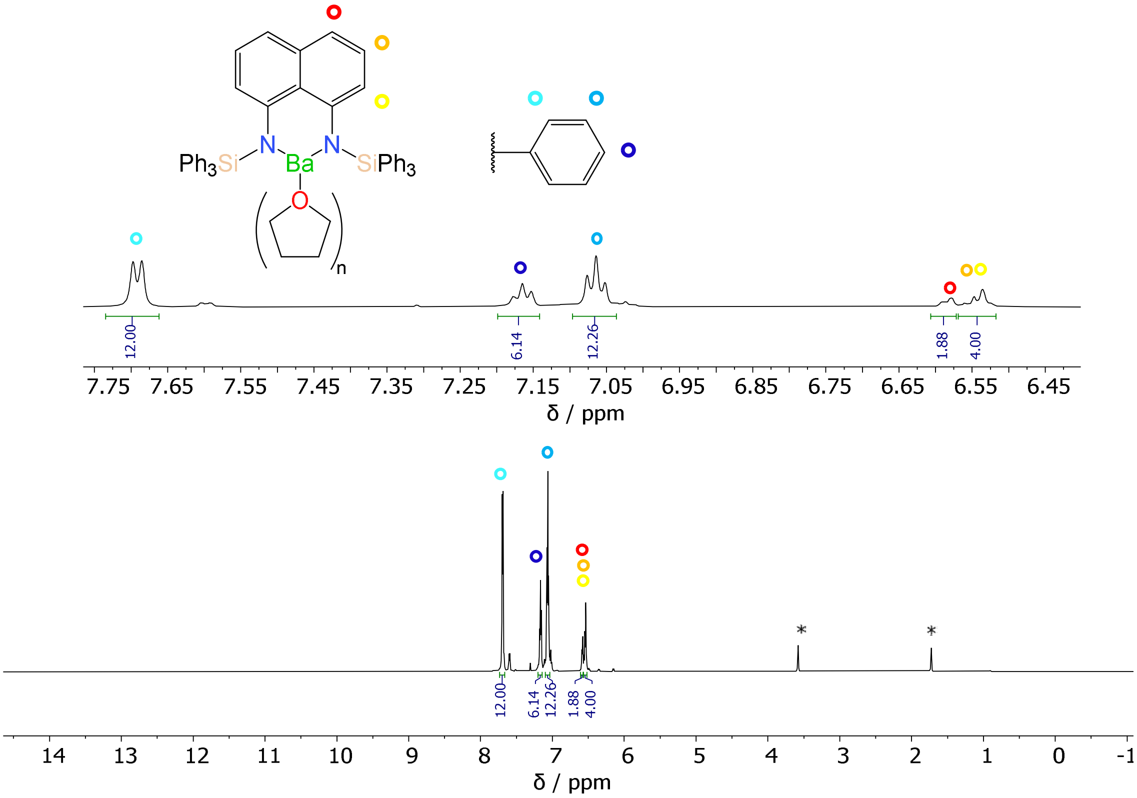

Figure S14. ^{1}H NMR spectrum (C_4D_8O, 298 K, 600.4 MHz) of [(^{Ph3}L)Ba(thf)$_n$], formed when [(^{Ph3}L)Ba]$_2$ (**6**) is dissolved in thf. * denotes residual protio fraction of C_4D_8O. Expansion shows aromatic region.

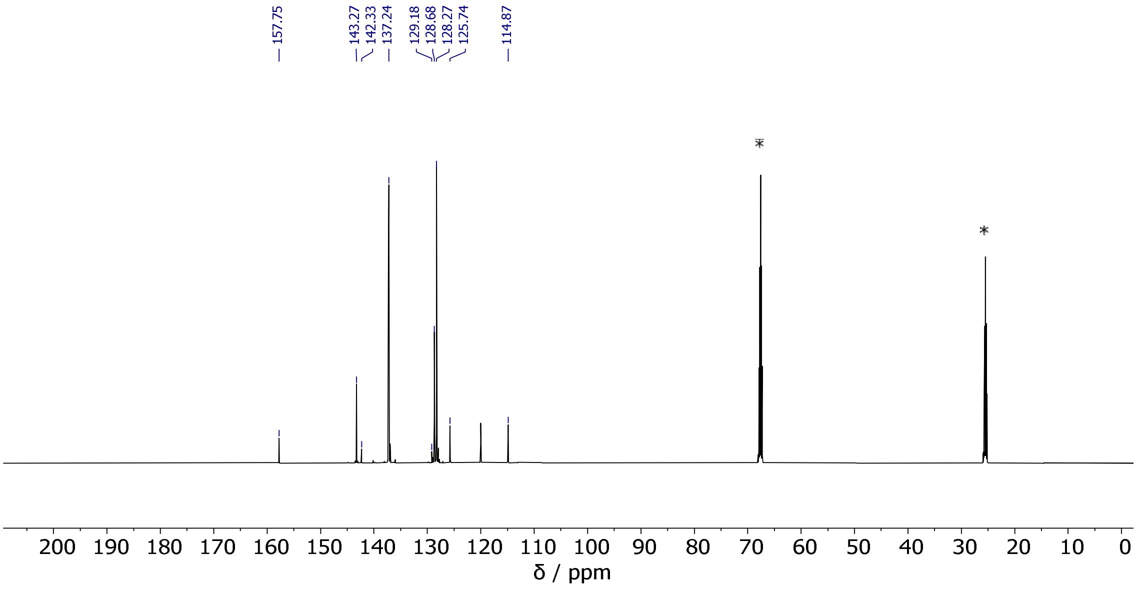

Figure S15. $^{13}C\{^{1}H\}$ NMR spectrum (C_4D_8O, 298 K, 151.0 MHz) of [(^{Ph3}L)Ba(thf)$_n$], formed when [(^{Ph3}L)Ba]$_2$ (**11**) is dissolved in thf. * denotes C_4D_8O.

Table S1. Chemical shifts of the in situ SiR_3 ^{29}Si NMR spectroscopic signals, δ(SiR_3), of [(^{R3}L)Ae(thf)$_n$] (R_3 = Ph_2Me and Ph_3; Ae = Ca, Sr and Ba), formed when **1–6** are dissolved in thf-d_8.

Compound	**Ae**	**R₃**	**δ(SiR₃) / ppm**
**1**	Ca	Ph_2Me	−27.98
**2**	Sr	Ph_2Me	−29.39
**3**	Ba	Ph_2Me	−30.64
**4**	Ca	Ph_3	−31.57
**5**	Sr	Ph_3	−32.92
**6**	Ba	Ph_3	−34.23

Solution phase NMR spectroscopic analysis: *in situ* studies. The insolubility of crystalline samples of **1–6** in benzene and toluene precluded the recording of informative NMR spectra at room temperature. While heating arene suspensions of **1–6** to 80 °C did enable their dissolution in some cases, this unfortunately resulted in decomposition. However, the 2:1 reaction of $^{R3}LH_2$ (R_3 = Ph_2Me and Ph_3) with [AeN''$_2$]$_2$ (Ae = Mg, Ca, Sr and Ba) in C_6D_6 could be monitored by ^1H NMR spectroscopy *in situ*, providing insight into the mechanism by which **1–6** form (**Scheme S1**).

Scheme S1. Synthesis of [(^{R3}L)Ae]$_2$ (**1–6**) *via* the proposed intermediate species [(^{R3}L)Ae_2N''$_2$] (R_3 = Ph_2Me and Ph_3; Ae = Mg, Ca, Sr and Ba).

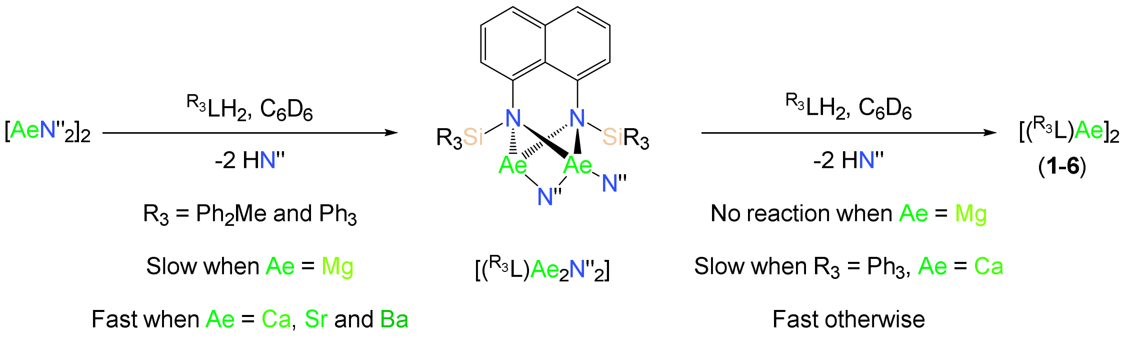

In all cases, no intermediate species with high frequency (N,N)μ_2-H signals or thermal degradation products were observed. With the exception of **4** (*vide infra*), initial ^1H NMR spectra recorded after *ca*. 10 minutes showed that in all cases full deprotonation of both equivalents of $^{R3}LH_2$ had occurred. The rapid crystallisation of **1**, **2**, **3**, **5** and **6** then resulted in spectra with broad, low intensity ligand signals that were dominated by a sharp singlet at δ = 0.10 ppm corresponding to the by-product HN''. However, [(^{Ph3}L)Ca]$_2$ (**4**) forms more slowly and remains dissolved in solution, providing insight into the mechanism of its formation and enabling its characterisation by ^1H NMR spectroscopy. An initial spectrum recorded after *ca.* 10 minutes showed the formation of a mixture of the product **4**, an intermediate species assigned as [(^{Ph3}L)Ca_2N''$_2$] and some as-yet unconsumed proligand. Full conversion to **4** was then achieved after 16 hours at 25 °C. This suggests that while the initial 1:1 reaction between $^{Ph3}LH_2$ and [CaN''$_2$]$_2$ proceeds rapidly, steric congestion results in a substantially slower reaction between the resulting intermediate species [(^{Ph3}L)Ca_2N''$_2$] and the second equivalent of proligand.

The intermediate species [(^{Ph3}L)$Ca_2N''_2$] was synthesised rationally *via* the 1:1 reaction of $^{Ph3}LH_2$ with $[CaN''_2]_2$ in toluene-d_8. A ^{1}H NMR spectrum recorded at 25 °C (**Figure S16a**) notably contains two broad overlapping singlets at δ = –0.28 and –0.43 ppm with a combined integral value of 36, which are assigned as the methyl protons of the two coordinated $[N'']^-$ ligands. This suggests that in solution [(^{Ph3}L)$Ca_2N''_2$] has a fluxional structure in which the two monodentate amide ligands are exchanging between two positions. This is further supported by the presence of only three naphthyl (δ = 7.51, 7.13 and 6.77 ppm) and three phenyl signals (δ = 7.68, 7.27 and 7.18 ppm) in the aromatic region, consistent with the presence of a plane of symmetry through the C(9)-C(10) axis that is perpendicular to the naphthalene ring. An analogous spectrum recorded at 0 °C (**Figure S16b**) shows the divergence of the $[N'']^-$ signal into two sharp singlets. These signals further diverge and sharpen at lower temperatures as the rate of exchange between terminal and bridging positions slows and an asymmetric structure becomes observable ($\delta_{193\,K}$ = –0.13 and –0.47 ppm, T_c = 303 K, Δν = 170 Hz, $\Delta G^{\ddagger}$(303 K) = 59 kJ mol^{-1}).[5] However, the C_2 symmetry of the phenyl and naphthyl signals is retained even upon cooling to –80 °C, with no signal divergence observed. This suggests that the intermediate possesses a Ca_2N_2 ring that is bridged by the $[^{Ph3}L]^{2-}$ ligand, which coordinates to both metal cations in κ^2-N,N′-bidentate manner *via* two μ_2-N donors in order to maximise the coordination number of the two Ca^{2+} centres.

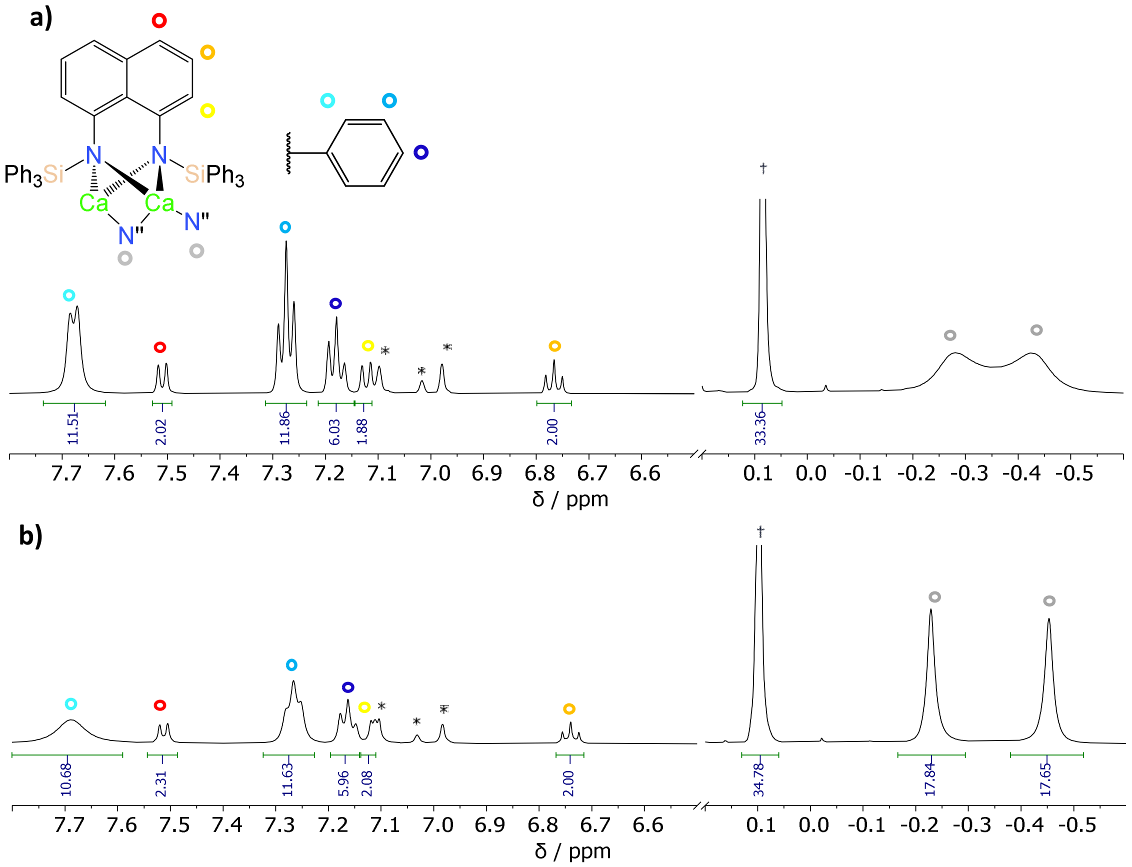

Figure S16. ^{1}H *in situ* ^{1}H NMR spectra (C_7D_8, 499.9 MHz) of [(^{Ph3}L)$Ca_2N''_2$], prepared *via* the 1:1 reaction of $^{Ph3}LH_2$ and $[CaN''_2]_2$, at **a)** 298 K and **b)** 273 K. † denotes HN", * denotes residual protio fraction of C_7D_8.

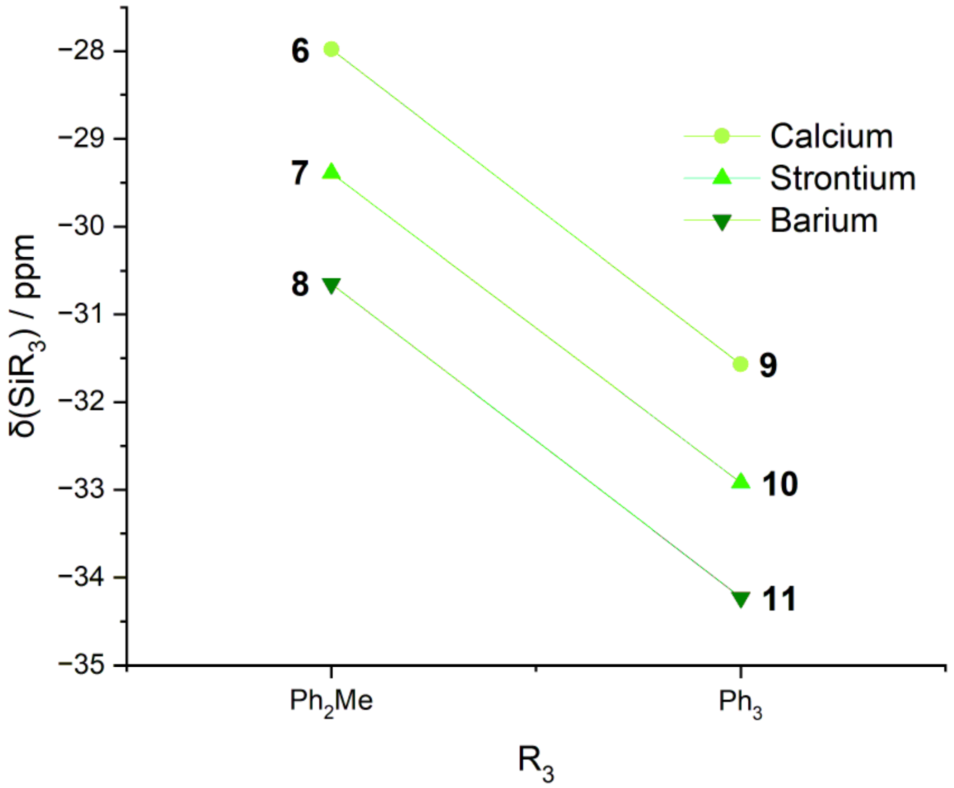

Figure S17. Chemical shifts of the *in situ* SiR_3 ^{29}Si NMR spectroscopic signals, $\delta(SiR_3)$, of $[(^{R3}L)Ae(thf)_n]$ (R_3 = Ph_2Me and Ph_3; Ae = Ca, Sr and Ba), formed when **1**–**6** are dissolved in thf-d_8.

III. Crystallographic data

Crystals were mounted on MiTeGen MicroMounts using perfluoropolyether oil and rapidly transferred to a goniometer head on a diffractometer fitted with an Oxford Cryostream open-flow nitrogen cooling device.[6] Data collections were carried out at 100 or 150 K using an Oxford Diffraction Supernova or Rigaku XtaLAB Synergy DW diffractometer using mirror-monochromated Cu Kα radiation (λ = 1.54178 Å) or Mo Kα radiation (λ = 0.71073 Å) and the data processed using CrysAlisPro.[7] The structures were solved using direct methods (SIR-92),[8] a flipping algorithm (SUPERFLIP),[9] or intrinsic phasing (SHELXT)[10] and refined on F^2 by full-matrix least squares procedures using the WinGX software suite,[11] CRYSTALS,[12] or the SHELXL[13] refinement package implemented in Olex2.[18] Molecular structures were generated using ORTEP (with ellipsoids shown at 30% probability).[14] Geometric calculations were performed using PLATON.[15] Solid state structural data have been deposited in the Cambridge Crystallographic Data Centre (2403499–2403506).

Table S2. Selected experimental crystallographic data.

Complex	^{Ph3}**LH$_2$**	[(^{Ph2Me}L)Ca]$_2$ (**1**)	[(^{Ph2Me}L)Sr]$_2$ (**2**)
**Crystal data**			
Chemical formula	$C_{46}H_{38}N_2Si_2$	$C_{36}H_{32}CaN_2Si_2\cdot(C_6H_6)$	$C_{72}H_{64}N_4Si_4Sr_2\cdot 2(C_6H_6)$
M_r	674.99	667.00	1429.09
Crystal system, space group	Triclinic, $P\bar{1}$	Monoclinic, $P2_1/c$	Monoclinic, $P2_1/c$
Temperature (K)	150	100	100
a, b, c (Å)	11.3470 (3), 13.2059 (3), 24.8531 (5)	10.6141 (4), 30.2492 (9), 11.3302 (3)	10.8034 (4), 30.4356 (9), 11.2445 (3)
α, β, γ (°)	81.2550 (17), 77.0739 (19), 82.0233 (18)	90, 109.192 (4), 90	110.096 (3)
V (Å^3)	3566.32 (15)	3435.6 (2)	3472.2 (2)
Z	4	4	2
Radiation type	Cu *K*a	Mo *K*a	Mo *K*α
μ (mm^{-1})	1.17	0.29	1.66
Crystal size (mm)	0.16 × 0.15 × 0.03	0.09 × 0.08 × 0.05	0.15 × 0.10 × 0.01
**Data Collection**			
Diffractometer	Oxford Diffraction SuperNova	XtaLAB Synergy R, DW system, HyPix-Arc 150	XtaLAB Synergy DW
Absorption correction	Multi-scan *CrysAlis PRO* (Rigaku Oxford	Multi-scan *CrysAlis PRO* 1.171.42.83a (Rigaku Oxford Diffraction, 2023) Empirical	Multi-scan *CrysAlis PRO* (Rigaku Oxford

	Diffraction, 2017)	absorption correction using spherical harmonics, implemented in SCALE3 ABSPACK scaling algorithm.	Diffraction, 2017)
T_{min}, T_{max}	0.86, 0.97	0.970, 1.000	0.74, 0.98
No. of measured, independent and observed [$I > 2\sigma(I)$] reflections	57465, 14727, 11823	41333, 7876, 6624	34757, 7958, 6079
R_{int}	0.048	0.041	0.072
**Refinement**			
$R[F^2 > 2\sigma(F^2)]$, $wR(F^2)$, S	0.049, 0.136, 1.03	0.058, 0.139, 1.16	0.049, 0.128, 0.99
No. of reflections	14724	7876	7958
No. of parameters	917	481	461
No. of restraints	16	264	101
$(\Delta/\sigma)_{max}$	0.002	0.001	0.001
$\Delta\rho_{max}$, $\Delta\rho_{min}$ (e Å^{-3})	0.70, −0.50	0.52, −0.29	0.86, -0.67

Complex	$[(^{Ph_2Me}L)Ba]_2$ **(3)**	$[(^{Ph_3}L)Ca]_2$ **(4)**	$[(^{Ph_3}L)Sr]_2$ **(5)**
**Crystal data**			
Chemical formula	$C_{36}H_{32}BaN_2Si_2{\cdot}2.5(C_6H_6)$	$2(C_{92}H_{72}Ca_2N_4Si_4){\cdot}7(C_6H_6)$	$C_{46}H_{36}N_2Si_2Sr{\cdot}3(C_6H_6)$
M_r	881.45	3399.04	994.89
Crystal system, space group	Monoclinic, $P2_1/c$	Triclinic, $P\bar{1}$	Monoclinic, $P2_1/c$
Temperature (K)	150	100	150
a, *b*, *c* (Å)	17.48618 (16), 14.15228 (13), 17.31503 (17)	13.2209 (1), 26.0135 (2), 27.9342 (3)	16.9507 (2), 17.3350 (2), 17.6684 (2)
α, *β*, *γ* (°)	90, 98.5389 (9), 90	72.1937 (8), 86.2967 (8), 76.4981 (8)	90, 99.032 (1), 90
V (Å^3)	4237.44 (7)	8893.92 (14)	5127.32 (10)
Z	4	2	4
Radiation type	Cu *K*a	Mo *K*α	Cu *K*a
μ (mm^{-1})	8.11	0.24	2.23
Crystal size (mm)	0.17 × 0.11 × 0.03	0.22 × 0.13 × 0.11	0.15 × 0.09 × 0.03
**Data Collection**			
Diffractometer	SuperNova, Dual, Cu at home/near, Atlas	XtaLAB Synergy R, DW system, HyPix-Arc 150	SuperNova, Dual, Cu at home/near, Atlas
Absorption correction	Gaussian *CrysAlis PRO* 1.171.42.72a (Rigaku Oxford Diffraction, 2022) Numerical absorption correction based on gaussian	Multi-scan *CrysAlis PRO* (Rigaku Oxford Diffraction, 2017)	Multi-scan *CrysAlis PRO* 1.171.42.72a (Rigaku Oxford Diffraction, 2022) Empirical absorption correction using spherical

	integration over a multifaceted crystal model Empirical absorption correction using spherical harmonics, implemented in SCALE3 ABSPACK scaling algorithm.		harmonics, implemented in SCALE3 ABSPACK scaling algorithm.
T_{min}, T_{max}	0.425, 0.893	0.84, 0.97	0.815, 1.000
No. of measured, independent and observed [$I > 2\sigma(I)$] reflections	29177, 8741, 7564	186494, 44112, 34971	65375, 10679, 8452
R_{int}	0.046	0.036	0.073
**Refinement**			
$R[F^2 > 2\sigma(F^2)]$, $wR(F^2)$, S	0.033, 0.090, 1.05	0.045, 0.128, 0.99	0.036, 0.087, 1.02
No. of reflections	8741	44109	10679
No. of parameters	507	2215	622
No. of restraints	-	294	-
$(\Delta/\sigma)_{max}$	0.0002	0.003	0.003
$\Delta\rho_{max}$, $\Delta\rho_{min}$ (e Å^{-3})	1.27, −0.96	0.70, −0.43	0.52, −0.34

Complex	$[(^{Ph3}L)Ba]_2$ (**6**)	$[(^{Ph3}L)Sr(thf)_3]$ (**7**)
**Crystal data**		
Chemical formula	$C_{92}H_{72}Ba_2N_4Si_4 \cdot 6(C_6H_6)$	$2(C_{58}H_{60}N_2O_3Si_2Sr) \cdot C_4H_8O$
M_r	2089.20	2025.94
Crystal system, space group	Monoclinic, $P2_1/c$	Monoclinic, $P2_1/c$
Temperature (K)	100	150
a, b, c (Å)	16.8047 (2), 17.5671 (2), 17.4731 (2)	24.4000 (2), 24.0908 (1), 21.8552 (1)
α, β, γ (°)	97.8288 (12)	114.271 (1)
V (Å^3)	5110.15 (10)	11711.31 (15)
Z	2	4
Radiation type	Mo $K\alpha$	Cu $K\alpha$
μ (mm^{-1})	0.87	2.00
Crystal size (mm)	0.13 × 0.11 × 0.05	0.27 × 0.17 × 0.13
**Data Collection**		
Diffractometer	XtaLAB Synergy R, DW system, HyPix-Arc 150	SuperNova, Dual, Cu at home/near, Atlas
Absorption correction	Multi-scan *CrysAlis PRO* (Rigaku Oxford Diffraction, 2017)	Gaussian, CrysAlisPro 1.171.43.90 (Rigaku Oxford Diffraction, 2023) Numerical absorption correction based on gaussian integration over

		a multifaceted crystal model<br>Empirical absorption correction using spherical harmonics, implemented in SCALE3 ABSPACK scaling algorithm.
T_{min}, T_{max}	0.84, 0.96	0.403, 1.000
No. of measured, independent and observed [$I > 2\sigma(I)$] reflections	98152, 12688, 10715	274590, 24454, 23052
R_{int}	0.043	0.027
**Refinement**		
$R[F^2 > 2\sigma(F^2)]$, $wR(F^2)$, S	0.043, 0.129, 1.03	0.029, 0.084, 1.02
No. of reflections	12684	24454
No. of parameters	622	1265
No. of restraints	-	6
$(\Delta/\sigma)_{max}$	0.002	0.005
$\Delta\rho_{max}$, $\Delta\rho_{min}$ (e Å^{-3})	3.39, -0.88	0.76, −0.62

Computer programs: XtaLAB Synergy, Dualflex, HyPix, (Rigaku, 2021), *CrysAlis PRO* 1.171.39.46 (Rigaku Oxford Diffraction, 2018) *CrysAlis PRO*, Agilent Technologies, Version 1.171.35.21 (release 20-01-2012 CrysAlis171 .NET) (compiled Jan 23 2012,18:06:46), : SUPERFLIP. Palatinus, L.; Chapuis, G. J. Appl. Cryst. 2007, 40, 786-790, : Sir-92. Altomare, A.; Cascarano, G.; Giacovazzo, C.; Guagliardi, A. J. Appl. Cryst. 1994, 27, 435., *SIR92* Altomare, A.; Cascarano, G.; Giacovazzo, C.; Guagliardi, A. J. Appl. Cryst. 1994, 27, 435, *SHELXL2018*/3 (Sheldrick, 2018), *SHELXL2014* (Sheldrick, 2014), *ORTEP-3 for Windows*. Farrugia, L. J. J. Appl. Cryst. 1997, 30, 565, *CRYSTALS* (Betteridge *et al.*, 2003), *CAMERON* (Watkin *et al.*, 1996)

Solid state structural analysis of $^{Ph3}LH_2$. Large colourless single crystals of $^{Ph3}LH_2$ suitable for X-ray diffraction analysis grew from a saturated toluene/*n*-pentane solution (**Figure S18**, **Table S3**). $^{Ph3}LH_2$ crystallises in the $P\bar{1}$ space group with two crystallographically independent $^{Ph3}LH_2$ molecules in the asymmetric unit. There is no statistically significant difference between the metrical parameters of these, so only one set of metrics is discussed. Notably, the crystal structure of $^{Ph3}LH_2$ consists of two distinct amino proton environments. One proton sits in the plane of the naphthalene backbone and forms an intramolecular NH--N hydrogen bond between the two amino groups (N(1)-H(2) = 2.11(2) Å, (N(1)-H(2)-N(2) = 140(2)°). Conversely, the other amino proton is projected out of the plane of the naphthalene backbone. This structure agrees well with those reported previously for $^{Ph2Me}LH_2$ and $^{iPr3}LH_2$,[16] and demonstrates the high basicity of the N,N pocket.[17] This contrasts with the C_{2v}-symmetric structures of $^{R3}LH_2$ inferred from NMR spectroscopic analysis, suggesting that in solution the two amino protons rapidly exchange between the two distinct positions they occupy in the solid state.

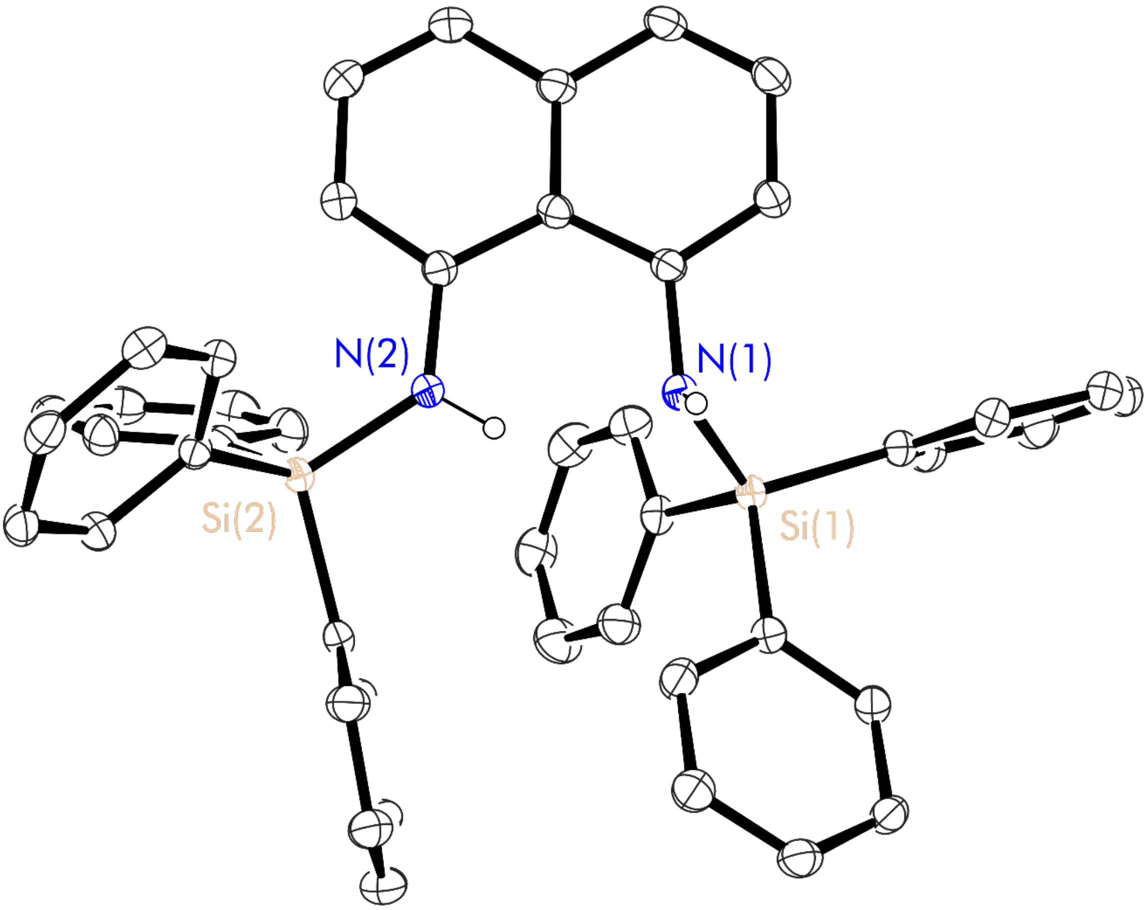

Figure S18. Thermal displacement ellipsoid drawing (30% probability) of $^{Ph3}LH_2$, displaying one of the two $^{Ph3}LH_2$ molecules within the unit cell. All non-amino hydrogen atoms have been omitted for clarity.

Table S3. Representative experimental metrical parameters (bond lengths in Å and angles in °) in one $^{Ph3}LH_2$ molecule in the unit cell.

N(1)-H(1)	0.87(2)
N(2)-H(2)	0.86(2)
N(1)-H(2)	2.11(2)
N(1)-C(1)	1.449(3)
N(2)-C(8)	1.390(3)
N(1)-Si(1)	1.741(2)
N(2)-Si(2)	1.733(2)
N(1)-N(2)	2.827(2)
H(1)-N(1)-C(1)	111(1)
H(1)-N(1)-Si(1)	113(1)
C(1)-N(1)-Si(1)	123.4(1)
H(2)-N(2)-C(8)	115(1)
H(2)-N(2)-Si(2)	117(1)
C(8)-N(2)-Si(2)	127.7(2)
N(1)-H(2)-N(2)	140(2)

Table S4. Selected experimental metrical parameters (bond lengths (Å) and angles (°)) for $[(^{Ph3}L)Sr(thf)_3]$ (**7**). Analogous metrics for **5** and $[(^{Ph2Me}L)Mg(thf)_2]$[16]) are also presented for comparison.

	**7**	**5**	**$[(^{Ph2Me}L)Mg(thf)_2]$[26]**
Space group	$P2_1/c$	$P2_1/c$	$P\overline{1}$
Ae(1)-N(1)	2.465(1)	2.462(2)	2.007(8)
Ae(1)-N(2)	2.437(1)	2.445(2)	1.965(6)
Ae(1)-O(1)	2.557(1)	-	2.025(6)
Ae(1)-O(2)	2.571(1)	-	2.010(7)
Ae(1)-O(3)	2.557(2)	-	-
N(1)-N(2)	2.929(2)	2.900(3)	2.940(9)
Ae(1)-plane$_{C(1)-C(10)}$	1.8654(17)	1.210(3)	1.079(8)
N(1)-Ae(1)-N(2)	73.37(4)	72.47(6)	95.5(3)

a)

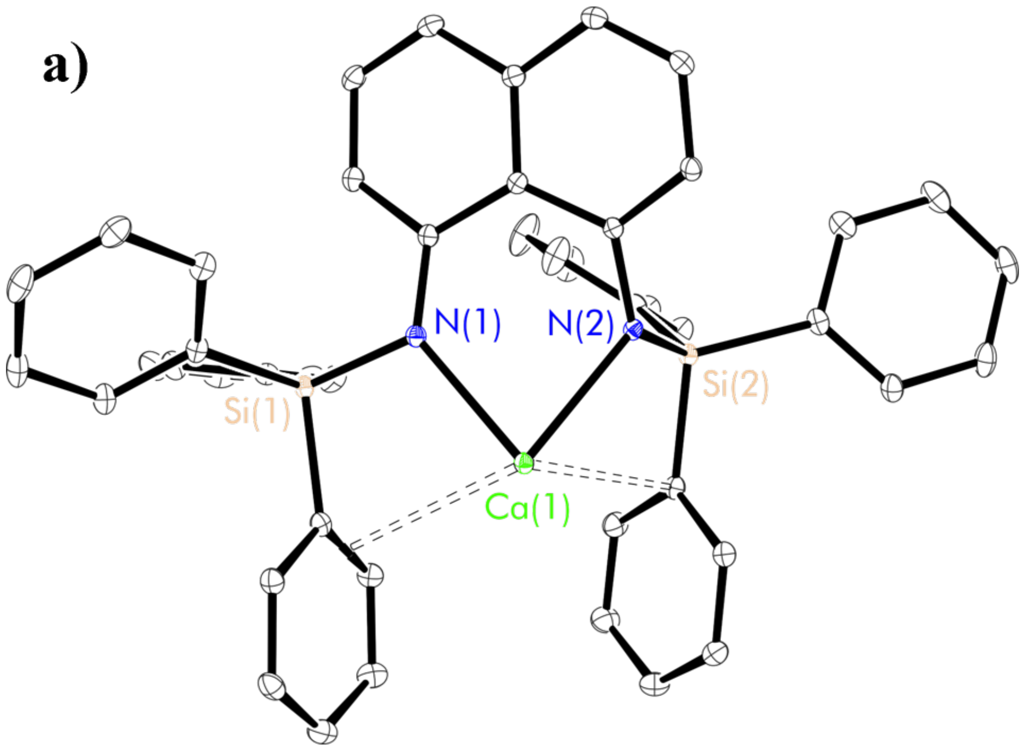

Figure S19. Thermal displacement ellipsoid drawings (30% probability) of the asymmetric [(^{Ph3}L)Ca]$_2$ (**4**). All hydrogen atoms have been omitted for clarity

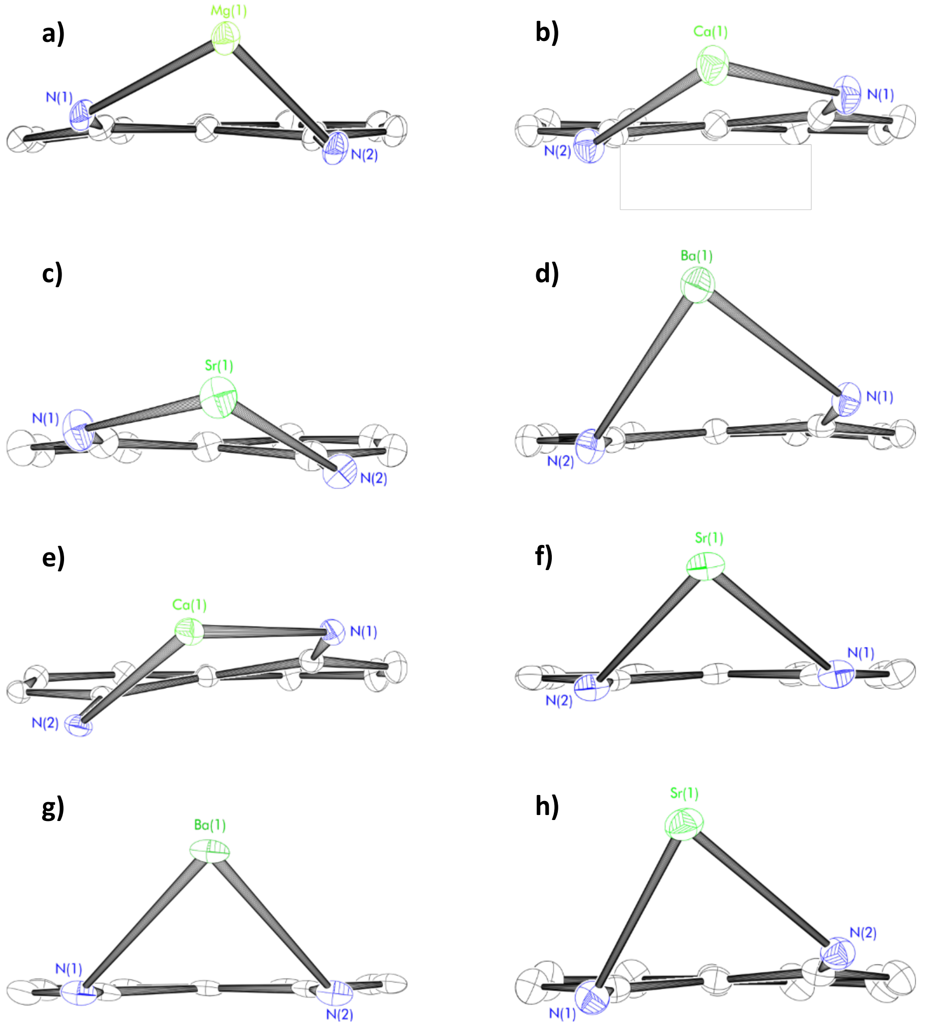

Figure S20. Thermal displacement ellipsoid drawings (30% probability) of the [(1,8-$C_{10}H_6N_2$)Ae] units of **a)** [(^{Ph_2Me}L)Mg(thf)$_2$],[16] **b) 1**, **c) 2**, **d) 3**, **e) 4**, **f) 5**, **g) 6** and **h) 7** viewed down their C(9)-C(10) axes, illustrating the general increase in Ae^{2+} exposure that results from greater Ae^{2+} size. All hydrogen atoms on the fragments have been omitted for clarity.

IV. **Computational Chemistry**

All calculations were performed using the ORCA quantum chemistry software suite. Molecular visualizations, including electron density maps and non-covalent interaction surfaces, were generated using ChimeraX 1.9.[18]

The geometries of compounds **1**–**6** were initially optimized using ORCA 5.0.3[19] at the B3LYP-D3(BJ)/SARC-ZORA-TZVP[20, 21] and r^{2}SCAN-3c[22] levels of theory.[23] Calculations were accelerated using the RIJCOSX approximation.[24] Convergence to true minima was confirmed by the absence of vibrational modes with imaginary frequencies, and the geometries showed a good match to the crystallographic results, measured by root mean squared deviation (RMSD).

Table S5. Comparison of selected experimental metrical parameters (bond lengths (Å) and angles (°)) from complexes **1**–**6** with optimized structures at the B3LYP-D3(BJ)/SARC-ZORA-TZVP[20, 21] and r^{2}SCAN-3c[22] levels of theory.

	**1**			**2**			**3**		
	scXRD	B3LYP	r^{2}SCAN-3c	scXRD	B3LYP	r^{2}SCAN-3c	scXRD	B3LYP	r^{2}SCAN-3c
Ae(1)-N(1)	2.308(2)	2.288	2.302	2.462(3)	2.457	2.459	2.621(2)	2.627	2.637
Ae(1)-N(2)	2.479(2)	2.320	2.325	2.489(2)	2.482	2.482	2.647(2)	2.703	2.694
Ae(1)-N(2)*	2.332(2)	2.451	2.475	2.679(3)	2.649	2.676	3.088(2)	3.028	3.094
Ae(1)-Ae(1)*	3.4870(8)	3.461	3.517	3.7726(4)	3.736	3.806	4.2476(2)	4.184	4.274
N(1)-N(2)	2.956(3)	2.965	2.946	2.983(4)	2.995	2.979	2.935(3)	2.972	2.958
N(1)-Ae(1)-N(2)	79.14(7)	80.08	79.09	74.10(9)	74.64	74.15	67.73(7)	67.76	67.40
N(1)-Ae(1)-N(2)*	117.04(7)	117.22	115.62	114.30(9)	114.17	112.05	104.41(7)	104.77	98.28
N(2)-Ae(1)-N(2)*	87.14(7)	87.04	85.86	86.24(8)	86.62	84.09	84.73(6)	86.38	85.04
RMSD		0.4168	0.3199		0.2944	0.2992		0.2409	0.1903
	**4**			**5**			**6**		
	scXRD	B3LYP	r^{2}SCAN-3c	scXRD	B3LYP	r^{2}SCAN-3c	scXRD	B3LYP	r^{2}SCAN-3c
Ae(1)-N(1)	2.3106(13)	2.298	2.334	2.462(2)	2.468	2.472	2.625(3)	2.652	2.660
Ae(1)-N(2)	2.3568(13)	2.344	2.337	2.445(2)	2.453	2.463	2.593(3)	2.621	2.646
Ae(1)-N(2)*	2.4783(14)	2.440	2.478	4.090(2)	3.976	4.022	4.276(3)	4.258	4.198
Ae(1)-Ae(1)*	3.4486(4)	3.420	3.467	4.8750(5)	4.661	4.855	4.9831(3)	4.893	5.025
N(1)-N(2)	2.9647(19)	2.960	2.953	2.900(3)	2.930	2.911	2.941(4)	2.953	2.940
N(1)-Ae(1)-N(2)	78.86(4)	79.23	78.42	72.47(6)	73.08	72.29	68.60(9)	68.11	67.30
N(1)-Ae(1)-N(2)*	121.18(5)	120.83	119.72	81.57(5)	81.07	79.07	83.18(7)	79.49	79.81
N(2)-Ae(1)-N(2)*	89.04(5)	88.62	87.91	86.95(5)	90.29	86.17	90.45(7)	92.60	88.44
RMSD		0.4357	0.2713		0.1389	0.1421		0.2365	0.1791

Final geometry optimizations were conducted in ORCA 6.0,[19, 25] and wavefunctions were calculated in ORCA 5.0.4 for Energy Decomposition Analysis with Natural Orbitals for Chemical Valence (EDA-NOCV)[26] and Natural Bond Orbital (NBO) analysis.[27] Post-optimization analyses, including Bader charge[28] and non-covalent interaction analyses,[29] were performed using wavefunctions generated in ORCA 6.0.

Geometry Optimization

The molecular geometries were optimized using the meta-generalized gradient approximation (meta-GGA) functional R^{2}SCAN,[30] incorporating D4 dispersion corrections[31] to account for non-covalent interactions. Scalar relativistic effects were treated using the Exact-Two-Component (X2C) Hamiltonian,[32] which decouples the Dirac equation into a two-component form, offering an efficient and accurate description of relativistic effects. The X2C-TZVPall basis set was applied to all atoms except hydrogen, which was treated with the X2C-SVPall basis set.[33] To ensure the accuracy of the optimizations, extremely tight convergence criteria for geometry (verytightopt) and self-consistent field (SCF) calculations (verytightscf), combined with a high-density integration grid (defgrid3), were

employed. These settings eliminated small imaginary frequencies, confirming the optimized structures as true minima. The Cartesian coordinates of the optimized geometries of **1-7** are available, as well as the monomeric fragments derived from **1-6**.

Bader Charge and Non-Covalent Interaction Analyses

Bader charge analysis, based on the Quantum Theory of Atoms in Molecules (QTAIM), was performed using the Multiwfn program with a high grid density.[34] This method partitions the electron density into atomic basins delineated by zero-flux surfaces in the density gradient, providing a rigorous and intuitive measure of charge distribution. Bader charges offer quantitative insights into electron transfer, polarization, and bonding characteristics within molecular systems.

The Independent Gradient Model (IGM) was applied,[29] using the Multiwfn program, to investigate non-covalent interactions. This method quantifies weak intermolecular and intramolecular interactions by analysing the gradient of the electron density. Unlike topological approaches such as QTAIM, IGM focuses on the overlap of electron density gradients, making it particularly effective for identifying and characterizing interactions such as van der Waals forces, hydrogen bonds, and π-π stacking. Key interaction regions were visualized using isosurfaces, with color-coded maps indicating stabilizing and destabilizing interactions.

Natural Bond Orbital (NBO) and Natural Orbitals for Chemical Valence (NOCV) Analyses

NBO and NOCV analyses were performed using wavefunctions generated in ORCA 5.0.4.[26, 27] Scalar relativistic effects were treated with the Zero-Order Regular Approximation (ZORA).[35] The SARC-ZORA-TZVP basis set was used for the metal centre,[36] while the def2-TZVP basis set was applied to all other atoms.[21, 37] Dispersion interactions were included via the D4 correction.[31]

NBO analysis identifies and quantifies localized bonding interactions, such as bonds, lone pairs, and antibonding orbitals, providing a detailed understanding of the electronic structure and bonding framework.

NOCV analysis breaks down the orbital component of the interaction energy into chemically meaningful descriptors. These descriptors elucidate the key orbital interactions that drive bond formation, offering insights into the electronic factors underpinning the stability and reactivity of the system.

1

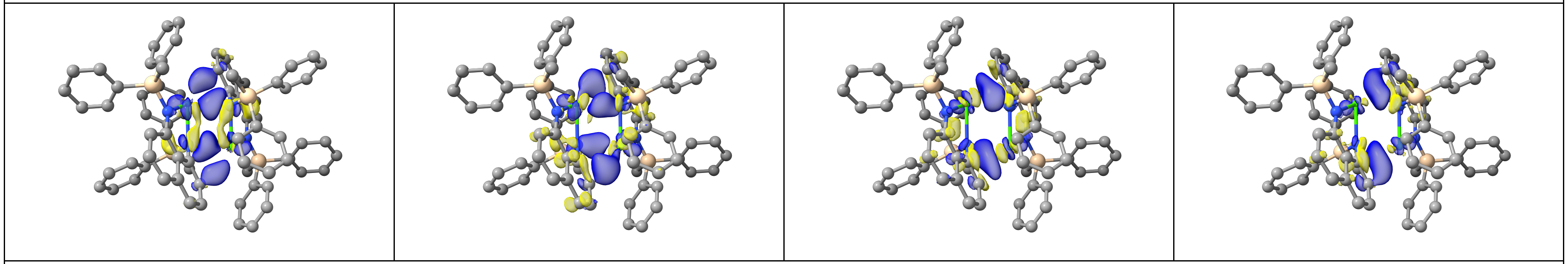

2

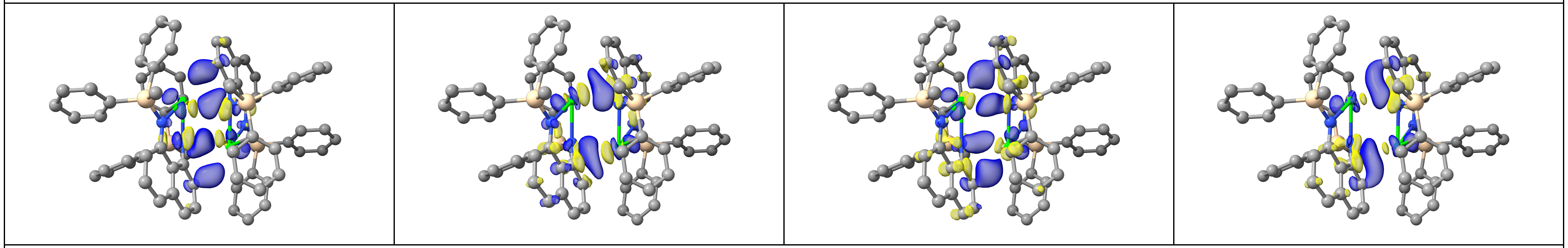

3

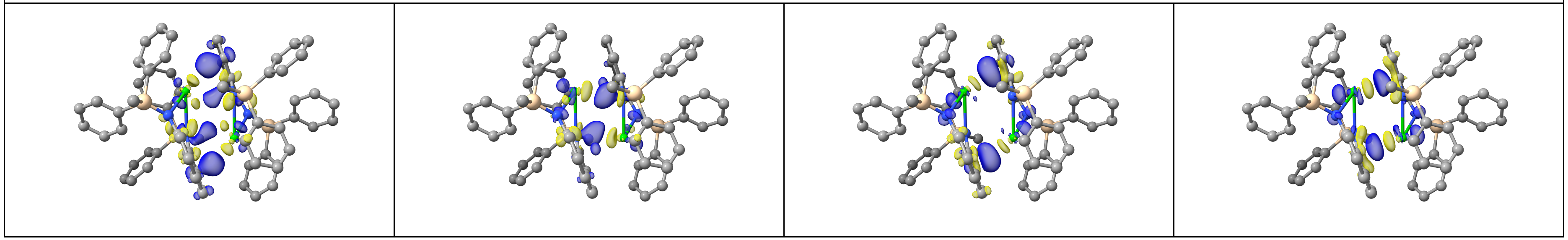

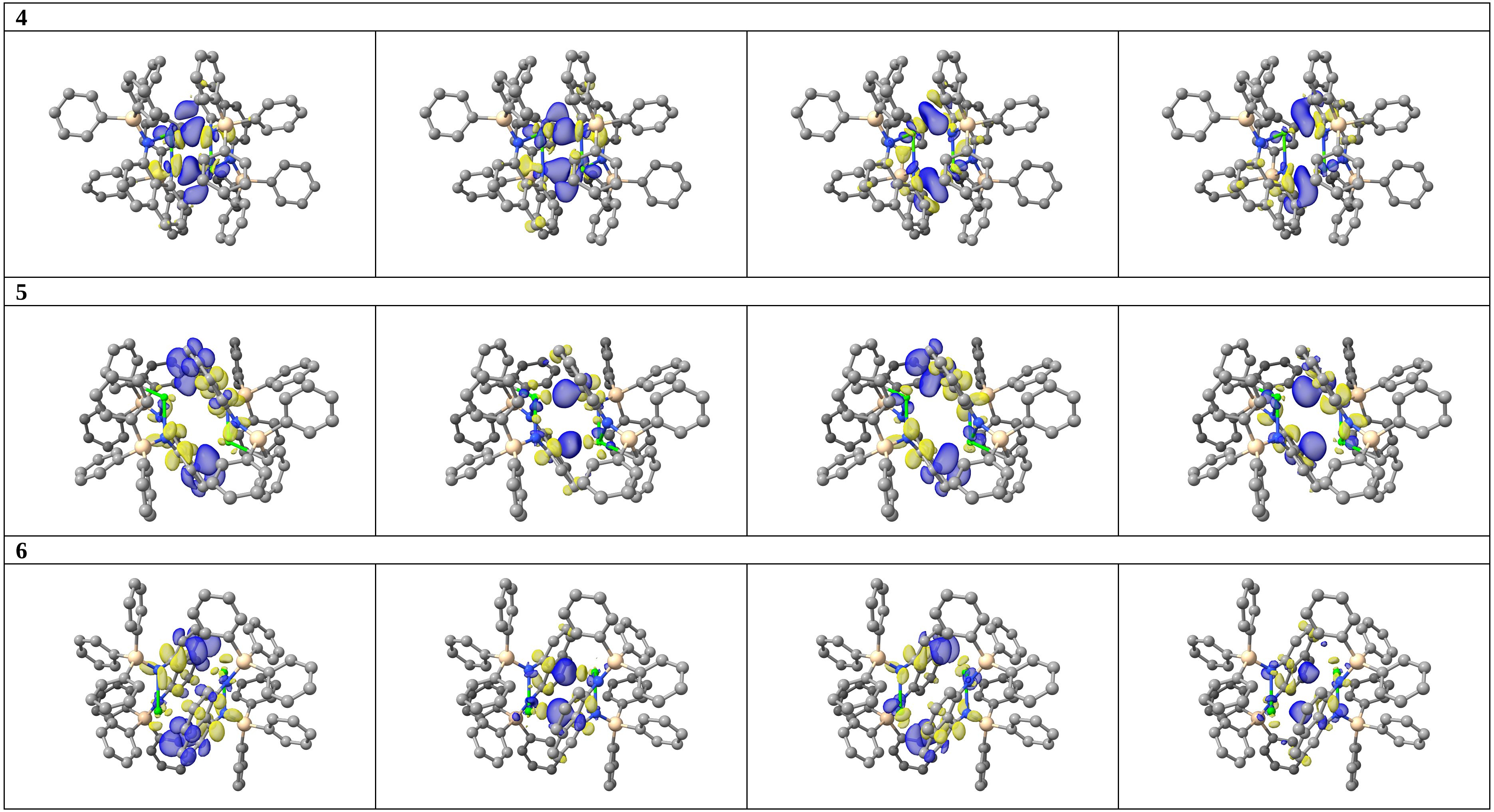

Figure S21. EDA with Natural Orbitals for Chemical Valence (EDA-NOCV) analysis of complexes **1**–**6**, showing the pairwise deformation densities of the largest contributing orbital interactions. Blue and yellow represent donating and accepting orbitals respectively.[38]

Table S6. Energy Decomposition Analysis of dimeric complexes [(^{R3}L)Ae]$_2$ **1**–**6** relative to monomers of the form (^{R3}L)Ae, with energies reported in kcal mol^{-1}. The total interaction energy (E_{int}) between monomers in the dimer is decomposed into the orbital contribution (E_{orb}) arising from covalent bonding contributions and further into the four principal NOCV orbitals with eigenvalue e and energy E (Figure S21 *vide supra*). The overall Gibbs energy of interaction, ΔG, includes entropic and thermal effects.

	**1**		**2**		**3**		**4**		**5**		**6**	
ΔG	−55.6993		−54.4260		−53.3339		−57.5757		−59.4528		−52.3005	
E_{int}	−94.1101		−86.7610		−84.1333		−104.496		−90.3569		−82.5277	
E_{orb}	−59.436		−53.798		−51.844		−63.093		−54.098		−48.279	
E_{orb}/E_{int}	0.6316		0.6201		0.6162		0.6038		0.5987		0.5850	
NOCV	e	E	e	E	e	E	e	E	e	E	e	E
1	0.345273	−12.13	0.335575	−10.38	0.336532	−8.499	0.32424	−11.003	0.32991	−6.801	0.313646	−5.508
2	0.345091	−9.008	0.319739	−7.572	0.274937	−5.522	0.320902	−8.574	0.239833	−5.553	0.256876	−5.513
3	0.236218	−6.185	0.233932	−5.753	0.238507	−4.793	0.243498	−6.168	0.234442	−4.584	0.221758	−4.142
4	0.185659	−4.557	0.168097	−3.845	0.191534	−4.318	0.187212	−4.241	0.212528	−4.654	0.197873	−3.754

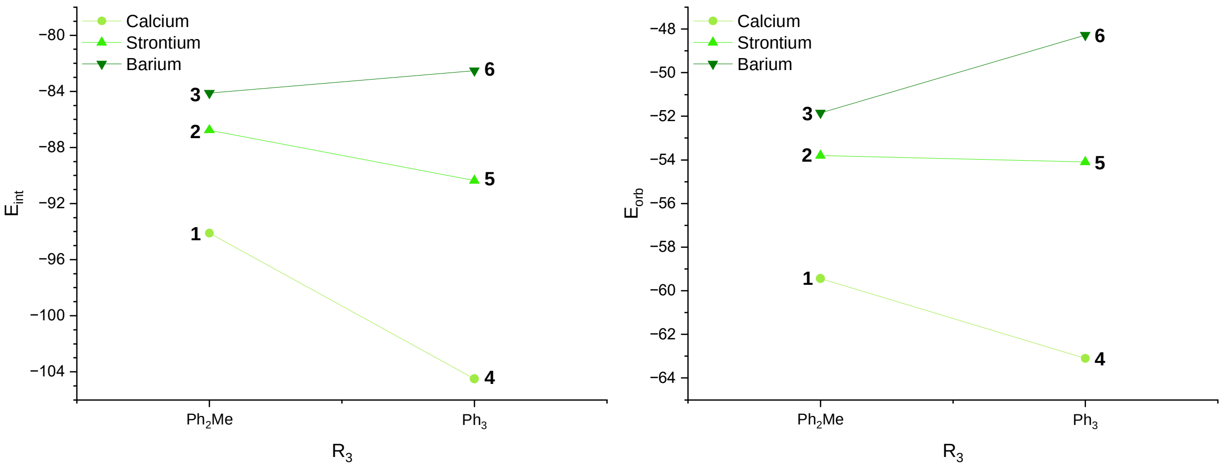

Figure S22 E_{int} and E_{orb} calculated by EDA of complexes **1**–**6**.

As the metal size increases, both E_{int} and E_{orb} become less stabilizing with weaker electrostatic and covalent interactions resulting from decreased charge density and decreased orbital overlap. The ratio E_{orb}/E_{int}, which represents the covalent character of the interactions, decreases both down group two and also with the bulkier ligand. This is consistent with increased steric hinderance in the ^{Ph3}L ligand as well as poorer orbital overlap with the heavier metals. The strontium complexes **2** and **5** have comparable E_{orb}, suggesting that the Ae-arene interaction in the cases balances the steric effects of the bulkier ligand. The barium complexes are relatively destabilized with the ^{Ph3}L ligand compared to the ^{Ph2Me}L as steric repulsion and weak π interactions dominate the covalent interactions.

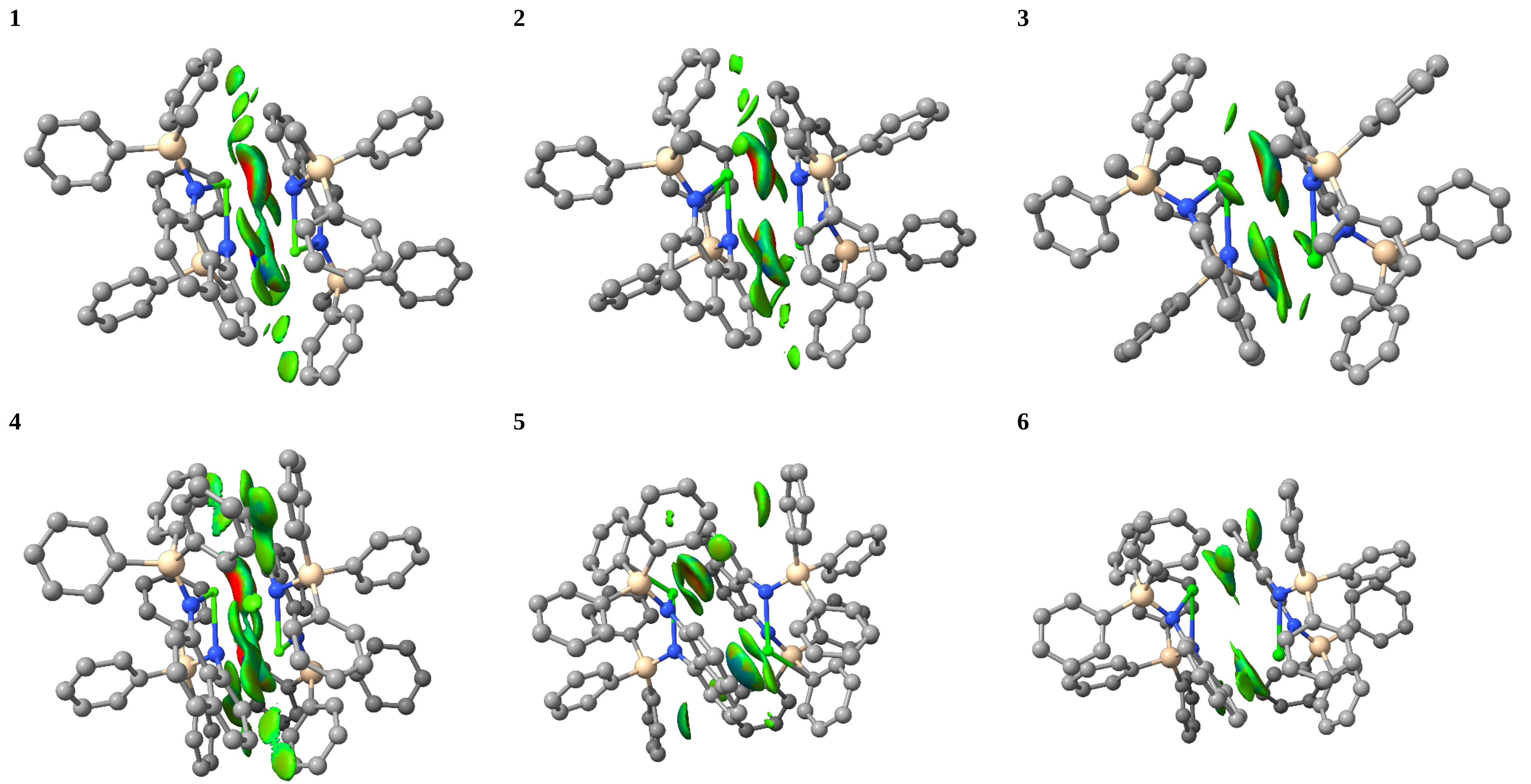

Figure S23. IGM with Hirshfeld Partitioning (IGMH) analysis of non-covalent interactions at the Ae centers in complexes **1**–**6**. Steric repulsive effects (red) of the ligands in the dimer are offset by attractive van der Waals (green) and stronger covalent and ionic (blue) interactions of the naphthyl and phenyl π systems.[39]

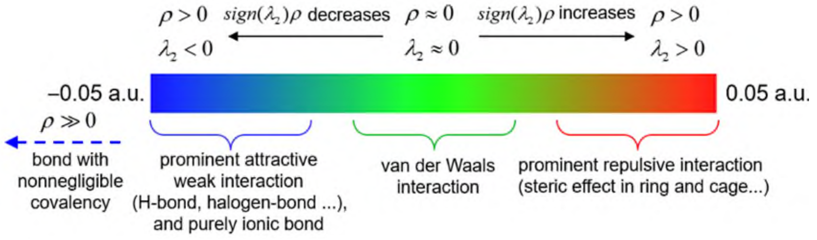

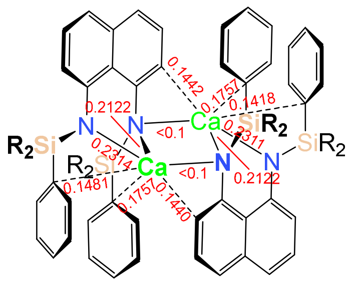

R_3 = Ph_2Me, Ae = Ca (**1**)

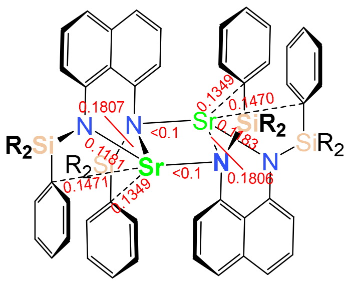

R_3 = Ph_2Me, Ae = Sr (**2**)

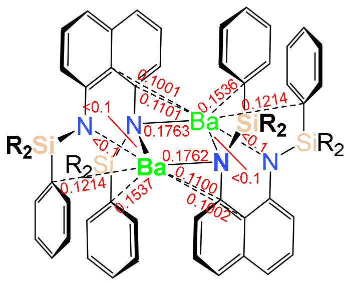

R_3 = Ph_2Me, Ae = Ba (**3**)

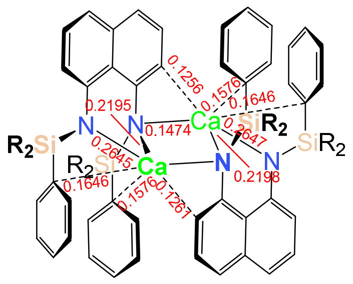

R_3 = Ph_3, Ae = Ca (**4**)

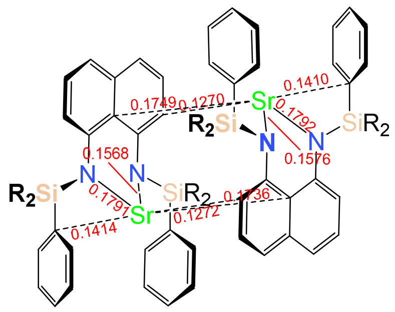

R_3 = Ph_3, Ae = Sr (**5**)

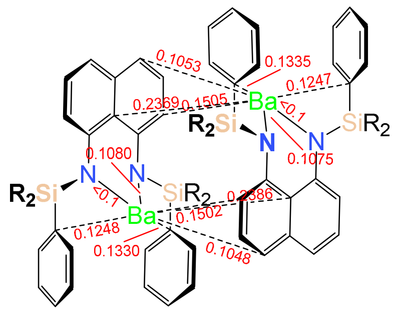

R_3 = Ph_3, Ae = Ba (**6**)

Figure S24. Mayer Bond Order analysis of Ae-arene interactions in **1**–**6**.[40] The calcium complexes are dominated by Ca-N bonding. The barium complexes have weakened Ba-N bonding as a result of poor orbital overlap *vide supra* and the structures are dominated by "soft" π-facial Ba-arene interactions. The strontium complexes **2** and **5** have comparable Sr-N and Sr-arene bond orders, and the preference for "relaxed" or "contracted" structures is finely balanced but ultimately determined by ligand sterics (Figure S23).

Table S7. Mean net charges on Ae, Si, and N in complexes **1**–**6** calculated using both NPA and Bader/QTAIM molecular partition models. Essentially no difference is observed (at most 3–4%) in monomeric species (^{R3}L)Ae compared to the dimers [(^{R3}L)Ae]$_2$ **1**–**6**. These net charges demonstrate the increasingly electron withdrawing nature of ^{Ph3}L compared with ^{Ph2Me}L as well as the increasingly ionic nature of heavier group two metals.

	Ae		Si		N	
Compound	NPA	QTAIM	NPA	QTAIM	NPA	QTAIM
**1**	1.76948	1.53777	1.85053	2.92625	–1.33569	–1.78125
**2**	1.79049	1.53417	1.85147	2.92225	–1.32527	–1.76847
**3**	1.76419	1.51346	1.85722	2.91773	–1.28698	–1.79778
**4**	1.77489	1.54586	1.86964	2.91518	–1.35157	–1.77821
**5**	1.79369	1.54628	1.86506	2.92302	–1.25076	–1.75400
**6**	1.78096	1.51785	1.87087	2.92390	–1.24703	–1.74407

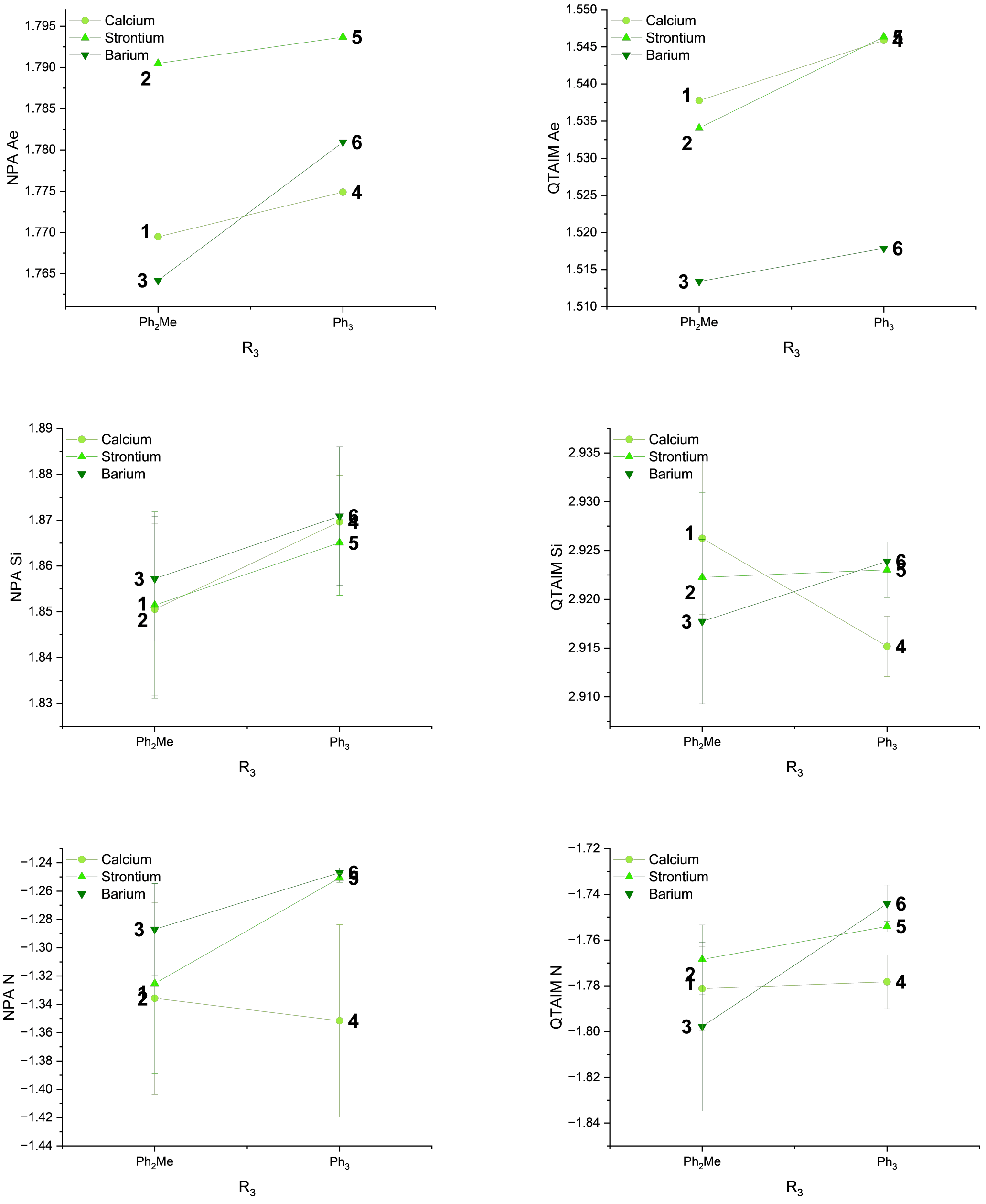

Figure S25. Net charges on Ae, Si, and N (mean ± s.d.) in complexes **1**–**6** calculated using both NPA and Bader/QTAIM molecular partition models.

V. **References**

(1) Gade, L. H.; Galka, C. H.; Hellmann, K. W.; Williams, R. M.; De Cola, L.; Scowen, I. J.; McPartlin, M. Tetraaminoperylenes: Their Efficient Synthesis and Physical Properties. *Chem. Eur. J.* **2002**, *8* (16), 3732-3746. DOI: https://doi.org/10.1002/1521-3765(20020816)8:16%3C3732::AID-CHEM3732%3E3.0.CO;2-5.
(2) Spivey, J. A.; Collum, D. B. Potassium Hexamethyldisilazide (KHMDS): Solvent-Dependent Solution Structures. *J. Am. Chem. Soc.* **2024**, *146* (26), 17827-17837. DOI: 10.1021/jacs.4c03418.
(3) Sarazin, Y.; Howard, R. H.; Hughes, D. L.; Humphrey, S. M.; Bochmann, M. Titanium, zinc and alkaline-earth metal complexes supported by bulky O,N,N,O-multidentate ligands: syntheses, characterisation and activity in cyclic ester polymerisation. *Dalton Trans.* **2006**, (2), 340-350, 10.1039/B511851D. DOI: 10.1039/B511851D. Cameron, T. M.; Xu, C.; Dipasquale, A. G.; Rheingold, A. L. Synthesis and Structure of Strontium and Barium Guanidinates and Mixed-Ligand Guanidinate Pentamethylcyclopentadienyl Complexes. *Organometallics* **2008**, *27* (7), 1596-1604. DOI: 10.1021/om701118j.
(4) Glock, C.; Krieck, S.; Westerhausen, M.; Lavin, C. M.; Gillett-Kunnath, M. M.; Ruhlandt, K.; Hill, M. S.; Anker, M. D.; Wilson, A. S. S.; Weetman, C.; et al. CALCIUM, STRONTIUM, GERMANIUM, TIN, AND LEAD BIS(TRIMETHYLSILYL)AMIDO DERIVATIVES AND 2,2,6,6-TETRAMETHYLPIPERIDIDO AND N-ISOPROPYLPHENYLAMIDO DERVATIVES OF POTASSIUM AND CALCIUM. In *Inorganic Syntheses*, 2018; pp 15-31. Vaartstra, B. A.; Huffman, J. C.; Streib, W. E.; Caulton, K. G. Syntheses and structures of a series of very low coordinate barium compounds: Ba[N(SiMe3)2]2(THF)2, {Ba[N(SiMe3)2)]2(THF)}2, and {Ba[N(SiMe3)2]2}2. *Inorg. Chem.* **1991**, *30* (1), 121-125. DOI: 10.1021/ic00001a023.
(5) Palmer III, A. G. NMR Probes of Molecular Dynamics: Overview and Comparison with Other Techniques. *Annu. Rev. Biophys.* **2001**, *30* (Volume 30, 2001), 129-155. DOI: https://doi.org/10.1146/annurev.biophys.30.1.129.
(6) Cosier, J.; Glazer, A. M. A nitrogen-gas-stream cryostat for general X-ray diffraction studies. *J. Appl. Crystallogr.* **1986**, *19* (2), 105-107. DOI: doi:10.1107/S0021889886089835.
(7) CrysAlisPRO, Oxford Diffraction /Agilent Technologies UK Ltd, Yarnton, England. (accessed.
(8) Altomare, A.; Cascarano, G.; Giacovazzo, C.; Guagliardi, A. Completion and refinement of crystal structures with SIR92. *J. Appl. Crystallogr.* **1993**, *26* (3), 343-350. DOI: doi:10.1107/S0021889892010331.
(9) Palatinus, L.; Chapuis, G. SUPERFLIP - a computer program for the solution of crystal structures by charge flipping in arbitrary dimensions. *J. Appl. Crystallogr.* **2007**, *40* (4), 786-790. DOI: doi:10.1107/S0021889807029238.
(10) Sheldrick, G. SHELXT - Integrated space-group and crystal-structure determination. *Acta Crystallogr., Sect. A: Found. Crystallogr.* **2015**, *71* (1), 3-8. DOI: doi:10.1107/S2053273314026370.
(11) Farrugia, L. WinGX suite for small-molecule single-crystal crystallography. *J. Appl. Crystallogr.* **1999**, *32* (4), 837-838. DOI: doi:10.1107/S0021889899006020.
(12) Betteridge, P. W.; Carruthers, J. R.; Cooper, R. I.; Prout, K.; Watkin, D. J. CRYSTALS version 12: software for guided crystal structure analysis. *J. Appl. Crystallogr.* **2003**, *36* (6), 1487. DOI: doi:10.1107/S0021889803021800.
(13) Sheldrick, G. Crystal structure refinement with SHELXL. *Acta Crystallogr. Sect. C: Cryst. Struct. Commun.* **2015**, *71* (1), 3-8. DOI: doi:10.1107/S2053229614024218.
(14) Farrugia, L. ORTEP-3 for Windows - a version of ORTEP-III with a Graphical User Interface (GUI). *J. Appl. Crystallogr.* **1997**, *30* (5 Part 1), 565. DOI: doi:10.1107/S0021889897003117.
(15) Spek, A. Single-crystal structure validation with the program PLATON. *J. Appl. Crystallogr.* **2003**, *36* (1), 7-13. DOI: doi:10.1107/S0021889802022112.
(16) Bradley, M. A.; Birchall, C.; Blake, A. J.; Lewis, W.; Moxey, G. J.; Kays, D. L. 1,8-Bis(silylamido)naphthalene complexes of magnesium and zinc synthesised through alkane elimination reactions. *Dalton Trans.* **2017**, *46* (12), 4101-4110, 10.1039/C7DT00471K. DOI: 10.1039/C7DT00471K.
(17) Benoit, R. L.; Lefebvre, D.; Fréchette, M. Basicity of 1,8-bis(dimethylamino)naphthalene and 1,4-diazabicyclo[2.2.2]octane in water and dimethylsulfoxide. *Can. J. Chem.* **1987**, *65* (5), 996-1001. DOI: 10.1139/v87-170.

(18) Meng, E. C.; Goddard, T. D.; Pettersen, E. F.; Couch, G. S.; Pearson, Z. J.; Morris, J. H.; Ferrin, T. E. UCSF ChimeraX: Tools for structure building and analysis. *Protein Sci.* **2023**, *32* (11), e4792. DOI: https://doi.org/10.1002/pro.4792. Pettersen, E. F.; Goddard, T. D.; Huang, C. C.; Meng, E. C.; Couch, G. S.; Croll, T. I.; Morris, J. H.; Ferrin, T. E. UCSF ChimeraX: Structure visualization for researchers, educators, and developers. *Protein Sci.* **2021**, *30* (1), 70-82. DOI: https://doi.org/10.1002/pro.3943.
(19) Neese, F. The ORCA program system. *Wiley Interdisciplinary Reviews: Computational Molecular Science* **2012**, *2* (1), 73-78. DOI: doi:10.1002/wcms.81. Neese, F. Software update: The ORCA program system—Version 5.0. *WIREs Computational Molecular Science* **2022**, *12* (5), e1606. DOI: https://doi.org/10.1002/wcms.1606.
(20) Becke, A. D. Density functional calculations of molecular bond energies. *J. Chem. Phys.* **1986**, *84* (8), 4524-4529. DOI: doi:http://dx.doi.org/10.1063/1.450025. Lee, C.; Yang, W.; Parr, R. G. Development of the Colle-Salvetti correlation-energy formula into a functional of the electron density. *Physical Review B* **1988**, *37* (2), 785-789. Grimme, S.; Antony, J.; Ehrlich, S.; Krieg, H. A consistent and accurate ab initio parametrization of density functional dispersion correction (DFT-D) for the 94 elements H-Pu. *J. Chem. Phys.* **2010**, *132* (15). DOI: 10.1063/1.3382344 (acccessed 1/8/2025). Grimme, S.; Ehrlich, S.; Goerigk, L. Effect of the damping function in dispersion corrected density functional theory. *J. Comput. Chem.* **2011**, *32* (7), 1456-1465. DOI: https://doi.org/10.1002/jcc.21759. Weigend, F. Accurate Coulomb-fitting basis sets for H to Rn. *Physical Chemistry Chemical Physics* **2006**, *8* (9), 1057-1065, 10.1039/B515623H. DOI: 10.1039/B515623H.
(21) Weigend, F.; Ahlrichs, R. Balanced basis sets of split valence, triple zeta valence and quadruple zeta valence quality for H to Rn: Design and assessment of accuracy. *Physical Chemistry Chemical Physics* **2005**, *7* (18), 3297-3305, 10.1039/B508541A. DOI: 10.1039/B508541A.
(22) Grimme, S.; Hansen, A.; Ehlert, S.; Mewes, J.-M. r2SCAN-3c: A "Swiss army knife" composite electronic-structure method. *J. Chem. Phys.* **2021**, *154* (6). DOI: 10.1063/5.0040021 (acccessed 1/8/2025).
(23) Caldeweyher, E.; Bannwarth, C.; Grimme, S. Extension of the D3 dispersion coefficient model. *J. Chem. Phys.* **2017**, *147* (3). DOI: 10.1063/1.4993215 (acccessed 1/8/2025). Kaupp, M.; Schleyer, P. v. R.; Stoll, H.; Preuss, H. Pseudopotential approaches to Ca, Sr, and Ba hydrides. Why are some alkaline earth MX2 compounds bent? *J. Chem. Phys.* **1991**, *94* (2), 1360-1366. DOI: 10.1063/1.459993 (acccessed 1/8/2025).
(24) Neese, F.; Wennmohs, F.; Hansen, A.; Becker, U. Efficient, approximate and parallel Hartree–Fock and hybrid DFT calculations. A 'chain-of-spheres' algorithm for the Hartree–Fock exchange. *Chem. Phys.* **2009**, *356* (1), 98-109. DOI: https://doi.org/10.1016/j.chemphys.2008.10.036. Izsák, R.; Neese, F. An overlap fitted chain of spheres exchange method. *J. Chem. Phys.* **2011**, *135* (14). DOI: 10.1063/1.3646921 (acccessed 1/8/2025).
(25) Neese, F. An improvement of the resolution of the identity approximation for the formation of the Coulomb matrix. *J. Comput. Chem.* **2003**, *24* (14), 1740-1747. DOI: https://doi.org/10.1002/jcc.10318. Garcia-Ratés, M.; Neese, F. Effect of the Solute Cavity on the Solvation Energy and its Derivatives within the Framework of the Gaussian Charge Scheme. *J. Comput. Chem.* **2020**, *41* (9), 922-939. DOI: https://doi.org/10.1002/jcc.26139. Neese, F. The SHARK integral generation and digestion system. *J. Comput. Chem.* **2023**, *44* (3), 381-396. DOI: https://doi.org/10.1002/jcc.26942.
(26) Mitoraj, M. P.; Michalak, A.; Ziegler, T. A Combined Charge and Energy Decomposition Scheme for Bond Analysis. *Journal of Chemical Theory and Computation* **2009**, *5* (4), 962-975. DOI: 10.1021/ct800503d. Michalak, A.; Mitoraj, M.; Ziegler, T. Bond Orbitals from Chemical Valence Theory. *J. Phys. Chem. A* **2008**, *112* (9), 1933-1939. DOI: 10.1021/jp075460u.
(27) *NBO 7.0*; Theoretical Chemistry Institute, University of Wisconsin, Madison: 2018. (accessed.
(28) Bader, R. F. W. The Quantum Mechanical Basis of Conceptual Chemistry. *Monatshefte für Chemie / Chemical Monthly* **2005**, *136* (6), 819-854. DOI: 10.1007/s00706-005-0307-x. Bader, R. F. W.; Hernández-Trujillo, J.; Cortés-Guzmán, F. Chemical bonding: From Lewis to atoms in molecules. *J. Comput. Chem.* **2007**, *28* (1), 4-14. DOI: https://doi.org/10.1002/jcc.20528.
(29) Wieduwilt, E. K.; Boisson, J.-C.; Terraneo, G.; Hénon, E.; Genoni, A. A Step toward the Quantification of Noncovalent Interactions in Large Biological Systems: The Independent Gradient Model-Extremely Localized Molecular Orbital Approach. *Journal of Chemical Information and Modeling* **2021**, *61* (2), 795-809. DOI: 10.1021/acs.jcim.0c01188.

(30) Furness, J. W.; Kaplan, A. D.; Ning, J.; Perdew, J. P.; Sun, J. Accurate and Numerically Efficient r2SCAN Meta-Generalized Gradient Approximation. *The Journal of Physical Chemistry Letters* **2020**, *11* (19), 8208-8215. DOI: 10.1021/acs.jpclett.0c02405.
(31) Caldeweyher, E.; Ehlert, S.; Hansen, A.; Neugebauer, H.; Spicher, S.; Bannwarth, C.; Grimme, S. A generally applicable atomic-charge dependent London dispersion correction. *J. Chem. Phys.* **2019**, *150* (15). DOI: 10.1063/1.5090222 (acccessed 1/8/2025).
(32) Peng, D.; Middendorf, N.; Weigend, F.; Reiher, M. An efficient implementation of two-component relativistic exact-decoupling methods for large molecules. *J. Chem. Phys.* **2013**, *138* (18). DOI: 10.1063/1.4803693 (acccessed 1/8/2025).
(33) Pollak, P.; Weigend, F. Segmented Contracted Error-Consistent Basis Sets of Double- and Triple-ζ Valence Quality for One- and Two-Component Relativistic All-Electron Calculations. *Journal of Chemical Theory and Computation* **2017**, *13* (8), 3696-3705. DOI: 10.1021/acs.jctc.7b00593.
(34) Lu, T.; Chen, F. Multiwfn: A multifunctional wavefunction analyzer. *J. Comput. Chem.* **2012**, *33* (5), 580-592. DOI: https://doi.org/10.1002/jcc.22885. Lu, T. A comprehensive electron wavefunction analysis toolbox for chemists, Multiwfn. *J. Chem. Phys.* **2024**, *161* (8). DOI: 10.1063/5.0216272 (acccessed 1/8/2025).
(35) van Lenthe, E.; Snijders, J. G.; Baerends, E. J. The zero-order regular approximation for relativistic effects: The effect of spin–orbit coupling in closed shell molecules. *J. Chem. Phys.* **1996**, *105* (15), 6505-6516. DOI: doi:http://dx.doi.org/10.1063/1.472460.
(36) Rolfes, J. D.; Neese, F.; Pantazis, D. A. All-electron scalar relativistic basis sets for the elements Rb–Xe. *J. Comput. Chem.* **2020**, *41* (20), 1842-1849. DOI: https://doi.org/10.1002/jcc.26355.
(37) Aravena, D.; Neese, F.; Pantazis, D. A. Improved Segmented All-Electron Relativistically Contracted Basis Sets for the Lanthanides. *Journal of Chemical Theory and Computation* **2016**, *12* (3), 1148-1156. DOI: 10.1021/acs.jctc.5b01048.
(38) Mitoraj, M.; Michalak, A. Donor–Acceptor Properties of Ligands from the Natural Orbitals for Chemical Valence. *Organometallics* **2007**, *26* (26), 6576-6580. DOI: 10.1021/om700754n.
(39) Lu, T.; Chen, Q. Independent gradient model based on Hirshfeld partition: A new method for visual study of interactions in chemical systems. *J. Comput. Chem.* **2022**, *43* (8), 539-555. DOI: https://doi.org/10.1002/jcc.26812.
(40) Bridgeman, A. J.; Cavigliasso, G.; Ireland, L. R.; Rothery, J. The Mayer bond order as a tool in inorganic chemistry*J. Chem. Soc., Dalton Trans.* **2001**, (14), 2095-2108, 10.1039/B102094N. DOI: 10.1039/B102094N.
